# Understanding Bio-Orthogonal Strain-Driven Sydnone Cycloadditions: Data-Assisted Profiles and the Search for Linear Relationships

**DOI:** 10.3390/molecules30132770

**Published:** 2025-06-27

**Authors:** Juan García de la Concepción, Pedro Cintas, Rafael Fernando Martínez

**Affiliations:** Departamento de Química Orgánica e Inorgánica, Facultad de Ciencias, Instituto Universitario de Investigación del Agua, Cambio Climático y Sostenibilidad (IACYS), Universidad de Extremadura, Avenida de Elvas s/n, 06006 Badajoz, Spain; pecintas@unex.es

**Keywords:** bio-orthogonal chemistry, cycloalkynes, free-energy linear relationships, mesoionic heterocycles, strain-promoted cycloadditions, sydnones

## Abstract

In the realm of click-type reactions and their application to bioorthogonal chemistry in living organisms, metal-free [3+2] cycloadditions involving mesoionic rings and strained cycloalkynes have gained increasing attention and potentiality in recent years. While there has been a significant accretion of experimental data, biological assays, and assessments of reaction mechanisms, some pieces of the tale are still missing. For instance, which structural and/or stereoelectronic effects are actually interlocked and which remain unplugged. With the advent of data-driven methods, including machine learning simulations, quantitative estimations of relevant observables and their correlations will explore better the chemical space of these transformations. Here we unveil a series of linear relationships, such as Hammett-type correlations, as well as deviations of linearity, using the case study of phenylsydnone (and its 4-aryl-substituted derivatives) with a highly reactive bicyclo[6.1.0]nonyne carbinol. Through accurate estimation of activation barriers and prediction of rate constants, our findings further increase the significance of integrating strain release and electronic effects in organic reactivity. Moreover, such results could pave the way to use mesoionics cycloadditions as probes for measuring the extent of delocalization-assisted strain release, which can be applied to related reactions involving dipoles and strained rings.

## 1. Introduction

There is little doubt that, since the early 21st century, bio-orthogonal chemistry has become one of the most innovative paradigms interfacing chemistry, biology, and even medicinal research. In short, bio-orthogonal reactions are designed to occur in biological environments (from cells to tissues and animal models) without interfering with native processes, thus enabling applications in biomolecular labeling, bioimaging, cell surface modification, or drug delivery, among others [1,2,3,4,5,6,7,8,9,10]. A series of synthetic transformations are expected to be orthogonal enough to enable chemoselective processes in vivo, which include variations of some classical reactions such as Staudinger ligation involving azides and a phosphine plus an acyl donor, azide–alkyne dipolar cycloadditions, or inverse electron-demand Diels–Alder cycloadditions of tetrazines and alkynes. Rooted in often metal-catalyzed regioselective click reactions like the Cu(I)-catalyzed azide-alkyne cycloaddition, a notable improvement was introduced by Bertozzi and associates who replaced standard alkynes by sterically hindered cycloalkyne surrogates, leading to metal-free strain-promoted bio-orthogonal reactions. The absence of metal traces should be much more biocompatible and avoids potential side effects. In context, fast reactions represent another key requirement for biological compatibility and viability, as reactions should be occurring at physiological conditions, i.e., mild temperature and near-neutral pH values. Certainly, as evidenced by recent studies, bio-orthogonal reactions may not exhibit complete chemoselectivity to enable site-selective bioconjugation, thereby affording an array of non-innocent transformations with biomolecules [11].

**Figure 1:**
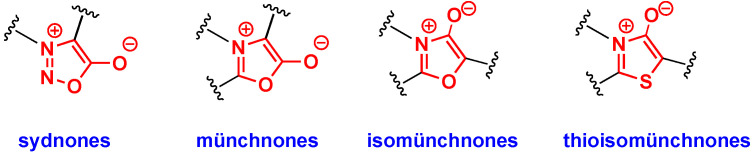
Representative subclasses of common mesoionic dipoles. Only one canonical structure is shown in every case.

In more recent times, another class of non-classical dipoles, mesoionic rings, have been incorporated into the repertoire of regioselective click cycloadditions. Introduced for the first time in the late 1940s, the structural nature and appropriate formulation of mesoionic systems have been both challenging and confusing [12,13]. Mesoionic rings are five-membered heterocycles that cannot be represented by Lewis structures without charge separation. IUPAC now considers mesoionic systems as a subclass of mesomeric betaines, which could then include six-membered rings as well [14]. The so-called nicknamed sydnones (1,2,3-oxadiazolium-5-olates), münchnones (1,3-oxazolium-5-olates), isomünchnones (1,3-oxazolium-4-olates), or thioisomünchnones (1,3-thiazolium-4-olates) constitute typical examples of well-explored mesoionic heterocycles (Figure 1). Being familiar with their chemistry for nearly three decades, we and others have long showcased both the conventional and unexpected pathways performed by such masked dipoles [15,16,17,18,19,20].

Mesoionics have the ability of participating as willing dipoles in atom-economical [3+2] cycloadditions. In numerous cases, the resulting intermediate cycloadducts undergo subsequent ring-opening accompanied by extrusion of small molecules and leading to novel heterocycles. As expected, the click-and-release tandem cycloadditions of mesoionics make them attractive synthons for bio-orthogonal chemistry in living cells, a field particularly developed by Taran and coworkers with sydnones and iminosydnones [21,22,23].

It is likely the case that one of the most recurring and controversial issues is whether mesoionic rings are or not aromatic species. While a sextet of electrons prone to delocalization can be identified, different types of conjugation, cross-conjugation, and hyperconjugation are plausible [12,13]. Current views, supported by solid-state characterization and theoretical analyses, suggest that extended delocalization takes place, which can also be tuned by push–pull effects of the heteroatoms and the substitution pattern [24]. The point is pertinent in the light of the emerging conceptual framework of delocalization-assisted strain release enabling directed reactivity [25]. Clearly, interactions of varied dipoles with cycloalkynes illustrate this dominant effect well.

In a previous study, the energy barriers and saddle points of some cycloadditions involving cycloalkynes and mesoionics, sydnone derivatives in particular, were computationally evaluated, which led to some predictive guidelines. Reactivity appears to be dictated by electron flux from the alkyne to the dipole, and sustained by non-covalent interactions, which can also be supported by charge transfer (CT) analyses [26].

**Scheme 1:**
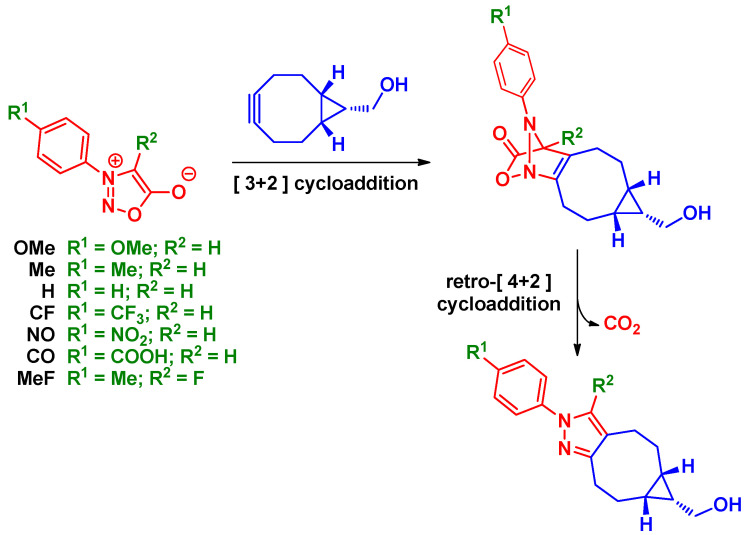
Tandem [3+2]/retro-[4+2] cycloadditions of sydnones and BCN leading to pyrazoles.

In order to shed light into the intimate nature of such strain-driven mesoionics-based cycloadditions and the influence, if any, of stereoelectronic effects, an in-depth evaluation of potential correlations has been carried out. To this end, thermodynamic and kinetic analyses have been computed at high levels for the reaction of phenyl sydnone, alongside its 4-substituted derivatives, with a widely employed hydroxymethylene-substituted bicyclononyne, namely (1*R*,8*S*,9*S*)-bicyclo[6.1.0]non-4-yn-9-yl methanol (BCN). The choice of BCN responds to a series of inherent advantages, namely, facile preparation (four steps from 1,5-cyclooctadiene), facile synthetic functionalization, and its symmetric structure prevents the existence of regioisomeric side products. In addition, it exhibits a good balance between reactivity and hydrophobicity (the latter suitable for potential in vivo labeling), relative to other strained alkynes [27,28]. As displayed in Scheme 1, the resulting cycloadduct is not stable enough and undergoes a spontaneous retro-Diels–Alder reaction that releases a pyrazole ring and ${\mathrm{CO}}_{2}$ as the sole byproduct.

## 2. Materials and Methods

### 2.1. Electronic Structure Calculations

All electronic structure calculations were carried out with the Gaussian 16 software package (revision A.03) [29]. All geometries were optimized with the hybrid meta-GGA functional M06-2X [30] in combination with the 6-311++G(d,p) basis set [31], with inclusion of solvation effects in water using the SMD method [32,33]. The M06-2X functional was chosen, because it provided good overall performance in describing pericyclic reactions according to a recent and comprehensive analysis that benchmarked 60 density functionals (only eight dispersion-corrected) with optimized geometric structures and frequencies of all stationary points at the CCSDT(Q)/CBS levels [34]. The meta-hybrid M06-2X functional afforded the best overall performance (with mean absolute errors of ca. 1 kcal/mol), while remarkably, the use of otherwise well-established GGA functionals provided poorer results (with mean absolute errors up to 5.8 kcal/mol), even if they can quantitatively describe both reaction barrier and energy profiles. Based on these considerations, as the present study involves two sequential cycloadditions, the aforementioned functional without further dispersion corrections was employed. All the stationary points were characterized by frequency calculations at the above-mentioned level of theory, showing none and one imaginary frequencies for energy minima and transition structures (TSs), respectively. A frequency scale factor of 0.970 was used to correct zero point vibrational energy (ZPVE) [35]. Hirshfeld atomic charges [36] were computed with the Multiwfn 3.8 software [37,38]. All saddle points linking specific reactant and products through the reaction path were verified using intrinsic reaction coordinate (IRC) analysis.

### 2.2. Conformational Analysis of BCN

**Figure 2:**
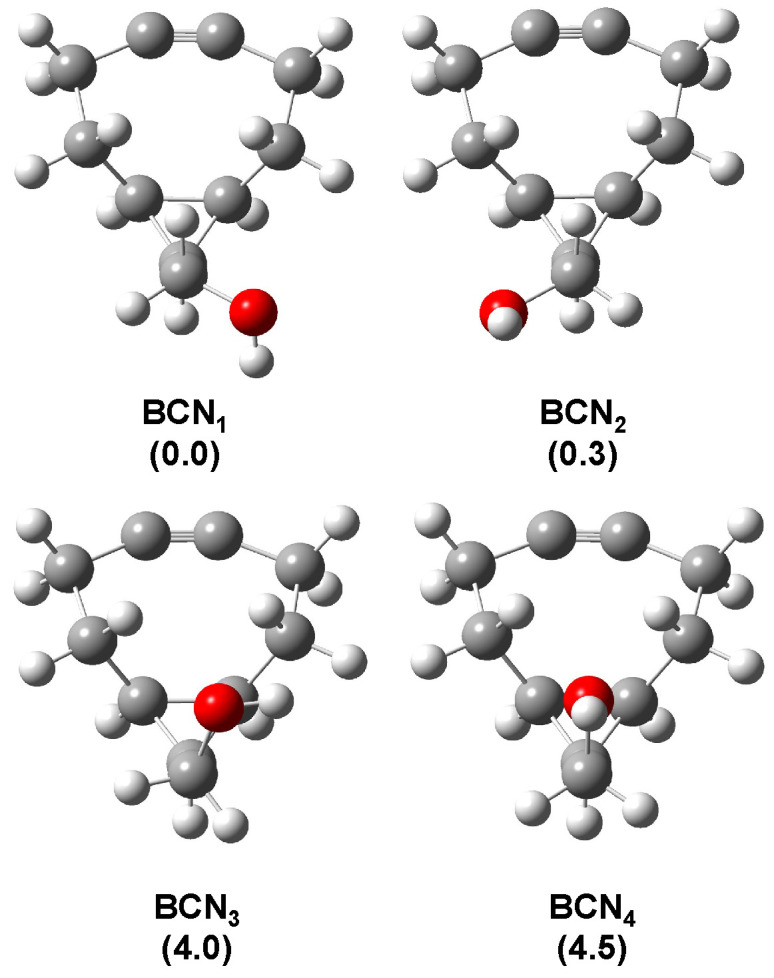
Optimized geometries for the four conformers of BCN at the M06-2X/6-311++G(d,p) level of theory as modeled with bulk solvation in water (SMD method). Values in parenthesis are the relative free energies in kcal ${\mathrm{mol}}^{-1}$ relative to the most stable conformer at 298.15 K.

The conformational analysis performed for BCN was carried out based on meta molecular dynamics simulations (MTD) within the NCI-iMTD workflow, implemented in the CREST software (version 3.0.1) [39] with the GFN2-xTB semiempirical method [40]. Within a six kcal ${\mathrm{mol}}^{-1}$ window, four conformers could be located, which were re-optimized and characterized as energy minima at the above-mentioned DFT level of theory. Two out of the four conformers $({\mathrm{BCN}}_{1}$ and ${\mathrm{BCN}}_{2})$ showed a free energy difference of 0.3 kcal ${\mathrm{mol}}^{-1}$. One of them $\left({\mathrm{BCN}}_{3}\right)$ was 4.0 kcal ${\mathrm{mol}}^{-1}$ above the most stable conformer, while the fourth $\left({\mathrm{BCN}}_{4}\right)$ was 4.5 kcal ${\mathrm{mol}}^{-1}$ higher (Figure 2).

### 2.3. Kinetic Calculations

The reaction mechanism accounting for the formation of the pyrazole ring from sydnone and BCN reveals that the bottleneck involves the transition structure corresponding to the 1,3-dipolar cycloaddition. Then, the rate determining step approximation for obtaining the rate constants was applied using the Eyring equation:

(1) $$k\left(T\right)=\sigma_{react}\kappa \frac{k_{B}T}{h}\frac{1}{C^{o}}e^{\frac{-\Delta G^{\mathrm{SP}}}{RT}}$$

where $\Delta G^{SP}$ is the relative free energy of the saddle point with respect to the reactants, $\sigma_{react}$ is the reaction path degeneracy, which was set to one (see below), κ is the transmission coefficient set to 1 as well, $k_{B}$ and *h* are the Boltzmann and Plank constants, respectively, *R* is the ideal gas constant, *T* is the temperature, which was set to 298.15 K, and $C^{o}$ is the standard concentration in mol $L^{-1}$ (M).

Bearing in mind that the most stable conformers of BCN are separated by only 0.3 kcal ${\mathrm{mol}}^{-1}$, both species should be considered in the kinetic calculations, since at 298.15 K they are supposed to be in equilibrium with a $K_{eq}$ of 0.61. For each conformer, there are two degenerate rotamers that do not give a doubly degenerated reaction pathway ($\sigma_{react}$ = 2), since the sydnone is not symmetrically substituted, giving rise to two different conformational arrangements in the transition structure when encountering either BCN conformer. Due to free rotation occurring at 298.15 K, all the conformers lead to the same product. Accordingly, four TSs should be considered for each sydnone with the two lowest BCN conformers. Moreover, at the TSs, the cyclopropane ring could adopt either *syn* or anti dispositions with respect to the aryl group of the sydnone moiety. The flat geometry of the pyrazole ring ensures that after the loss of ${\mathrm{CO}}_{2}$, both syn- and anti-configured TSs lead to the same product. Overall, for each sydnone, eight TSs should be taken into account to perform the corresponding kinetic calculations.

For a $K_{eq}$ of 0.61, fractional populations of 0.62 and 0.38 are obtained for the most and least stable conformers, respectively. Then, the global rate constants are given by

(2) $$k_{\mathrm{tot}}\left(T\right)=0.62\sum_{i=1}^{n}k{\left(T\right)}_{i}^{{\mathrm{BCN}}_{1}}+0.38\sum_{i=1}^{n}k{\left(T\right)}_{i}^{{\mathrm{BCN}}_{2}}$$

where ${\mathrm{BCN}}_{1}$ and ${\mathrm{BCN}}_{2}$ represent the most stable conformers of BCN. For each, $n_{max}$ is 4, i.e., two rotamers of BCN that are degenerated in the reactant asymptote and two corresponding to the above-mentioned syn and anti dispositions of the cyclopropane ring with respect to the aryl group in the cycloaddition reaction.

All the calculated rate constants are overestimated compared to the experimental results by no more than a factor of eight. Thus, in order to reproduce the experimental results, we applied a correction factor for all the rate constants as

(3) $$F=\frac{1}{n}\sum_{i=1}^{n}\left(\frac{k_{\exp,i}}{k_{\mathrm{tot},i}\left(T\right)}\right)$$

where *n* is the number of sydnones depicted in Scheme 1, $k_{exp}$ is the experimental rate constant, and $k_{tot}\left(T\right)$ is the global rate constant computed for each sydnone obtained from Equation (2). As a result, our final rate constants are given by

(4) $$k_{\mathrm{theo}}\left(T\right)=Fk_{\mathrm{tot}}\left(T\right)$$

## 3. Results and Discussion

As indicated in Section 1, the main purpose of this analysis was to find quantitative structure–reactivity relationships for the cycloaddition of sydnones with BCN looking for a given parameter of the former that allows experiments by design without the usual trial-and-error protocol in the lab. To this end, the reactivity of a series of sydnones with BCN, for which experimental data are available [41,42], was interrogated theoretically. These tandem reactions yield pyrazole rings and ${\mathrm{CO}}_{2}$ as byproduct, as shown in Scheme 1.

### 3.1. Conformations of BCN

Despite its structural rigidity, the well-known dipolarophile BCN has rotatable sigma bonds, and hence a conformational screening of all the potential reactive conformers appears to be compulsory. Using the computational details mentioned in Section 2.2 and within a range of 6 kcal ${\mathrm{mol}}^{-1}$ window, four conformers whose optimized geometries and relative free energies are shown in Figure 2 could be detected. It is noteworthy that each conformer shows a degenerate partner not shown (apart from ${\mathrm{BCN}}_{4}$, which possesses a plane of symmetry through the OH group). Both ${\mathrm{BCN}}_{1}$ and ${\mathrm{BCN}}_{2}$ should be considered for assessing the kinetics of the cycloaddition as they are interconvertible under the given experimental conditions. Furthermore, they are very close in energy, showing a Boltzmann factor of 0.61 at 298.15 K.

### 3.2. Reaction Mechanism for the Reaction of BCN and Sydnone **H**

1,3-Dipolar reactions, formally [3+2] cycloadditions of allyl-type dipoles and alkenes, can follow either concerted or stepwise mechanisms [43]. This dichotomy, even among different reaction channels, often takes place with mesoionic rings in view of their subtle dipolar character [44]. Accordingly, this key point was the subject of our preliminary investigation with a focus on the simplest sydnone **H** (Scheme 1).

**Figure 3:**
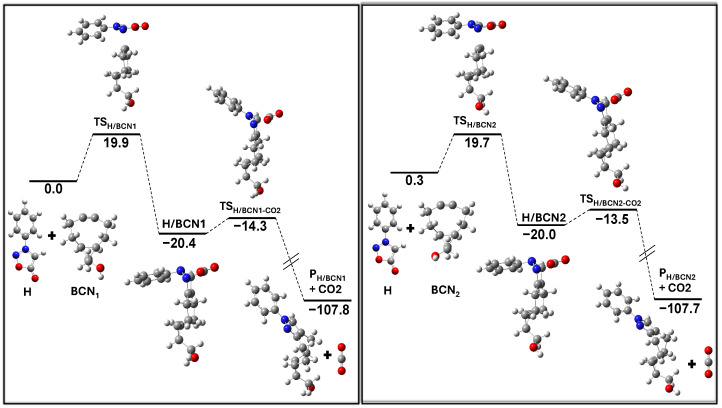
Free energy profiles at 298.15 K for the reaction of sydnone **H** with ${\mathrm{BCN}}_{1}$ (**left**) and ${\mathrm{BCN}}_{2}$ (**right**). Free energies are given in kcal ${\mathrm{mol}}^{-1}$. Color code: carbon (grey), hydrogen (white), nitrogen (blue), oxygen (red).

The free energy profiles for the reaction pathways of **H** and ${\mathrm{BCN}}_{1}$ or ${\mathrm{BCN}}_{2}$ are shown in Figure 3. Both reaction paths show almost identical free energy profiles since the ${\mathrm{CH}}_{2}\mathrm{OH}$ moiety of the dipolarophile is quite distant from the reacting bonds involved, thus exerting little or no influence. Certainly, the computational analysis unveils the concertedness of the first step involving the 1,3-dipolar cycloaddition between the reactants (${\mathrm{TS}}_{H/BCN1}$ and ${\mathrm{TS}}_{H/BCN2})$, showing energy barriers of 19.9 and 19.7 kcal ${\mathrm{mol}}^{-1}$ for ${\mathrm{BCN}}_{1}$ and ${\mathrm{BCN}}_{2}$, respectively. The transition structures lead to the strained cycloadducts **H/BCN1** and **H/BCN2** (−20.4 and −20.0 kcal ${\mathrm{mol}}^{-1}$, respectively). The subsequent step is the rapid and spontaneous loss of ${\mathrm{CO}}_{2}$ from the cycloadducts through a retro-Diels–Alder cycloaddition leading to the pyrazole rings $P_{H/BCN1}$ or $P_{H/BCN2}$. These steps correspond with the transition structures ${\mathrm{TS}}_{H/BCN1-CO2}$ and ${\mathrm{TS}}_{H/BCN2-CO2}$, which show relative free energies of −14.3 and −13.5 kcal ${\mathrm{mol}}^{-1}$, respectively.

### 3.3. Calculation of Rate Constants

**Figure 4:**
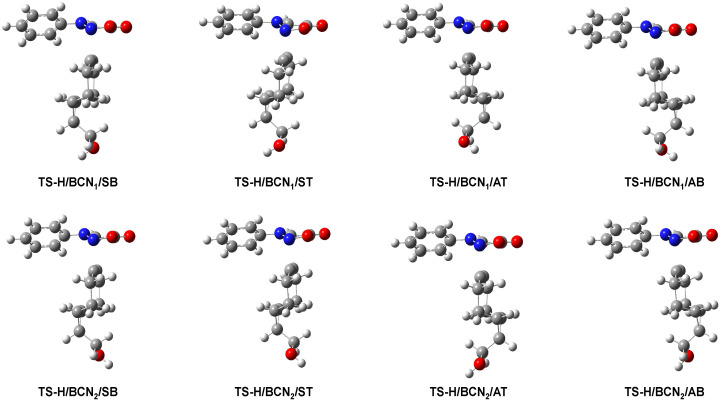
Optimized geometries of the eight TSs for the reaction between **H** and BCN at the M06-2X/6-311++G(d,p) level of theory in water (SMD). The nomenclature is as follows: ${\mathrm{BCN}}_{1}$ and ${\mathrm{BCN}}_{2}$ refer to the two conformers of BCN, **S** and **A** refer to the syn and anti orientations of the cyclopropane ring with respect to the aryl group of the parent sydnone, and **T** and **B** represent the top and bottom dispositions of the OH with respect to the plane. Color code: carbon (grey), hydrogen (white), nitrogen (blue), oxygen (red).

The inspection of the reaction mechanism allows us to restrict the kinetic calculations to the rate-determining step approximation because the cycloaddition represents actually the bottleneck of the whole transformation (see Figure 3). However, additional considerations should be taken into account, as transition structures other than those depicted in Figure 3 could lead to the same pyrazole ring. As mentioned above, conformers ${\mathrm{BCN}}_{1}$ and ${\mathrm{BCN}}_{2}$ equilibrate via a equilibrium constant of 0.61 and the two conformational dispositions will also be present in the resulting pyrazole, affording an identical product. For each BCN, there are two degenerate geometries that lose their degeneration in the TSs because the parent sydnones are not symmetrically substituted. The corresponding syn and anti arrangements of the cyclopropane fragment with respect to the aryl group account for four additional TSs. They are depicted in Figure 4 and denoted as **TS-H/BCN1/SB** and **TS-H/BCN1/ST** for the two degenerate conformers of ${\mathrm{BCN}}_{1}$ with the cyclopropane ring for the syn orientation, and as **TS-H/BCN1/AB** and **TS-H/BCN1/AT** for the anti orientation. The same applies to the cycloaddition with ${\mathrm{BCN}}_{2}$. All the relative energies of the transitions structures for each sydnone are gathered in Tables S1–S7.

Considering the above-mentioned approach, eight rate constants for each sydnone, four for ${\mathrm{BCN}}_{1}$ and four for ${\mathrm{BCN}}_{2}$ (Tables S1–S7), were calculated with Equation (1). $k_{tot}\left(T\right)$ was then computed with Equation (2), which includes the population ratios of both conformers. All these calculated rate constants overestimated the experimental results, as can be inferred from the data shown in Table 1. The mesoionic dipoles showing large deviations ($k_{tot}\left(T\right)$) from the experimental values were the sydnones **OMe** and **CO**, by a factor of 7.9 and 5.1, respectively.

### 3.4. Relationship of Electronic Effects with Rate Constants

Since the calculated rate constants showed good agreement with the experimental results, a series of linear relationships were explored with the aim of rationalizing the experimental data and eventually proposing quantitative predictions.

A priori, the electronic effect of substituents, namely Hammett plots, provide the most common LFER (linear free energy relationship) by examining changes in charges during the reaction mechanism. The electronic effects of the substituents attached at the *para* position of the phenyl ring for six *N*-phenyl sydnones (**OMe**, **Me**, **H**, **CF**, **NO**, and **CO**), exploring Hammett-type correlations with rate constants, dipole moments, and the energy values of the LUMO orbital of sydnones, were investigated (Table S8). Fluorosydnone **MeF** was not explicitly considered, since it bears a fluorine atom at Position 5, nor could sydnone **MeF** be included in the correlation analyses, as no electrical property exhibited any significant variation (i.e., increase/decrease) with the rate constant, unlike the other sydnones.

**Figure 5:**
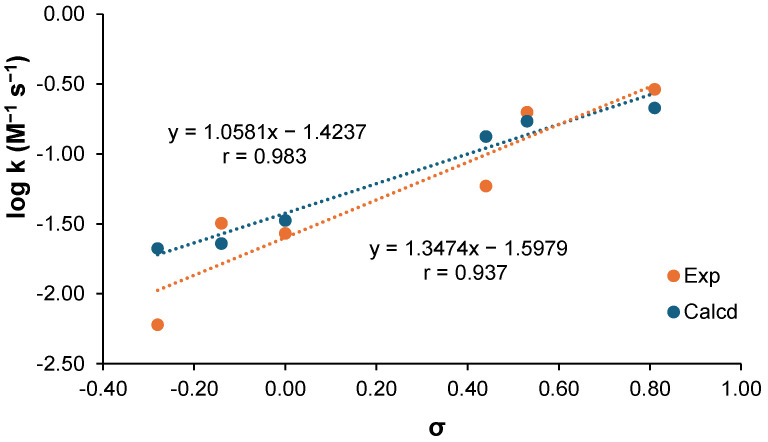
Hammett-type plots between experimental and calculated rate constants against σ values.

Hammett-type plots between both experimental and calculated rate constants versus σ values (Hammett constant) led to highly linear relationships (Figure 5). These results are consistent with a Type III Sustmann cycloaddition through an inverse electron-demand mechanism, as previously reported by Houk and coworkers [45]. Thus, the presence of electron-withdrawing groups at the phenyl ring of the syndnone would enhance the reaction rate, which would be beneficial for the bio-orthogonal ligation. Moreover, this linear relationship foresees the influence of operative electronic effects, ranging from polarizability, field and inductive, or resonance contributions on reactivity, by a straightforward correlation with the Hammett constant σ for a given substituent at the phenyl ring of the sydnone partner as showns by Equation (5):

(5) $$log(k_{exp})=1.03474\sigma +1.5979$$

**Figure 6:**
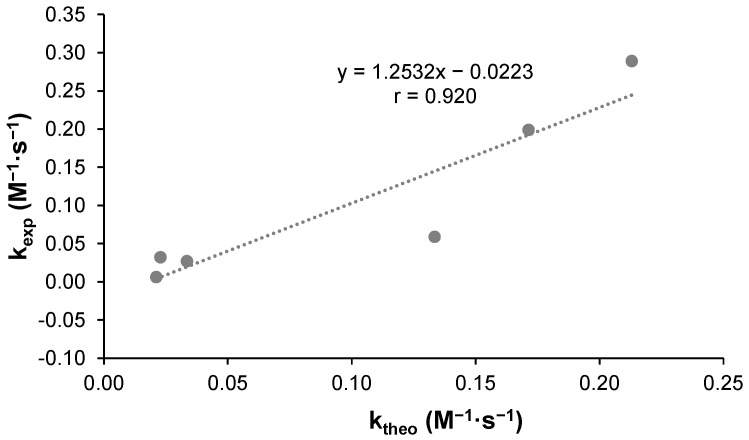
Linear plot of experimental rate constants versus calculated values from Equation (4).

Remarkably, a good correlation could also be obtained when the experimental rate constants were plotted versus the calculated values from Equation (4) (Figure 6), thus reinforcing the predictive character of this approach.

At first sight, enhanced reactivity of 1,3-dipolar cycloadditions involving low-LUMO lying partners could be expected. However, the inspection of frontier orbital energies do not provide an immediate rationale unless other factors such as orbital or distortion effects are involved. Thus, secondary orbital interactions do apparently account for the reactivity of nonynes (like unsubstituted BCN) in reactions with tetrazines [46], which also represent a case of inverse electron-demand Diels–Alder reaction.

**Figure 7:**
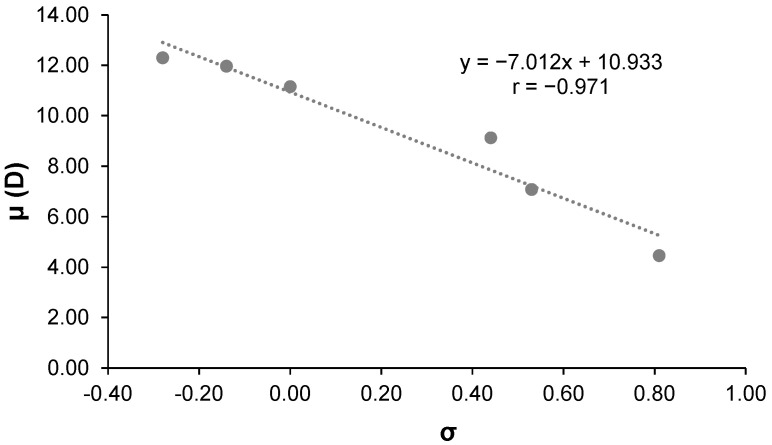
Linear relationship between the dipole moment of sydnones and σ values.

**Figure 8:**
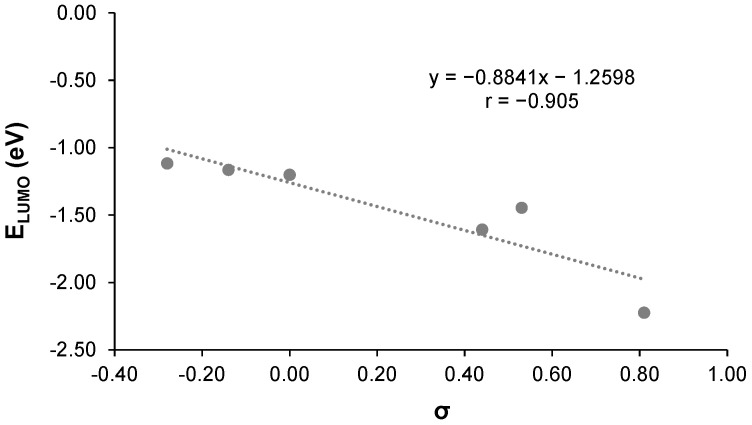
Linear relationship between the energy value of the sydnone LUMO orbital and σ values.

**Figure 9:**
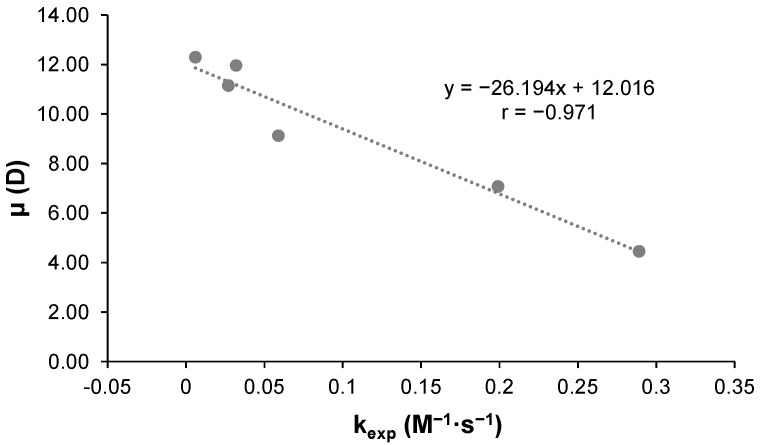
Linear representation between the dipole moment of sydnones and the experimental rate constants.

To estimate further how the dipolar nature of mesoionic heterocycles influence their reactivity, the electronic effect of the substituents at the phenyl ring was also correlated to the dipole moment of sydnones (Figure 7) as well as to the energy of the corresponding LUMO orbital (Figure 8), obtaining good regression coefficients in both cases. Notably, five-membered mesoionics show high permanent dipole moments. Although the enhanced reactivity could be ascribed to diradical behavior, and mesoionic carbenoids can easily be generated by metalation, the metastable character is most likely due to repulsive effects within the inner heterocyclic structure [47]. Likewise, the plot of the dipole moment of sydnones against the corresponding experimental reaction rate values led to an excellent linear relationship (Figure 9), and predictions may be inferred from the calculated dipole moment of a particular sydnone (Equations (6) and (7)).

(6) $$\mu =-26.194k_{exp}+12.016$$

(7) $$k_{exp}=\frac{12.016-\mu }{26.194}$$

These representations demonstrate the strong influence that the electronic properties of substituents at the *para* position of the phenyl ring have on the kinetics of the reaction of *N*-aryl sydnones with BCN.

**Figure 10:**
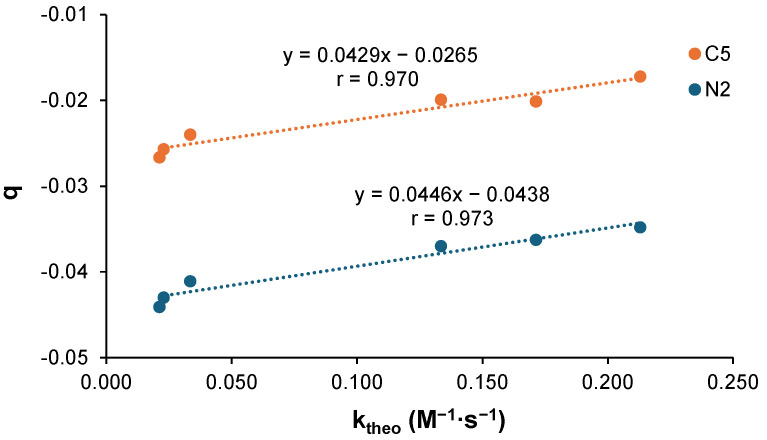
Linear correlations between calculated Hirshfeld charge values at C5 (orange)/N2 (blue) of the sydnone ring and the calculated rate constants.

**Figure 11:**
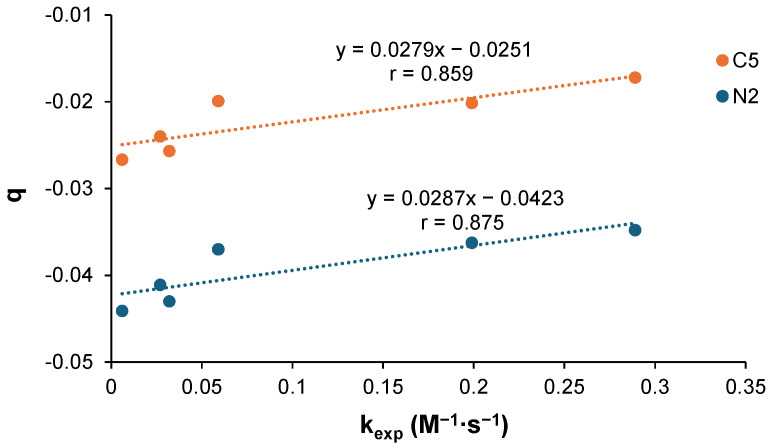
Linear correlations between calculated Hirshfeld charge values at C5 (orange)/N2 (blue) of the sydnone ring and the experimental rate constants.

Finally, high correlations were obtained as well when the calculated charge values at Positions N2 and C5 of sydnones were plotted against the calculated rate constants (Figure 10). However, the same representation taking the experimental rate constants provided a modest correlation for both N2 and C5 atoms (Figure 11). It is well known that Positions 2 and 5 at the sydnone ring are the most reactive sites in the cycloaddition reaction with BCN. Therefore, the charge developed by these two atoms should heavily be influenced by the mesomeric effect of the substituent at the aromatic ring of sydnones, and hence will have a significant impact on the reaction rate.

## 4. Conclusions

We have disclosed in detail that electronic changes that can be viewed as substituent effects nicely fit the concept of a structure–reactivity relationship in sydnone-based cycloadditions with strained alkynes. These processes are now recognized as a salient addendum to bio-orthogonal chemistry capable of liberating in vivo small molecules of diagnostic or signaling significance. Mesoionic 1,3-dipolar cycloadditions can also be regarded as *dipole-transmissive* reactions, by virtue of a modularity that enables the construction/deconstruction/reorganization of several bonds and cycles in a few steps [48]. Taking as examples the cycloadditions of aryl-substituted sydnones and a bicyclononyne carbinol, whose mechanistic and stereochemical features have been thoroughly disentangled, both energy barriers and reaction rates have now been determined with high precision.

Through Hammett plots involving experimental and computed rates, the effect of electronic parameters upon the reactivity of substituted sydnones undergoing change has been proven. It is obvious that, on the one hand, other polar and steric effects should be explored in the near future and that, on the other, the possibility of accessing heteroatomic mesoionics other than sydnones or iminosydnones, like sydnthiones whose bio-orthogonal chemistry has recently begun [49], could furnish different correlations. Therefore, the present analysis opens the door to linear free energy relationships for these kinds of substituent effects in orthogonal cycloadditions with mesoionic dipoles.

## Figures and Tables

**Table 1:** Experimental ($k_{exp}$) and theoretical rate constants ($k_{tot}\left(T\right)$ and $k_{theo}\left(T\right)$) in $M^{-1}s^{-1}$.

Sydnone	$k_{\exp}$	$k_{\mathrm{tot}}\left(T\right)$	$k_{\mathrm{theo}}\left(T\right)$
**OMe**	0.006	0.048	0.021
**Me**	0.032	0.052	0.023
**H**	0.027	0.075	0.033
**CO**	0.059	0.301	0.133
**CF**	0.199	0.386	0.171
**NO**	0.289	0.480	0.213
**MeF**	42.0	61.0	27.1

## Data Availability

Additional data, as mentioned in this manuscript, are provided in the Supplementary Materials. Further information can be obtained from the authors upon reasonable request.

## Supplementary Materials

The following supporting information can be downloaded at https://www.mdpi.com/article/10.3390/molecules30132770/s1. References [41,50] are cited in the supplementary materials.
