# Supplementary material for: Understanding Bio-Orthogonal Strain-Driven Sydnone Cycloadditions: Data-Assisted Profiles and the Search for Linear Relationships
Source: Molecules. 2025 Jun 27;30(13):2770. doi: 10.3390/molecules30132770 (PMC12251426; doi:10.3390/molecules30132770)
Supplement: Supplementary file 1 [file molecules-30-02770-s001.zip › molecules-3702577-supplementary.pdf]

Understanding Bio-orthogonal Strain-Driven Sydnone Cycloadditions: Data-Assisted Profiles and the Search for Linear Relationships

Juan García de la Concepción*, Pedro Cintas and R. Fernando Martínez*

Departamento de Química Orgánica e Inorgánica, Facultad de Ciencias, and Instituto Universitario de Investigación del Agua, Cambio Climático y Sostenibilidad, (IACYS), Universidad de Extremadura, Avenida de Elvas s/n, 06006 Badajoz, Spain;

* Correspondence: rmarvaz@unex.es (R.F.M.); jugarco@unex.es (J.G.dlC)

Page	Supplementary Data
S2	Tables S1-S8.
S6	Computational data for optimized structures.

Table S1. Calculated rate constants from the rate-limiting step for the cycloaddition between sydnone **H** and BCN.

	**ΔG^{SP}** (kcal mol^{-1})	**k** (M^{-1} s^{-1}) [a]
**TS-H/BCN$_1$/AT**	19.91	0.016
**TS-H/BCN$_1$/AB**	20.04	0.013
**TS-H/BCN$_1$/ST**	19.89	0.017
**TS-H/BCN$_1$/SB**	19.87	0.017
**TS-H/BCN$_2$/AB**	20.02	0.014
**TS-H/BCN$_2$/AT**	19.73	0.022
**TS-H/BCN$_2$/SB**	19.73	0.022
**TS-H/BCN$_2$/ST**	19.42	0.037
	$k_{tot}(T)$	0.075

[a] Calculated from equation 1 (see manuscript).

Table S2. Calculated rate constants from the rate-limiting step for the cycloaddition between sydnone **OMe** and BCN.

	**ΔG^{SP}** (kcal mol^{-1})	**k** (M^{-1} s^{-1}) [a]
**TS-OMe/BCN$_1$/AT**	20.38	0.007
**TS-OMe/BCN$_1$/AB**	20.36	0.008
**TS-OMe/BCN$_1$/ST**	20.11	0.012
**TS-OMe/BCN$_1$/SB**	20.44	0.007
**TS-OMe/BCN$_2$/AB**	20.21	0.010
**TS-OMe/BCN$_2$/AT**	20.18	0.010
**TS-OMe/BCN$_2$/SB**	19.88	0.017
**TS-OMe/BCN$_2$/ST**	19.48	0.034
	$k_{tot}(T)$	0.048

[a] Calculated from equation 1 (see manuscript).

Table S3. Calculated rate constants from the rate-limiting step for the cycloaddition between sydnone **Me** and BCN.

	**ΔG^SP^** (kcal mol $^{-1}$)	**k** (M^{-1} s^{-1}) [a]
**TS-Me/BCN$_1$/AT**	20.12	0.011
**TS-Me/BCN$_1$/AB**	20.68	0.004
**TS-Me/BCN$_1$/ST**	20.02	0.014
**TS-Me/BCN$_1$/SB**	20.26	0.009
**TS-Me/BCN$_2$/AB**	19.79	0.020
**TS-Me/BCN$_2$/AT**	19.64	0.026
**TS-Me/BCN$_2$/SB**	19.84	0.018
**TS-Me/BCN$_2$/ST**	20.28	0.009
	$k_{tot}(T)$	0.052

[a] Calculated from equation 1 (see manuscript).

Table S4. Calculated rate constants from the rate-limiting step for the cycloaddition between sydnone **CO** and BCN.

	**ΔG^SP^** (kcal mol $^{-1}$)	**k** (M^{-1} s^{-1}) [a]
**TS-CO/BCN$_1$/AT**	19.48	0.034
**TS-CO/BCN$_1$/AB**	19.21	0.054
**TS-CO/BCN$_1$/ST**	18.83	0.101
**TS-CO/BCN$_1$/SB**	18.92	0.087
**TS-CO/BCN$_2$/AB**	18.88	0.093
**TS-CO/BCN$_2$/AT**	19.37	0.041
**TS-CO/BCN$_2$/SB**	18.89	0.091
**TS-CO/BCN$_2$/ST**	18.74	0.118
	$k_{tot}(T)$	0.301

[a] Calculated from equation 1 (see manuscript).

Table S5. Calculated rate constants from the rate-limiting step for the cycloaddition between sydnone **CF** and BCN.

	**ΔG^{SP}** (kcal mol^{-1})	**k** (M^{-1} s^{-1}) [a]
**TS-CF/BCN$_1$/AT**	19.54	0.030
**TS-CF/BCN$_1$/AB**	19.56	0.029
**TS-CF/BCN$_1$/ST**	18.94	0.084
**TS-CF/BCN$_1$/SB**	18.37	0.220
**TS-CF/BCN$_2$/AB**	18.72	0.121
**TS-CF/BCN$_2$/AT**	19.27	0.048
**TS-CF/BCN$_2$/SB**	18.60	0.148
**TS-CF/BCN$_2$/ST**	18.80	0.106
	$k_{tot}(T)$	0.386

[a] Calculated from equation 1 (see manuscript).

Table S6. Calculated rate constants from the rate-limiting step for the cycloaddition between sydnone **NO** and BCN.

	**ΔG^{SP}** (kcal mol^{-1})	**k** (M^{-1} s^{-1}) [a]
**TS-NO/BCN$_1$/AT**	18.85	0.097
**TS-NO/BCN$_1$/AB**	18.92	0.087
**TS-NO/BCN$_1$/ST**	19.09	0.065
**TS-NO/BCN$_1$/SB**	18.86	0.096
**TS-NO/BCN$_2$/AB**	18.59	0.150
**TS-NO/BCN$_2$/AT**	18.79	0.108
**TS-NO/BCN$_2$/SB**	18.69	0.127
**TS-NO/BCN$_2$/ST**	18.15	0.318
	$k_{tot}(T)$	0.480

[a] Calculated from equation 1 (see manuscript).

Table S7. Calculated rate constants from the rate-limiting step for the cycloaddition between sydnone **MeF** and BCN.

	**ΔG^{SP}** (kcal mol^{-1})	**k** (M^{-1} s^{-1}) [a]
**TS-MeF/BCN$_1$/AT**	15.92	13.8
**TS-MeF/BCN$_1$/AB**	16.25	7.9
**TS-MeF/BCN$_1$/ST**	16.35	6.6
**TS-MeF/BCN$_1$/SB**	15.84	15.7
**TS-MeF/BCN$_2$/AB**	15.82	16.2
**TS-MeF/BCN$_2$/AT**	15.71	19.5
**TS-MeF/BCN$_2$/SB**	15.86	15.1
**TS-MeF/BCN$_2$/ST**	15.31	38.5
	$k_{tot}(T)$	61.0

[a] Calculated from equation 1 (see manuscript).

Table S8. Electronic effect of the substituents at the aromatic ring of aryl sydnones on the cycloaddition reaction rate with BCN.

Sydnone	k_{exp} [a]	k_{theo} [b]	μ (D)	q_{N2} [c]	q_{C5} [d]	E_{LUMO} [e]	σ [f]
**OMe**	0.006	0.021	12.29	-0.0441	-0.0267	-1.117	-0.28
**Me**	0.032	0.023	11.96	-0.0430	-0.0257	-1.165	-0.14
**H**	0.027	0.033	11.15	-0.0411	-0.0240	-1.201	0.00
**CO**	0.059	0.133	9.13	-0.0370	-0.0199	-1.608	0.44
**CF**	0.199	0.171	7.07	-0.0363	-0.0201	-1.446	0.53
**NO**	0.289	0.213	4.45	-0.0348	-0.0172	-2.224	0.81

[a] Ref. 1; [b] Calculated rate constant according to equation 4 (see manuscript); [c] Calculated charge at N2 of the sydnone; [d] Calculated charge at C5 of the syndone; [e] Calculated energy of the LUMO orbital of the sydnone (in eV); [f] Hammett constant (ref. 2).

Computational data for optimized structures at the M06-2X/6-311++G(d,p) level in water (SMD method)

BCN1

```
Zero-point correction=                           0.220205 (Hartree/Particle)
Thermal correction to Energy=                    0.230814
Thermal correction to Enthalpy=                  0.231758
Thermal correction to Gibbs Free Energy=         0.184431
Sum of electronic and zero-point Energies=          -464.324272
Sum of electronic and thermal Energies=             -464.313663
Sum of electronic and thermal Enthalpies=           -464.312719
Sum of electronic and thermal Free Energies=        -464.360046
```

No imaginary frequencies

Standard orientation:

Center Number	Atomic Number	Atomic Type	Coordinates (Angstroms) X	Y	Z
1	6	0	2.518978	-0.166239	-0.238257
2	6	0	2.251150	1.008312	-0.184347
3	6	0	2.201068	-1.597605	-0.249773
4	1	0	2.657964	-2.117321	-1.093832
5	1	0	2.549661	-2.082392	0.666027
6	6	0	1.351448	2.159996	-0.067603
7	1	0	1.518001	2.897971	-0.854373
8	1	0	1.487418	2.664133	0.892922
9	6	0	-0.077227	1.567708	-0.174777
10	1	0	-0.801880	2.382501	-0.078412
11	1	0	-0.194359	1.144204	-1.174830
12	6	0	-0.357917	0.526803	0.894956
13	6	0	0.655581	-1.660018	-0.346996
14	1	0	0.345251	-2.709783	-0.357487
15	1	0	0.361822	-1.222477	-1.303376
16	6	0	-0.024898	-0.952986	0.813573
17	1	0	-0.247297	0.931067	1.897715
18	1	0	0.267606	-1.375172	1.771292
19	6	0	-1.464394	-0.494775	0.766203
20	1	0	-2.053229	-0.679403	1.660233
21	6	0	-2.273868	-0.605879	-0.498292
22	1	0	-1.662877	-0.470800	-1.389313
23	1	0	-2.737644	-1.596231	-0.558369
24	8	0	-3.288759	0.402537	-0.570690
25	1	0	-3.859874	0.301510	0.199200

BCN2

```
Zero-point correction=                           0.220220 (Hartree/Particle)
Thermal correction to Energy=                    0.230803
Thermal correction to Enthalpy=                  0.231747
Thermal correction to Gibbs Free Energy=         0.184623
Sum of electronic and zero-point Energies=          -464.323977
Sum of electronic and thermal Energies=             -464.313394
Sum of electronic and thermal Enthalpies=           -464.312450
Sum of electronic and thermal Free Energies=        -464.359574
```

No imaginary frequencies

Standard orientation:

Center Number	Atomic Number	Atomic Type	Coordinates (Angstroms) X	Y	Z
1	6	0	-2.234076	1.026034	-0.198477
2	6	0	-2.514553	-0.145235	-0.259108
3	6	0	-1.324052	2.167914	-0.067141
4	1	0	-1.472973	2.909822	-0.853770
5	1	0	-1.467191	2.670785	0.893009
6	6	0	-2.212006	-1.579959	-0.270717
7	1	0	-2.666789	-2.093138	-1.119913
8	1	0	-2.574311	-2.062565	0.640902
9	6	0	-0.666508	-1.658609	-0.354526
10	1	0	-0.367087	-2.711562	-0.366055
11	1	0	-0.360343	-1.220871	-1.306672
12	6	0	0.011059	-0.963374	0.814842
13	6	0	0.099902	1.561671	-0.157386
14	1	0	0.831088	2.368630	-0.046181
15	1	0	0.227523	1.141832	-1.157829
16	6	0	0.355120	0.512421	0.910422
17	1	0	-0.293989	-1.388384	1.767334
18	1	0	0.235792	0.912052	1.913923
19	6	0	1.455159	-0.518536	0.789582
20	1	0	2.030780	-0.713186	1.688801
21	6	0	2.274300	-0.620550	-0.464672
22	1	0	2.763684	-1.599078	-0.511194
23	1	0	1.661614	-0.506494	-1.361151
24	8	0	3.269421	0.411332	-0.429071
25	1	0	3.830763	0.310841	-1.205551

BCN_3

```
Zero-point correction=                           0.220397 (Hartree/Particle)
Thermal correction to Energy=                    0.230888
Thermal correction to Enthalpy=                  0.231832
Thermal correction to Gibbs Free Energy=         0.184533
Sum of electronic and zero-point Energies=           -464.317775
Sum of electronic and thermal Energies=              -464.307284
Sum of electronic and thermal Enthalpies=            -464.306340
Sum of electronic and thermal Free Energies=         -464.353639
```

No imaginary frequencies

Standard orientation:

Center Number	Atomic Number	Atomic Type	Coordinates (Angstroms) X	Y	Z
1	6	0	-2.318171	0.581165	-0.291287
2	6	0	-2.313754	-0.622843	-0.218393
3	6	0	-1.701181	1.911824	-0.280628
4	1	0	-1.969715	2.499697	-1.160200
5	1	0	-2.009584	2.477488	0.602671
6	6	0	-1.675411	-1.936098	-0.073552
7	1	0	-1.969440	-2.628076	-0.864837
8	1	0	-1.935719	-2.395746	0.883610
9	6	0	-0.154908	-1.640941	-0.149937
10	1	0	0.395377	-2.585471	-0.083467
11	1	0	0.044241	-1.209153	-1.130830
12	6	0	0.293185	-0.714620	0.970449
13	6	0	-0.173626	1.644588	-0.248967
14	1	0	0.349462	2.605937	-0.209197
15	1	0	0.113453	1.156441	-1.182575
16	6	0	0.236298	0.804191	0.946157
17	1	0	0.030898	-1.127744	1.941547

```
    18          1           0        -0.088347    1.243697    1.886131
    19          6           0         1.568515    0.100920    1.013405
    20          1           0         2.040251    0.125897    1.991301
    21          6           0         2.631025    0.198058   -0.067285
    22          1           0         2.941962    1.240681   -0.172768
    23          1           0         3.506743   -0.371997    0.258264
    24          8           0         2.256142   -0.225772   -1.375102
    25          1           0         2.149455   -1.182949   -1.358616
 ---------------------------------------------------------------------
```

BCN_4

```
 Zero-point correction=                           0.219981 (Hartree/Particle)
 Thermal correction to Energy=                    0.230629
 Thermal correction to Enthalpy=                  0.231573
 Thermal correction to Gibbs Free Energy=         0.184125
 Sum of electronic and zero-point Energies=           -464.317020
 Sum of electronic and thermal Energies=              -464.306371
 Sum of electronic and thermal Enthalpies=            -464.305427
 Sum of electronic and thermal Free Energies=         -464.352876
```

No imaginary frequencies

```
                         Standard orientation:
 ---------------------------------------------------------------------
 Center     Atomic      Atomic             Coordinates (Angstroms)
 Number     Number       Type             X           Y           Z
 ---------------------------------------------------------------------
      1          6           0         2.317615   -0.603110   -0.263334
      2          6           0         2.317665    0.603143   -0.262479
      3          6           0         1.693478   -1.928113   -0.179994
      4          1           0         1.974393   -2.570958   -1.016322
      5          1           0         1.983565   -2.438781    0.742256
      6          6           0         1.693438    1.928145   -0.179900
      7          1           0         1.974523    2.570465   -1.016580
      8          1           0         1.983285    2.439431    0.742080
      9          6           0         0.168035    1.649192   -0.197873
     10          1           0        -0.366066    2.603349   -0.137546
     11          1           0        -0.085130    1.193761   -1.154723
     12          6           0        -0.259839    0.762064    0.958106
     13          6           0         0.168051   -1.649275   -0.197751
     14          1           0        -0.365934   -2.603482   -0.137171
     15          1           0        -0.085347   -1.194051   -1.154637
     16          6           0        -0.259777   -0.762040    0.958163
     17          1           0         0.040093    1.184386    1.914413
     18          1           0         0.040196   -1.184274    1.914496
     19          6           0        -1.561113   -0.000031    1.023669
     20          1           0        -2.023629   -0.000015    2.006712
     21          6           0        -2.654103   -0.000115   -0.019385
     22          1           0        -3.282344   -0.884187    0.139524
     23          1           0        -3.282689    0.883668    0.139765
     24          8           0        -2.164352    0.000186   -1.359369
     25          1           0        -2.930808    0.000038   -1.942642
 ---------------------------------------------------------------------
```

Sydnone H

```
 Zero-point correction=                           0.132726 (Hartree/Particle)
 Thermal correction to Energy=                    0.141399
 Thermal correction to Enthalpy=                  0.142343
 Thermal correction to Gibbs Free Energy=         0.097850
 Sum of electronic and zero-point Energies=           -568.135588
 Sum of electronic and thermal Energies=              -568.126915
```

```
 Sum of electronic and thermal Enthalpies=           -568.125971
 Sum of electronic and thermal Free Energies=        -568.170464
```

No imaginary frequencies

Standard orientation:

Center Number	Atomic Number	Atomic Type	Coordinates (Angstroms) X	Y	Z
1	6	0	1.500287	0.833474	-0.377171
2	6	0	2.778239	0.300563	-0.142014
3	8	0	2.507651	-0.955730	0.416892
4	1	0	1.192586	1.759476	-0.827419
5	7	0	1.183063	-1.172268	0.527250
6	7	0	0.636011	-0.106420	0.045739
7	8	0	3.930447	0.662760	-0.313925
8	6	0	-0.802146	-0.021317	0.008923
9	6	0	-1.398928	1.198117	0.296478
10	6	0	-1.531557	-1.158818	-0.308560
11	6	0	-2.786046	1.274839	0.261483
12	1	0	-0.795745	2.059840	0.555240
13	6	0	-2.917733	-1.063601	-0.331533
14	1	0	-1.026571	-2.086934	-0.544192
15	6	0	-3.542873	0.148185	-0.049301
16	1	0	-3.273192	2.215648	0.485414
17	1	0	-3.507014	-1.937427	-0.580176
18	1	0	-4.623826	0.215318	-0.073360

TS $_{H/BCN1}$

```
 Zero-point correction=                           0.353887 (Hartree/Particle)
 Thermal correction to Energy=                    0.373669
 Thermal correction to Enthalpy=                  0.374613
 Thermal correction to Gibbs Free Energy=         0.304478
 Sum of electronic and zero-point Energies=          -1032.449395
 Sum of electronic and thermal Energies=             -1032.429613
 Sum of electronic and thermal Enthalpies=           -1032.428669
 Sum of electronic and thermal Free Energies=        -1032.498804
```

One imaginary frequency: -399.13i cm-1

Standard orientation:

Center Number	Atomic Number	Atomic Type	Coordinates (Angstroms) X	Y	Z
1	7	0	-2.402439	0.814544	-0.022164
2	7	0	-1.979067	1.184829	1.190684
3	6	0	-1.846394	1.603010	-0.962002
4	6	0	-1.544267	2.834201	-0.284319
5	8	0	-1.735450	2.543102	1.048013
6	8	0	-1.199187	3.936299	-0.653609
7	6	0	0.067591	0.637210	0.803952
8	6	0	0.225040	0.977272	-0.377637
9	6	0	0.580210	0.035199	2.044614
10	1	0	0.432290	0.718110	2.885838
11	1	0	0.014001	-0.874917	2.270763
12	6	0	1.100792	1.076713	-1.562880
13	1	0	1.133783	2.108028	-1.925989
14	1	0	0.688001	0.468435	-2.375039
15	6	0	2.514924	0.589263	-1.198244
16	1	0	3.151185	0.670596	-2.085367
17	1	0	2.930545	1.259021	-0.441995
18	6	0	2.512846	-0.847874	-0.715681

19	6	0	2.075047	-0.288401	1.868455
20	1	0	2.451735	-0.724440	2.799163
21	1	0	2.613651	0.648250	1.710137
22	6	0	2.311617	-1.259753	0.727912
23	1	0	2.065276	-1.528965	-1.434709
24	1	0	1.751398	-2.182987	0.848890
25	6	0	3.659873	-1.438382	0.070405
26	1	0	3.930943	-2.460065	-0.179824
27	6	0	4.844955	-0.599237	0.467553
28	1	0	4.551841	0.405878	0.766551
29	1	0	5.364858	-1.061947	1.313041
30	8	0	5.761482	-0.425576	-0.620261
31	1	0	6.065690	-1.298739	-0.892915
32	6	0	-2.742738	-0.561934	-0.181076
33	6	0	-3.598770	-1.145704	0.747235
34	6	0	-2.197330	-1.289006	-1.233959
35	6	0	-3.913172	-2.492107	0.613654
36	1	0	-4.014464	-0.549344	1.549977
37	6	0	-2.532762	-2.633209	-1.360770
38	1	0	-1.516741	-0.820782	-1.935196
39	6	0	-3.384909	-3.235414	-0.439780
40	1	0	-4.580672	-2.957658	1.328454
41	1	0	-2.116694	-3.210283	-2.177565
42	1	0	-3.638120	-4.283805	-0.542304
43	1	0	-2.018029	1.478326	-2.019283

H/BCN$_1$

```
Zero-point correction=                           0.357817 (Hartree/Particle)
Thermal correction to Energy=                    0.376861
Thermal correction to Enthalpy=                  0.377805
Thermal correction to Gibbs Free Energy=         0.309329
Sum of electronic and zero-point Energies=          -1032.514548
Sum of electronic and thermal Energies=             -1032.495504
Sum of electronic and thermal Enthalpies=           -1032.494560
Sum of electronic and thermal Free Energies=        -1032.563036
```

No imaginary frequencies

Standard orientation:

Center Number	Atomic Number	Atomic Type	Coordinates (Angstroms) X	Y	Z
1	7	0	2.446016	-0.697276	-0.019108
2	7	0	1.706842	-1.094176	1.162772
3	6	0	1.583031	-1.418120	-0.961385
4	6	0	1.733378	-2.786018	-0.321899
5	8	0	1.830365	-2.546984	1.021767
6	8	0	1.753300	-3.888813	-0.784615
7	6	0	0.269309	-0.821240	0.787734
8	6	0	0.159848	-1.000382	-0.531961
9	6	0	-0.541177	-0.470932	1.988702
10	1	0	-0.427632	-1.298707	2.699152
11	1	0	-0.046656	0.389354	2.457621
12	6	0	-0.882711	-0.959914	-1.604511
13	1	0	-0.889969	-1.949172	-2.077285
14	1	0	-0.502695	-0.274941	-2.373781
15	6	0	-2.305799	-0.557334	-1.232989
16	1	0	-2.901260	-0.574675	-2.150649
17	1	0	-2.740354	-1.306219	-0.569657
18	6	0	-2.359491	0.825788	-0.625511
19	6	0	-2.019385	-0.163089	1.784098
20	1	0	-2.449428	0.058699	2.765169
21	1	0	-2.528102	-1.054829	1.417112

22	6	0	-2.222486	1.019843	0.863684
23	1	0	-1.889876	1.586941	-1.241933
24	1	0	-1.671513	1.902936	1.173752
25	6	0	-3.542025	1.298572	0.184847
26	1	0	-3.790966	2.349584	0.072195
27	6	0	-4.749852	0.427491	0.404090
28	1	0	-4.477227	-0.604514	0.619082
29	1	0	-5.329793	0.802646	1.253705
30	8	0	-5.586554	0.369545	-0.757882
31	1	0	-5.880054	1.265636	-0.957719
32	6	0	2.532393	0.723604	-0.130255
33	6	0	2.947474	1.459261	0.981723
34	6	0	2.256616	1.370393	-1.333031
35	6	0	3.082622	2.838060	0.883553
36	1	0	3.162983	0.950328	1.913117
37	6	0	2.409913	2.752248	-1.422360
38	1	0	1.929853	0.810983	-2.201325
39	6	0	2.817481	3.492129	-0.318314
40	1	0	3.401879	3.402868	1.751642
41	1	0	2.198864	3.247542	-2.362887
42	1	0	2.928327	4.567248	-0.391217
43	1	0	1.841885	-1.383691	-2.013175

TS H/BCN1–CO2

```
Zero-point correction=                           0.356440 (Hartree/Particle)
Thermal correction to Energy=                    0.375178
Thermal correction to Enthalpy=                  0.376123
Thermal correction to Gibbs Free Energy=         0.308719
Sum of electronic and zero-point Energies=          -1032.505521
Sum of electronic and thermal Energies=             -1032.486783
Sum of electronic and thermal Enthalpies=           -1032.485838
Sum of electronic and thermal Free Energies=        -1032.553242
```

One imaginary frequency: -105.57i cm-1

Standard orientation:

Center Number	Atomic Number	Atomic Type	Coordinates (Angstroms) X	Y	Z
1	7	0	-1.970664	0.511232	0.083844
2	7	0	-1.243448	0.663588	1.244747
3	6	0	-1.141668	1.281563	-0.808733
4	6	0	-1.156852	2.579824	0.015387
5	8	0	-1.235730	2.203269	1.313701
6	8	0	-1.094980	3.725867	-0.337754
7	6	0	0.170808	0.391916	0.809917
8	6	0	0.265064	0.739308	-0.479666
9	6	0	1.000971	-0.093258	1.949517
10	1	0	0.887804	0.647599	2.750499
11	1	0	0.527909	-1.009023	2.323996
12	6	0	1.290171	0.822627	-1.566563
13	1	0	1.281269	1.858138	-1.927003
14	1	0	0.904022	0.223381	-2.400895
15	6	0	2.724709	0.399750	-1.266268
16	1	0	3.301674	0.529146	-2.186918
17	1	0	3.162097	1.077641	-0.532286
18	6	0	2.810605	-1.042270	-0.821715
19	6	0	2.480884	-0.348868	1.691254
20	1	0	2.927362	-0.677255	2.634366
21	1	0	2.969745	0.589403	1.427981
22	6	0	2.693631	-1.411045	0.635993
23	1	0	2.349833	-1.736365	-1.518272
24	1	0	2.163046	-2.334897	0.845974

25	6	0	4.010407	-1.582894	-0.081765
26	1	0	4.277116	-2.608817	-0.317959
27	6	0	5.206267	-0.722981	0.229246
28	1	0	4.920729	0.281019	0.539425
29	1	0	5.784891	-1.171223	1.043651
30	8	0	6.049424	-0.547420	-0.915854
31	1	0	6.359587	-1.417502	-1.191616
32	6	0	-3.025558	-0.374531	-0.086211
33	6	0	-3.534477	-1.091086	1.003525
34	6	0	-3.585458	-0.545445	-1.357530
35	6	0	-4.600557	-1.961059	0.811921
36	1	0	-3.095653	-0.964567	1.984547
37	6	0	-4.652130	-1.420530	-1.525182
38	1	0	-3.191358	0.001980	-2.205128
39	6	0	-5.169899	-2.134150	-0.447561
40	1	0	-4.986937	-2.510891	1.662600
41	1	0	-5.078223	-1.543705	-2.514277
42	1	0	-5.999514	-2.816001	-0.587058
43	1	0	-1.437827	1.383245	-1.845900

P H/BCN1

```
Zero-point correction=                           0.343778 (Hartree/Particle)
Thermal correction to Energy=                    0.360851
Thermal correction to Enthalpy=                  0.361795
Thermal correction to Gibbs Free Energy=         0.297949
Sum of electronic and zero-point Energies=          -844.076033
Sum of electronic and thermal Energies=             -844.058960
Sum of electronic and thermal Enthalpies=           -844.058016
Sum of electronic and thermal Free Energies=        -844.121862
```

No imaginary frequencies

Standard orientation:

Center Number	Atomic Number	Atomic Type	Coordinates (Angstroms) X	Y	Z
1	7	0	1.803742	-0.064143	0.381886
2	7	0	1.149230	1.090930	0.635419
3	6	0	1.000321	-1.145637	0.579700
4	6	0	-0.073912	0.735974	1.006239
5	6	0	-0.226032	-0.677937	0.984678
6	6	0	-1.085492	1.775173	1.395313
7	1	0	-1.113291	1.867192	2.485847
8	1	0	-0.738011	2.735264	1.005212
9	6	0	-1.466271	-1.458739	1.289583
10	1	0	-1.945782	-1.014613	2.164756
11	1	0	-1.186846	-2.474554	1.576967
12	6	0	-2.489181	-1.539471	0.121391
13	1	0	-2.323196	-2.477471	-0.414152
14	1	0	-3.487536	-1.616768	0.556876
15	6	0	-2.434277	-0.426941	-0.918467
16	6	0	-2.507050	1.492026	0.875358
17	1	0	-3.096730	2.407894	0.977753
18	1	0	-2.990959	0.740531	1.500562
19	6	0	-2.477064	1.041262	-0.568195
20	1	0	-1.798672	-0.657519	-1.766347
21	1	0	-1.866130	1.678163	-1.202103
22	6	0	-3.652537	0.405604	-1.263886
23	1	0	-3.766865	0.657546	-2.314108
24	6	0	-4.966515	0.211555	-0.558687
25	1	0	-4.836402	0.055619	0.511632
26	1	0	-5.591624	1.101011	-0.690206
27	8	0	-5.669157	-0.942195	-1.035616

28	1	0	-5.818121	-0.827895	-1.980968
29	6	0	3.157505	-0.057625	-0.038051
30	6	0	3.677537	1.065355	-0.680132
31	6	0	3.960777	-1.173702	0.194260
32	6	0	5.006180	1.064590	-1.089397
33	1	0	3.043699	1.923485	-0.859137
34	6	0	5.283666	-1.165779	-0.233936
35	1	0	3.564127	-2.035120	0.717291
36	6	0	5.813857	-0.048973	-0.873856
37	1	0	5.407427	1.939209	-1.587805
38	1	0	5.904145	-2.035162	-0.051295
39	1	0	6.847061	-0.045478	-1.199190
40	1	0	1.347090	-2.151684	0.400705

CO_2

```
Zero-point correction=                           0.011731 (Hartree/Particle)
Thermal correction to Energy=                    0.014344
Thermal correction to Enthalpy=                  0.015289
Thermal correction to Gibbs Free Energy=        -0.008950
Sum of electronic and zero-point Energies=           -188.559701
Sum of electronic and thermal Energies=              -188.557088
Sum of electronic and thermal Enthalpies=            -188.556143
Sum of electronic and thermal Free Energies=         -188.580382
```

No imaginary frequencies

Standard orientation:

Center Number	Atomic Number	Atomic Type	Coordinates (Angstroms) X	Y	Z
1	6	0	0.000000	0.000000	0.000000
2	8	0	0.000000	0.000000	1.155081
3	8	0	0.000000	0.000000	-1.155081

TS $_{H/BCN2}$

```
Zero-point correction=                           0.353646 (Hartree/Particle)
Thermal correction to Energy=                    0.373541
Thermal correction to Enthalpy=                  0.374485
Thermal correction to Gibbs Free Energy=         0.303948
Sum of electronic and zero-point Energies=          -1032.449357
Sum of electronic and thermal Energies=             -1032.429463
Sum of electronic and thermal Enthalpies=           -1032.428519
Sum of electronic and thermal Free Energies=        -1032.499055
```

One imaginary frequency: -400.65i cm-1

Standard orientation:

Center Number	Atomic Number	Atomic Type	Coordinates (Angstroms) X	Y	Z
1	7	0	-2.413610	0.754028	0.143087
2	7	0	-1.879535	1.080691	1.324375
3	6	0	-1.985667	1.609539	-0.805280
4	6	0	-1.658476	2.817119	-0.097268
5	8	0	-1.699714	2.453024	1.230098
6	8	0	-1.392788	3.949320	-0.440258
7	6	0	0.134599	0.634925	0.701195

8	6	0	0.154132	1.040614	-0.469962
9	6	0	0.788402	-0.015568	1.847288
10	1	0	0.710101	0.615938	2.736670
11	1	0	0.269966	-0.952777	2.077592
12	6	0	0.901312	1.240884	-1.728200
13	1	0	0.850521	2.288708	-2.037991
14	1	0	0.440163	0.650791	-2.527635
15	6	0	2.364708	0.811270	-1.520655
16	1	0	2.916429	0.981203	-2.450873
17	1	0	2.806857	1.457755	-0.760023
18	6	0	2.471220	-0.652374	-1.135835
19	6	0	2.265496	-0.287775	1.507500
20	1	0	2.735757	-0.777123	2.366221
21	1	0	2.772728	0.669308	1.365487
22	6	0	2.415253	-1.171586	0.285309
23	1	0	1.989526	-1.303284	-1.860572
24	1	0	1.894922	-2.119934	0.388719
25	6	0	3.703772	-1.252237	-0.500714
26	1	0	3.982843	-2.240223	-0.852463
27	6	0	4.887034	-0.403671	-0.134834
28	1	0	5.582911	-0.351543	-0.978680
29	1	0	4.593573	0.615618	0.124286
30	8	0	5.540344	-1.014084	0.986925
31	1	0	6.334210	-0.504799	1.183743
32	6	0	-2.721650	-0.625565	-0.051833
33	6	0	-3.461105	-1.284441	0.925081
34	6	0	-2.259598	-1.281404	-1.188032
35	6	0	-3.742463	-2.634046	0.755290
36	1	0	-3.814558	-0.742439	1.793468
37	6	0	-2.561639	-2.629826	-1.349213
38	1	0	-1.668263	-0.755621	-1.928383
39	6	0	-3.297479	-3.306488	-0.380931
40	1	0	-4.319381	-3.157640	1.507788
41	1	0	-2.210379	-3.151657	-2.231032
42	1	0	-3.525006	-4.357688	-0.511132
43	1	0	-2.260738	1.530072	-1.844952

H/BCN$_2$

```
Zero-point correction=                           0.358073 (Hartree/Particle)
Thermal correction to Energy=                    0.377007
Thermal correction to Enthalpy=                  0.377951
Thermal correction to Gibbs Free Energy=         0.309736
Sum of electronic and zero-point Energies=          -1032.514060
Sum of electronic and thermal Energies=             -1032.495126
Sum of electronic and thermal Enthalpies=           -1032.494182
Sum of electronic and thermal Free Energies=        -1032.562397
```

No imaginary frequencies

Standard orientation:

Center Number	Atomic Number	Atomic Type	Coordinates (Angstroms) X	Y	Z
1	7	0	-2.455185	-0.626720	-0.171570
2	7	0	-1.594303	-1.011639	-1.272910
3	6	0	-1.740846	-1.418421	0.835467
4	6	0	-1.866359	-2.754055	0.126232
5	8	0	-1.789793	-2.461837	-1.208115
6	8	0	-1.985418	-3.871181	0.536785
7	6	0	-0.202603	-0.813970	-0.718046
8	6	0	-0.261205	-1.048106	0.596228
9	6	0	0.753885	-0.442480	-1.800115
10	1	0	0.713377	-1.243483	-2.548357

11	1	0	0.327389	0.440645	-2.292815
12	6	0	0.649257	-1.098565	1.782110
13	1	0	0.564199	-2.106693	2.204795
14	1	0	0.213200	-0.429785	2.535586
15	6	0	2.118225	-0.745924	1.580101
16	1	0	2.620388	-0.846382	2.546960
17	1	0	2.578082	-1.476486	0.914328
18	6	0	2.282856	0.668021	1.068890
19	6	0	2.203012	-0.159148	-1.420394
20	1	0	2.730202	0.124064	-2.336064
21	1	0	2.677630	-1.073611	-1.063024
22	6	0	2.313767	0.959771	-0.410176
23	1	0	1.768093	1.404032	1.679678
24	1	0	1.812596	1.872555	-0.718941
25	6	0	3.555425	1.162088	0.425378
26	1	0	3.808697	2.195953	0.636024
27	6	0	4.761212	0.281495	0.269252
28	1	0	5.405994	0.375761	1.149139
29	1	0	4.488538	-0.770510	0.162820
30	8	0	5.476159	0.713332	-0.896690
31	1	0	6.282859	0.190798	-0.962250
32	6	0	-2.497832	0.792177	-0.014359
33	6	0	-2.795515	1.577349	-1.130048
34	6	0	-2.293495	1.389663	1.227322
35	6	0	-2.885196	2.956777	-0.996933
36	1	0	-2.956177	1.105051	-2.091491
37	6	0	-2.401368	2.773382	1.350901
38	1	0	-2.055247	0.791527	2.098741
39	6	0	-2.691673	3.562194	0.243705
40	1	0	-3.113651	3.560507	-1.867458
41	1	0	-2.246855	3.231141	2.320955
42	1	0	-2.767067	4.638193	0.344034
43	1	0	-2.122695	-1.410758	1.849780

TS H/BCN$_2$−CO2

```
Zero-point correction=                           0.356755 (Hartree/Particle)
Thermal correction to Energy=                    0.375360
Thermal correction to Enthalpy=                  0.376304
Thermal correction to Gibbs Free Energy=         0.309723
Sum of electronic and zero-point Energies=          -1032.504997
Sum of electronic and thermal Energies=             -1032.486392
Sum of electronic and thermal Enthalpies=           -1032.485448
Sum of electronic and thermal Free Energies=        -1032.552029
```

One imaginary frequency: -104.35i cm-1

Standard orientation:

Center Number	Atomic Number	Atomic Type	Coordinates (Angstroms) X	Y	Z
1	7	0	1.967779	0.485721	-0.173826
2	7	0	1.147363	0.535443	-1.280686
3	6	0	1.231774	1.367259	0.696912
4	6	0	1.201062	2.570975	-0.259770
5	8	0	1.162400	2.057605	-1.512572
6	8	0	1.190500	3.749336	-0.028648
7	6	0	-0.230411	0.349045	-0.704235
8	6	0	-0.207497	0.833178	0.543808
9	6	0	-1.157964	-0.237286	-1.714630
10	1	0	-1.104353	0.412911	-2.596551
11	1	0	-0.720483	-1.193362	-2.026661
12	6	0	-1.142401	1.066193	1.687802
13	1	0	-1.073821	2.132051	1.935639

14	1	0	-0.718757	0.539450	2.552229
15	6	0	-2.605890	0.674408	1.519542
16	1	0	-3.130084	0.947762	2.440249
17	1	0	-3.048622	1.270576	0.721334
18	6	0	-2.764525	-0.808937	1.271517
19	6	0	-2.616307	-0.450067	-1.322784
20	1	0	-3.121850	-0.895346	-2.184554
21	1	0	-3.095907	0.513885	-1.149033
22	6	0	-2.759509	-1.365957	-0.129432
23	1	0	-2.272948	-1.421375	2.021381
24	1	0	-2.259795	-2.321873	-0.253907
25	6	0	-4.024179	-1.408244	0.695071
26	1	0	-4.290410	-2.385969	1.083039
27	6	0	-5.218624	-0.567612	0.348035
28	1	0	-5.895681	-0.514179	1.206848
29	1	0	-4.937690	0.452232	0.077204
30	8	0	-5.892817	-1.189292	-0.754774
31	1	0	-6.695204	-0.687577	-0.935790
32	6	0	3.013977	-0.408659	0.004293
33	6	0	3.429395	-1.235559	-1.046353
34	6	0	3.661318	-0.474989	1.243367
35	6	0	4.490140	-2.110721	-0.848994
36	1	0	2.923562	-1.189260	-2.001829
37	6	0	4.721412	-1.356916	1.417200
38	1	0	3.339300	0.157965	2.061340
39	6	0	5.146274	-2.180205	0.377873
40	1	0	4.803968	-2.746308	-1.669233
41	1	0	5.216048	-1.398361	2.380849
42	1	0	5.971339	-2.866587	0.522060
43	1	0	1.616441	1.568586	1.689613

P $_{H/BCN2}$

```
Zero-point correction=                          0.343790 (Hartree/Particle)
Thermal correction to Energy=                   0.360929
Thermal correction to Enthalpy=                 0.361873
Thermal correction to Gibbs Free Energy=        0.297197
Sum of electronic and zero-point Energies=          -844.075185
Sum of electronic and thermal Energies=             -844.058046
Sum of electronic and thermal Enthalpies=           -844.057102
Sum of electronic and thermal Free Energies=        -844.121778
```

No imaginary frequencies

Standard orientation:

Center Number	Atomic Number	Atomic Type	Coordinates (Angstroms) X	Y	Z
1	7	0	-1.815480	0.133482	0.371127
2	7	0	-1.077062	-0.914239	0.799186
3	6	0	-1.082856	1.280894	0.343038
4	6	0	0.128804	-0.423140	1.053418
5	6	0	0.182960	0.970480	0.777224
6	6	0	1.218345	-1.310956	1.581024
7	1	0	1.273627	-1.211792	2.669955
8	1	0	0.930380	-2.344477	1.372247
9	6	0	1.377364	1.866767	0.877680
10	1	0	1.946405	1.593568	1.768548
11	1	0	1.044139	2.895686	1.030679
12	6	0	2.297827	1.835892	-0.369575
13	1	0	1.930145	2.586387	-1.073708
14	1	0	3.289800	2.175965	-0.064027
15	6	0	2.375808	0.521315	-1.144439
16	6	0	2.607497	-1.033304	0.977856

17	1	0	3.256395	-1.881011	1.218131
18	1	0	3.055775	-0.159252	1.451105
19	6	0	2.536178	-0.846275	-0.520352
20	1	0	1.746123	0.514866	-2.026939
21	1	0	1.986682	-1.636095	-1.025418
22	6	0	3.665643	-0.251366	-1.320591
23	1	0	3.830774	-0.678024	-2.304613
24	6	0	4.935899	0.182391	-0.649026
25	1	0	5.453798	0.925958	-1.264693
26	1	0	4.745162	0.627515	0.330102
27	8	0	5.766093	-0.976467	-0.494725
28	1	0	6.605079	-0.694184	-0.114935
29	6	0	-3.173133	-0.034555	0.000546
30	6	0	-3.633095	-1.291393	-0.390152
31	6	0	-4.039606	1.057968	0.026744
32	6	0	-4.964867	-1.448139	-0.757107
33	1	0	-2.950846	-2.130617	-0.409573
34	6	0	-5.365389	0.888970	-0.356879
35	1	0	-3.689753	2.028139	0.357141
36	6	0	-5.835517	-0.361558	-0.748186
37	1	0	-5.318655	-2.426464	-1.060601
38	1	0	-6.034842	1.740810	-0.336107
39	1	0	-6.870955	-0.488532	-1.040104
40	1	0	-1.501342	2.214649	0.001067

TS H/BCN$_1$/SB

```
Zero-point correction=                           0.353887 (Hartree/Particle)
Thermal correction to Energy=                    0.373669
Thermal correction to Enthalpy=                  0.374613
Thermal correction to Gibbs Free Energy=         0.304479
Sum of electronic and zero-point Energies=          -1032.449395
Sum of electronic and thermal Energies=             -1032.429613
Sum of electronic and thermal Enthalpies=           -1032.428669
Sum of electronic and thermal Free Energies=        -1032.498803
```

One imaginary frequency: -399.13i cm-1

Standard orientation:

Center Number	Atomic Number	Atomic Type	Coordinates (Angstroms) X	Y	Z
1	7	0	-2.402428	0.814593	-0.022162
2	7	0	-1.979048	1.184861	1.190688
3	6	0	-1.846336	1.603029	-0.961999
4	6	0	-1.544159	2.834210	-0.284317
5	8	0	-1.735361	2.543122	1.048014
6	8	0	-1.199027	3.936290	-0.653608
7	6	0	0.067616	0.637153	0.803949
8	6	0	0.225054	0.977207	-0.377644
9	6	0	0.580248	0.035157	2.044616
10	1	0	0.432346	0.718081	2.885832
11	1	0	0.014035	-0.874951	2.270785
12	6	0	1.100791	1.076625	-1.562899
13	1	0	1.133781	2.107935	-1.926022
14	1	0	0.687994	0.468335	-2.375045
15	6	0	2.514924	0.589191	-1.198254
16	1	0	3.151194	0.670518	-2.085373
17	1	0	2.930524	1.258966	-0.442011
18	6	0	2.512862	-0.847940	-0.715682
19	6	0	2.075085	-0.288457	1.868446
20	1	0	2.451787	-0.724471	2.799160
21	1	0	2.613681	0.648194	1.710103
22	6	0	2.311647	-1.259826	0.727913

23	1	0	2.065297	-1.529039	-1.434707
24	1	0	1.751423	-2.183055	0.848901
25	6	0	3.659899	-1.438430	0.070391
26	1	0	3.930997	-2.460100	-0.179867
27	6	0	4.844951	-0.599251	0.467551
28	1	0	5.364847	-1.061926	1.313063
29	1	0	4.551809	0.405870	0.766497
30	8	0	5.761496	-0.425576	-0.620254
31	1	0	6.065707	-1.298726	-0.892946
32	6	0	-2.742790	-0.561870	-0.181069
33	6	0	-3.598851	-1.145598	0.747241
34	6	0	-2.197407	-1.288970	-1.233945
35	6	0	-3.913318	-2.491986	0.613659
36	1	0	-4.014523	-0.549218	1.549978
37	6	0	-2.532902	-2.633158	-1.360755
38	1	0	-1.516787	-0.820775	-1.935173
39	6	0	-3.385085	-3.235320	-0.439770
40	1	0	-4.580844	-2.957503	1.328457
41	1	0	-2.116855	-3.210253	-2.177545
42	1	0	-3.638349	-4.283697	-0.542296
43	1	0	-2.017976	1.478353	-2.019281

TS H/BCN$_1$/ST

```
Zero-point correction=                           0.353836 (Hartree/Particle)
Thermal correction to Energy=                    0.373644
Thermal correction to Enthalpy=                  0.374588
Thermal correction to Gibbs Free Energy=         0.304497
Sum of electronic and zero-point Energies=          -1032.449435
Sum of electronic and thermal Energies=             -1032.429627
Sum of electronic and thermal Enthalpies=           -1032.428683
Sum of electronic and thermal Free Energies=        -1032.498774
```

One imaginary frequency: -400.67i cm-1

Standard orientation:

Center Number	Atomic Number	Atomic Type	Coordinates (Angstroms) X	Y	Z
1	7	0	-2.415976	0.749014	0.141435
2	7	0	-1.884160	1.077231	1.323251
3	6	0	-1.988944	1.605221	-0.806701
4	6	0	-1.665381	2.813771	-0.098621
5	8	0	-1.707264	2.449949	1.228773
6	8	0	-1.401928	3.946464	-0.441629
7	6	0	0.132115	0.636599	0.702693
8	6	0	0.151926	1.040529	-0.469035
9	6	0	0.787857	-0.007769	1.851111
10	1	0	0.704795	0.625375	2.738895
11	1	0	0.274760	-0.947523	2.082938
12	6	0	0.899256	1.240538	-1.727219
13	1	0	0.846083	2.287730	-2.038750
14	1	0	0.439658	0.648127	-2.525833
15	6	0	2.363762	0.814662	-1.519492
16	1	0	2.913818	0.982961	-2.450963
17	1	0	2.805653	1.464717	-0.761589
18	6	0	2.474755	-0.647293	-1.129557
19	6	0	2.266806	-0.271430	1.513073
20	1	0	2.740548	-0.753485	2.374034
21	1	0	2.767100	0.688605	1.367031
22	6	0	2.422763	-1.159692	0.294698
23	1	0	1.995198	-1.303251	-1.851228
24	1	0	1.907427	-2.110413	0.402214
25	6	0	3.711238	-1.235705	-0.490015

26	1	0	3.996794	-2.224372	-0.837654
27	6	0	4.894558	-0.377777	-0.130121
28	1	0	4.600786	0.636110	0.136923
29	1	0	5.581013	-0.313606	-0.980992
30	8	0	5.594885	-0.887284	1.011581
31	1	0	5.875790	-1.786758	0.808919
32	6	0	-2.721146	-0.631164	-0.053660
33	6	0	-3.458915	-1.291936	0.923260
34	6	0	-2.258319	-1.285619	-1.190351
35	6	0	-3.737761	-2.641998	0.752920
36	1	0	-3.813096	-0.751060	1.792046
37	6	0	-2.557908	-2.634512	-1.352095
38	1	0	-1.668343	-0.758389	-1.930750
39	6	0	-3.292069	-3.313043	-0.383848
40	1	0	-4.313374	-3.167041	1.505409
41	1	0	-2.206109	-3.155199	-2.234376
42	1	0	-3.517758	-4.364580	-0.514522
43	1	0	-2.262902	1.525008	-1.846606

TS H/BCN$_1$/AT

```
Zero-point correction=                           0.353880 (Hartree/Particle)
Thermal correction to Energy=                    0.373735
Thermal correction to Enthalpy=                  0.374679
Thermal correction to Gibbs Free Energy=         0.304235
Sum of electronic and zero-point Energies=          -1032.449109
Sum of electronic and thermal Energies=             -1032.429254
Sum of electronic and thermal Enthalpies=           -1032.428309
Sum of electronic and thermal Free Energies=        -1032.498753
```

One imaginary frequency: -401.64i cm-1

Standard orientation:

Center Number	Atomic Number	Atomic Type	Coordinates (Angstroms) X	Y	Z
1	7	0	2.379670	0.853189	-0.177229
2	7	0	1.750693	1.039571	-1.341656
3	6	0	1.857833	1.666604	0.760983
4	6	0	1.309841	2.775692	0.027668
5	8	0	1.353573	2.368102	-1.286607
6	8	0	0.878785	3.863447	0.344962
7	6	0	-0.177531	0.752920	0.547565
8	6	0	-0.144984	0.318390	-0.613123
9	6	0	-0.930077	0.946956	1.803941
10	1	0	-0.353772	0.576112	2.656002
11	1	0	-1.092354	2.017320	1.972221
12	6	0	-0.780947	-0.373729	-1.744553
13	1	0	-0.146812	-1.192757	-2.094839
14	1	0	-0.887350	0.325129	-2.581576
15	6	0	-2.159009	-0.906236	-1.310277
16	1	0	-2.616257	-1.422859	-2.160041
17	1	0	-2.011951	-1.650300	-0.524098
18	6	0	-3.080356	0.204627	-0.847262
19	6	0	-2.277269	0.207348	1.710659
20	1	0	-2.813214	0.338472	2.656261
21	1	0	-2.074805	-0.860244	1.601546
22	6	0	-3.137202	0.724753	0.573736
23	1	0	-3.221053	0.972373	-1.603168
24	1	0	-3.316868	1.793922	0.645188
25	6	0	-4.298664	-0.058387	0.005993
26	1	0	-5.183487	0.521963	-0.239227
27	6	0	-4.624841	-1.445147	0.492731
28	1	0	-3.734870	-1.991236	0.801571

29	1	0	-5.298629	-1.389988	1.354276
30	8	0	-5.227157	-2.242239	-0.534630
31	1	0	-6.033605	-1.797542	-0.819251
32	6	0	2.927880	-0.443833	0.049908
33	6	0	3.692387	-1.027574	-0.955445
34	6	0	2.672606	-1.099439	1.249671
35	6	0	4.211006	-2.299525	-0.749246
36	1	0	3.881680	-0.486966	-1.874470
37	6	0	3.211199	-2.367041	1.445524
38	1	0	2.058050	-0.637366	2.013017
39	6	0	3.975470	-2.968282	0.450102
40	1	0	4.809321	-2.763508	-1.523827
41	1	0	3.021486	-2.886978	2.376633
42	1	0	4.387619	-3.957708	0.607793
43	1	0	2.188667	1.679778	1.786937

TS H/BCN$_1$/AB

```
Zero-point correction=                           0.353916 (Hartree/Particle)
Thermal correction to Energy=                    0.373740
Thermal correction to Enthalpy=                  0.374684
Thermal correction to Gibbs Free Energy=         0.304510
Sum of electronic and zero-point Energies=          -1032.449128
Sum of electronic and thermal Energies=             -1032.429303
Sum of electronic and thermal Enthalpies=           -1032.428359
Sum of electronic and thermal Free Energies=        -1032.498534
```

One imaginary frequency: -399.00i cm-1

Standard orientation:

Center Number	Atomic Number	Atomic Type	Coordinates (Angstroms) X	Y	Z
1	7	0	-2.358339	0.917820	-0.018092
2	7	0	-1.812123	1.214007	1.165026
3	6	0	-1.713927	1.578229	-0.999256
4	6	0	-1.154678	2.738765	-0.359952
5	8	0	-1.328437	2.502321	0.985338
6	8	0	-0.630219	3.752914	-0.767490
7	6	0	0.239922	0.598471	-0.504145
8	6	0	0.088635	0.313533	0.692917
9	6	0	1.104722	0.621474	-1.701552
10	1	0	0.598629	0.148649	-2.547757
11	1	0	1.299834	1.660450	-1.989559
12	6	0	0.599761	-0.254627	1.949809
13	1	0	-0.089481	-1.010850	2.335277
14	1	0	0.660088	0.535544	2.706346
15	6	0	1.989728	-0.867334	1.698779
16	1	0	2.355100	-1.303597	2.633873
17	1	0	1.881879	-1.684566	0.982472
18	6	0	2.982408	0.168555	1.207271
19	6	0	2.427921	-0.105586	-1.397550
20	1	0	3.051572	-0.080024	-2.296924
21	1	0	2.208061	-1.155547	-1.190880
22	6	0	3.184193	0.531437	-0.248992
23	1	0	3.072928	1.009868	1.888764
24	1	0	3.388558	1.585412	-0.416589
25	6	0	4.269092	-0.199877	0.506791
26	1	0	5.139685	0.390761	0.777114
27	6	0	4.607758	-1.632570	0.193813
28	1	0	5.126974	-2.087755	1.043842
29	1	0	3.719246	-2.228056	-0.009918
30	8	0	5.423562	-1.743304	-0.979145
31	1	0	6.232531	-1.242404	-0.824148

32	6	0	-2.972131	-0.364416	-0.132504
33	6	0	-3.856910	-0.767949	0.862647
34	6	0	-2.661821	-1.183505	-1.212667
35	6	0	-4.442794	-2.023703	0.768639
36	1	0	-4.085260	-0.102618	1.685943
37	6	0	-3.268170	-2.432597	-1.299016
38	1	0	-1.954271	-0.859568	-1.966532
39	6	0	-4.153573	-2.854629	-0.311827
40	1	0	-5.134910	-2.348228	1.536005
41	1	0	-3.036954	-3.078759	-2.137014
42	1	0	-4.618683	-3.830417	-0.383560
43	1	0	-1.962352	1.481447	-2.044138

TS H/BCN$_2$/SB

```
Zero-point correction=                           0.353810 (Hartree/Particle)
Thermal correction to Energy=                    0.373604
Thermal correction to Enthalpy=                  0.374548
Thermal correction to Gibbs Free Energy=         0.304482
Sum of electronic and zero-point Energies=          -1032.449232
Sum of electronic and thermal Energies=             -1032.429438
Sum of electronic and thermal Enthalpies=           -1032.428493
Sum of electronic and thermal Free Energies=        -1032.498560
```

One imaginary frequency: -399.32i cm-1

Standard orientation:

Center Number	Atomic Number	Atomic Type	Coordinates (Angstroms) X	Y	Z
1	7	0	-2.397970	0.821928	-0.022589
2	7	0	-1.975244	1.190045	1.191180
3	6	0	-1.837588	1.608977	-0.961042
4	6	0	-1.532267	2.838714	-0.282212
5	8	0	-1.726672	2.547567	1.049641
6	8	0	-1.182704	3.939811	-0.650327
7	6	0	0.069836	0.635129	0.807383
8	6	0	0.230434	0.975371	-0.373735
9	6	0	0.578842	0.029865	2.047984
10	1	0	0.433166	0.712668	2.889683
11	1	0	0.008753	-0.878120	2.272926
12	6	0	1.106862	1.070096	-1.558802
13	1	0	1.147669	2.101666	-1.920400
14	1	0	0.689074	0.466311	-2.371766
15	6	0	2.517425	0.570818	-1.196346
16	1	0	3.151959	0.645283	-2.085248
17	1	0	2.941419	1.237176	-0.441578
18	6	0	2.503920	-0.865561	-0.712091
19	6	0	2.072540	-0.299142	1.872032
20	1	0	2.448285	-0.735688	2.802914
21	1	0	2.613876	0.635574	1.712525
22	6	0	2.304529	-1.272749	0.732471
23	1	0	2.050040	-1.544336	-1.429208
24	1	0	1.739956	-2.192932	0.855953
25	6	0	3.649212	-1.464371	0.072138
26	1	0	3.911306	-2.487390	-0.177721
27	6	0	4.837039	-0.631372	0.458203
28	1	0	4.542992	0.367705	0.786365
29	1	0	5.380039	-1.112223	1.278476
30	8	0	5.693649	-0.528262	-0.687679
31	1	0	6.490884	-0.056666	-0.423249
32	6	0	-2.742047	-0.553468	-0.182977
33	6	0	-3.602985	-1.134795	0.742301

34	6	0	-2.195185	-1.281978	-1.234117
35	6	0	-3.921012	-2.480219	0.607365
36	1	0	-4.019562	-0.537293	1.543741
37	6	0	-2.534233	-2.625135	-1.362345
38	1	0	-1.510735	-0.815671	-1.932886
39	6	0	-3.391367	-3.224915	-0.444390
40	1	0	-4.592362	-2.943902	1.319768
41	1	0	-2.117053	-3.203334	-2.177777
42	1	0	-3.647366	-4.272525	-0.547977
43	1	0	-2.007767	1.485353	-2.018682

TS H/BCN$_2$/ST

```
Zero-point correction=                           0.353647 (Hartree/Particle)
Thermal correction to Energy=                    0.373541
Thermal correction to Enthalpy=                  0.374485
Thermal correction to Gibbs Free Energy=         0.303948
Sum of electronic and zero-point Energies=          -1032.449357
Sum of electronic and thermal Energies=             -1032.429463
Sum of electronic and thermal Enthalpies=           -1032.428518
Sum of electronic and thermal Free Energies=        -1032.499056
```

One imaginary frequency: -400.64i cm-1

Standard orientation:

Center Number	Atomic Number	Atomic Type	Coordinates (Angstroms) X	Y	Z
1	7	0	-2.413656	0.753884	0.143062
2	7	0	-1.879660	1.080595	1.324372
3	6	0	-1.985792	1.609463	-0.805279
4	6	0	-1.658764	2.817070	-0.097240
5	8	0	-1.699993	2.452947	1.230120
6	8	0	-1.393206	3.949310	-0.440201
7	6	0	0.134543	0.635045	0.701240
8	6	0	0.154073	1.040784	-0.469900
9	6	0	0.788359	-0.015477	1.847312
10	1	0	0.710061	0.615999	2.736715
11	1	0	0.269930	-0.952696	2.077587
12	6	0	0.901271	1.241156	-1.728111
13	1	0	0.850448	2.288995	-2.037847
14	1	0	0.440160	0.651088	-2.527586
15	6	0	2.364673	0.811571	-1.520550
16	1	0	2.916433	0.981598	-2.450727
17	1	0	2.806772	1.457999	-0.759840
18	6	0	2.471180	-0.652102	-1.135846
19	6	0	2.265453	-0.287666	1.507501
20	1	0	2.735725	-0.777048	2.366197
21	1	0	2.772677	0.669427	1.365530
22	6	0	2.415195	-1.171415	0.285263
23	1	0	1.989466	-1.302948	-1.860629
24	1	0	1.894829	-2.119751	0.388615
25	6	0	3.703710	-1.252047	-0.500769
26	1	0	3.982735	-2.240018	-0.852597
27	6	0	4.887021	-0.403588	-0.134809
28	1	0	5.582892	-0.351411	-0.978656
29	1	0	4.593625	0.615690	0.124427
30	8	0	5.540300	-1.014183	0.986867
31	1	0	6.334125	-0.504896	1.183845
32	6	0	-2.721521	-0.625746	-0.051879
33	6	0	-3.460909	-1.284722	0.925020
34	6	0	-2.259364	-1.281521	-1.188073
35	6	0	-3.742080	-2.634365	0.755224
36	1	0	-3.814453	-0.742769	1.793400

37	6	0	-2.561220	-2.629985	-1.349258
38	1	0	-1.668094	-0.755658	-1.928418
39	6	0	-3.296983	-3.306747	-0.380987
40	1	0	-4.318940	-3.158036	1.507713
41	1	0	-2.209874	-3.151768	-2.231070
42	1	0	-3.524361	-4.357979	-0.511190
43	1	0	-2.260817	1.529973	-1.844960

TS H/BCN$_2$/AT

```
Zero-point correction=                           0.353796 (Hartree/Particle)
Thermal correction to Energy=                    0.373681
Thermal correction to Enthalpy=                  0.374625
Thermal correction to Gibbs Free Energy=         0.304127
Sum of electronic and zero-point Energies=          -1032.448887
Sum of electronic and thermal Energies=             -1032.429002
Sum of electronic and thermal Enthalpies=           -1032.428058
Sum of electronic and thermal Free Energies=        -1032.498556
```

One imaginary frequency: -401.85i cm-1

Standard orientation:

Center Number	Atomic Number	Atomic Type	Coordinates (Angstroms) X	Y	Z
1	7	0	2.379486	0.850346	-0.176594
2	7	0	1.751325	1.038365	-1.341267
3	6	0	1.858987	1.664734	0.761527
4	6	0	1.314025	2.775348	0.028151
5	8	0	1.357282	2.367895	-1.286082
6	8	0	0.885463	3.864022	0.345586
7	6	0	-0.177841	0.756047	0.547667
8	6	0	-0.146781	0.321843	-0.613217
9	6	0	-0.929855	0.950998	1.804243
10	1	0	-0.353623	0.579973	2.656273
11	1	0	-1.091600	2.021469	1.972322
12	6	0	-0.784625	-0.366869	-1.745720
13	1	0	-0.153154	-1.187678	-2.096661
14	1	0	-0.887819	0.333411	-2.581943
15	6	0	-2.165348	-0.895020	-1.314166
16	1	0	-2.624165	-1.405786	-2.166581
17	1	0	-2.022716	-1.643029	-0.530805
18	6	0	-3.082109	0.217850	-0.847404
19	6	0	-2.277016	0.211500	1.710595
20	1	0	-2.812719	0.340420	2.656667
21	1	0	-2.074009	-0.855577	1.598971
22	6	0	-3.137479	0.731804	0.575385
23	1	0	-3.220859	0.988430	-1.600650
24	1	0	-3.315763	1.800912	0.650332
25	6	0	-4.302274	-0.043683	0.005278
26	1	0	-5.184191	0.540021	-0.238066
27	6	0	-4.628628	-1.429024	0.483398
28	1	0	-3.736706	-1.969094	0.807431
29	1	0	-5.319934	-1.377704	1.331129
30	8	0	-5.250451	-2.135916	-0.598913
31	1	0	-5.500221	-3.010122	-0.280175
32	6	0	2.924259	-0.448025	0.050864
33	6	0	3.688551	-1.033479	-0.953640
34	6	0	2.665839	-1.103193	1.250199
35	6	0	4.203744	-2.306755	-0.747004
36	1	0	3.880395	-0.493130	-1.872294
37	6	0	3.201003	-2.372180	1.446507
38	1	0	2.051479	-0.639653	2.012818
39	6	0	3.965023	-2.975136	0.451928

40	1	0	4.801917	-2.772082	-1.520889
41	1	0	3.008842	-2.891815	2.377286
42	1	0	4.374517	-3.965614	0.609945
43	1	0	2.189450	1.677103	1.787623

TS H/BCN$_2$/AB

```
Zero-point correction=                           0.353940 (Hartree/Particle)
Thermal correction to Energy=                    0.373752
Thermal correction to Enthalpy=                  0.374697
Thermal correction to Gibbs Free Energy=         0.304649
Sum of electronic and zero-point Energies=          -1032.448811
Sum of electronic and thermal Energies=             -1032.428999
Sum of electronic and thermal Enthalpies=           -1032.428055
Sum of electronic and thermal Free Energies=        -1032.498102
```

One imaginary frequency: -399.16i cm-1

Standard orientation:

Center Number	Atomic Number	Atomic Type	Coordinates (Angstroms) X	Y	Z
1	7	0	2.358987	0.913036	0.023721
2	7	0	1.816832	1.218958	-1.158737
3	6	0	1.716138	1.571116	1.007456
4	6	0	1.164274	2.738259	0.373804
5	8	0	1.339428	2.508675	-0.972482
6	8	0	0.644397	3.752815	0.786142
7	6	0	-0.241454	0.602754	0.502856
8	6	0	-0.089570	0.325611	-0.695940
9	6	0	-1.107216	0.620419	1.699652
10	1	0	-0.602646	0.141960	2.543602
11	1	0	-1.300523	1.658120	1.993406
12	6	0	-0.601129	-0.232798	-1.957037
13	1	0	0.088119	-0.985798	-2.348730
14	1	0	-0.662283	0.563180	-2.707383
15	6	0	-1.990328	-0.848420	-1.708974
16	1	0	-2.355848	-1.280325	-2.646065
17	1	0	-1.880389	-1.668964	-0.997057
18	6	0	-2.984071	0.183868	-1.211878
19	6	0	-2.431901	-0.102085	1.391122
20	1	0	-3.056203	-0.078544	2.290036
21	1	0	-2.215080	-1.151803	1.179530
22	6	0	-3.185543	0.541967	0.245000
23	1	0	-3.074843	1.027958	-1.889804
24	1	0	-3.388640	1.595347	0.417225
25	6	0	-4.272666	-0.183668	-0.514944
26	1	0	-5.140716	0.410148	-0.782446
27	6	0	-4.610699	-1.613211	-0.205772
28	1	0	-5.156679	-2.056559	-1.045054
29	1	0	-3.715973	-2.213876	-0.030244
30	8	0	-5.438690	-1.630865	0.965846
31	1	0	-5.709058	-2.541653	1.125982
32	6	0	2.965895	-0.372936	0.132526
33	6	0	3.849463	-0.776694	-0.863597
34	6	0	2.650265	-1.195171	1.208747
35	6	0	4.428561	-2.035970	-0.774617
36	1	0	4.082118	-0.108939	-1.683710
37	6	0	3.249871	-2.447833	1.290118
38	1	0	1.943849	-0.870696	1.963433
39	6	0	4.133923	-2.870170	0.301847
40	1	0	5.119672	-2.360756	-1.542777
41	1	0	3.014537	-3.096433	2.125082
42	1	0	4.593722	-3.848757	0.369602

43	1	0	1.962183	1.467219	2.052233

TS-OMe/BCN$_1$/SB

```
Zero-point correction=                           0.386882 (Hartree/Particle)
Thermal correction to Energy=                    0.409143
Thermal correction to Enthalpy=                  0.410087
Thermal correction to Gibbs Free Energy=         0.334432
Sum of electronic and zero-point Energies=          -1146.931892
Sum of electronic and thermal Energies=             -1146.909631
Sum of electronic and thermal Enthalpies=           -1146.908686
Sum of electronic and thermal Free Energies=        -1146.984341
```

One imaginary frequency: -406.86i cm-1

Standard orientation:

Center Number	Atomic Number	Atomic Type	Coordinates (Angstroms) X	Y	Z
1	7	0	-1.458730	1.900869	-0.024935
2	7	0	-0.925495	2.113810	1.183595
3	6	0	-0.661377	2.430860	-0.973107
4	6	0	0.050219	3.490975	-0.311920
5	8	0	-0.227756	3.304523	1.023601
6	8	0	0.754820	4.400085	-0.695859
7	6	0	0.791015	0.887326	0.809615
8	6	0	1.055998	1.134757	-0.376652
9	6	0	1.058951	0.150837	2.055028
10	1	0	1.175843	0.848125	2.889367
11	1	0	0.200870	-0.487415	2.292909
12	6	0	1.903122	0.895805	-1.562982
13	1	0	2.308469	1.840465	-1.937055
14	1	0	1.292574	0.472974	-2.368512
15	6	0	3.043451	-0.071236	-1.196495
16	1	0	3.660267	-0.235538	-2.085863
17	1	0	3.679211	0.407315	-0.448275
18	6	0	2.518439	-1.403777	-0.699606
19	6	0	2.330622	-0.698453	1.878789
20	1	0	2.527867	-1.233434	2.813216
21	1	0	3.173120	-0.024770	1.709089
22	6	0	2.187133	-1.699483	0.748414
23	1	0	1.848057	-1.879324	-1.410776
24	1	0	1.327056	-2.350448	0.880370
25	6	0	3.371696	-2.367815	0.090803
26	1	0	3.245764	-3.419655	-0.149505
27	6	0	4.784529	-2.023072	0.480255
28	1	0	4.886259	-0.979198	0.772431
29	1	0	5.098982	-2.639942	1.328763
30	8	0	5.697148	-2.207750	-0.608952
31	1	0	5.650760	-3.131629	-0.880093
32	6	0	-2.250760	0.724224	-0.169117
33	6	0	-3.290918	0.499643	0.720390
34	6	0	-1.954768	-0.194063	-1.175688
35	6	0	-4.056790	-0.655906	0.612381
36	1	0	-3.510010	1.228000	1.491546
37	6	0	-2.724184	-1.337311	-1.293230
38	1	0	-1.128891	-0.021068	-1.855654
39	6	0	-3.774525	-1.574163	-0.400600
40	1	0	-4.867123	-0.818968	1.309351
41	1	0	-2.518809	-2.065776	-2.068136
42	1	0	-0.868826	2.359201	-2.028992
43	8	0	-4.469819	-2.726133	-0.594949
44	6	0	-5.554699	-3.001568	0.288147
45	1	0	-5.967720	-3.953417	-0.036991

46	1	0	-5.203193	-3.085217	1.318987
47	1	0	-6.319005	-2.224310	0.215932

TS-OMe/BCN$_1$/ST

```
Zero-point correction=                           0.386626 (Hartree/Particle)
Thermal correction to Energy=                    0.408928
Thermal correction to Enthalpy=                  0.409873
Thermal correction to Gibbs Free Energy=         0.333882
Sum of electronic and zero-point Energies=          -1146.932127
Sum of electronic and thermal Energies=             -1146.909825
Sum of electronic and thermal Enthalpies=           -1146.908881
Sum of electronic and thermal Free Energies=        -1146.984872
```

One imaginary frequency: -406.86i cm-1

Standard orientation:

Center Number	Atomic Number	Atomic Type	Coordinates (Angstroms) X	Y	Z
1	7	0	-1.469814	1.894473	0.135552
2	7	0	-0.852307	2.014849	1.316518
3	6	0	-0.754107	2.522836	-0.817508
4	6	0	-0.012809	3.537601	-0.119063
5	8	0	-0.188589	3.230733	1.211492
6	8	0	0.646784	4.492107	-0.471784
7	6	0	0.851107	0.863910	0.715013
8	6	0	1.024587	1.220121	-0.460373
9	6	0	1.214912	0.023619	1.866901
10	1	0	1.385288	0.645359	2.750322
11	1	0	0.380265	-0.644514	2.106350
12	6	0	1.791040	1.104947	-1.717834
13	1	0	2.148563	2.088231	-2.037093
14	1	0	1.135744	0.732674	-2.513068
15	6	0	2.974587	0.143811	-1.508249
16	1	0	3.542906	0.078552	-2.441714
17	1	0	3.639556	0.572791	-0.755778
18	6	0	2.509131	-1.243392	-1.107891
19	6	0	2.476443	-0.792185	1.529788
20	1	0	2.733123	-1.410948	2.395497
21	1	0	3.306803	-0.099954	1.372802
22	6	0	2.270971	-1.683985	0.321330
23	1	0	1.808493	-1.666340	-1.822954
24	1	0	1.428575	-2.360291	0.439700
25	6	0	3.424801	-2.259416	-0.465608
26	1	0	3.304525	-3.284403	-0.804395
27	6	0	4.850018	-1.919613	-0.120317
28	1	0	5.499086	-2.111833	-0.981198
29	1	0	4.964358	-0.872515	0.155407
30	8	0	5.318634	-2.668021	1.008119
31	1	0	5.249935	-3.604377	0.790093
32	6	0	-2.250679	0.716461	-0.052675
33	6	0	-3.222415	0.397966	0.884242
34	6	0	-2.010859	-0.110529	-1.149466
35	6	0	-3.975055	-0.761500	0.734428
36	1	0	-3.399242	1.056917	1.725539
37	6	0	-2.768132	-1.257221	-1.307356
38	1	0	-1.237031	0.133694	-1.867861
39	6	0	-3.749364	-1.588526	-0.367351
40	1	0	-4.732234	-0.998026	1.468976
41	1	0	-2.604899	-1.916609	-2.151099
42	1	0	-1.036462	2.536617	-1.858203
43	8	0	-4.437569	-2.736162	-0.606721
44	6	0	-5.451976	-3.105843	0.324491

45	1	0	-5.870647	-4.038005	-0.047081
46	1	0	-5.026476	-3.262989	1.318265
47	1	0	-6.232676	-2.342922	0.369147

TS-OMe/BCN$_1$/AT

```
Zero-point correction=                           0.386581 (Hartree/Particle)
Thermal correction to Energy=                    0.408969
Thermal correction to Enthalpy=                  0.409913
Thermal correction to Gibbs Free Energy=         0.334005
Sum of electronic and zero-point Energies=          -1146.931879
Sum of electronic and thermal Energies=             -1146.909491
Sum of electronic and thermal Enthalpies=           -1146.908547
Sum of electronic and thermal Free Energies=        -1146.984455
```

One imaginary frequency: -405.83i cm-1

Standard orientation:

Center Number	Atomic Number	Atomic Type	Coordinates (Angstroms) X	Y	Z
1	7	0	1.477483	1.786573	-0.183690
2	7	0	0.806979	1.773837	-1.340646
3	6	0	0.736134	2.390302	0.765470
4	6	0	-0.133057	3.282461	0.045860
5	8	0	0.022694	2.918942	-1.272628
6	8	0	-0.875371	4.182465	0.376038
7	6	0	-0.917045	0.901261	0.555532
8	6	0	-0.762313	0.507666	-0.610687
9	6	0	-1.687444	0.846285	1.815077
10	1	0	-1.022953	0.646322	2.660283
11	1	0	-2.153769	1.819991	2.001851
12	6	0	-1.174304	-0.330129	-1.747660
13	1	0	-0.330752	-0.926170	-2.106676
14	1	0	-1.481617	0.315447	-2.577914
15	6	0	-2.337046	-1.244150	-1.319428
16	1	0	-2.626667	-1.862733	-2.174806
17	1	0	-1.978246	-1.921273	-0.540919
18	6	0	-3.539609	-0.452938	-0.844938
19	6	0	-2.761777	-0.251538	1.711539
20	1	0	-3.308498	-0.295405	2.659047
21	1	0	-2.259430	-1.213050	1.585756
22	6	0	-3.739658	0.010450	0.582589
23	1	0	-3.898427	0.250259	-1.591498
24	1	0	-4.220430	0.981002	0.668728
25	6	0	-4.626674	-1.067810	0.004463
26	1	0	-5.642859	-0.766222	-0.232815
27	6	0	-4.534274	-2.495147	0.473414
28	1	0	-3.519840	-2.765651	0.761812
29	1	0	-5.181353	-2.645960	1.343983
30	8	0	-4.899712	-3.419020	-0.559293
31	1	0	-5.805446	-3.222972	-0.824692
32	6	0	2.397686	0.720299	0.030679
33	6	0	3.314862	0.413298	-0.963951
34	6	0	2.350754	-0.013104	1.215250
35	6	0	4.201591	-0.642638	-0.786584
36	1	0	3.343958	1.000699	-1.873377
37	6	0	3.242294	-1.054478	1.399438
38	1	0	1.621431	0.219746	1.981944
39	6	0	4.166291	-1.376710	0.400372
40	1	0	4.911819	-0.870953	-1.569109
41	1	0	3.228072	-1.640398	2.310373
42	1	0	1.057186	2.499090	1.789099
43	8	0	4.993465	-2.421190	0.671239

44	6	0	5.937877	-2.794458	-0.329518
45	1	0	6.483240	-3.642274	0.078019
46	1	0	5.428831	-3.091035	-1.249650
47	1	0	6.630161	-1.974445	-0.533446

TS-OMe/BCN$_1$/AB

```
Zero-point correction=                           0.386645 (Hartree/Particle)
Thermal correction to Energy=                    0.409047
Thermal correction to Enthalpy=                  0.409991
Thermal correction to Gibbs Free Energy=         0.333993
Sum of electronic and zero-point Energies=          -1146.931833
Sum of electronic and thermal Energies=             -1146.909430
Sum of electronic and thermal Enthalpies=           -1146.908486
Sum of electronic and thermal Free Energies=        -1146.984485
```

One imaginary frequency: -404.24i cm-1

Standard orientation:

Center Number	Atomic Number	Atomic Type	Coordinates (Angstroms) X	Y	Z
1	7	0	1.470825	1.768475	0.018225
2	7	0	0.869005	1.889402	-1.169611
3	6	0	0.660934	2.234123	0.988759
4	6	0	-0.188162	3.193550	0.334820
5	8	0	0.052876	3.002363	-1.006932
6	8	0	-0.971845	4.030078	0.729788
7	6	0	-0.941188	0.758731	0.498248
8	6	0	-0.711019	0.515523	-0.696196
9	6	0	-1.789658	0.554615	1.690238
10	1	0	-1.181203	0.240860	2.543000
11	1	0	-2.259061	1.504582	1.969335
12	6	0	-1.045759	-0.178743	-1.949406
13	1	0	-0.177170	-0.726004	-2.325831
14	1	0	-1.309903	0.561106	-2.713148
15	6	0	-2.222457	-1.139630	-1.699981
16	1	0	-2.450912	-1.664269	-2.633181
17	1	0	-1.903115	-1.892941	-0.977037
18	6	0	-3.460024	-0.404466	-1.221976
19	6	0	-2.866986	-0.502859	1.386239
20	1	0	-3.479623	-0.639992	2.283013
21	1	0	-2.371941	-1.456450	1.187958
22	6	0	-3.759727	-0.098286	0.230122
23	1	0	-3.767279	0.377406	-1.910962
24	1	0	-4.238086	0.864412	0.388657
25	6	0	-4.607518	-1.096603	-0.523517
26	1	0	-5.601254	-0.758020	-0.802031
27	6	0	-4.558190	-2.565772	-0.198384
28	1	0	-4.959818	-3.146228	-1.035610
29	1	0	-3.541949	-2.909987	-0.013454
30	8	0	-5.290850	-2.876591	0.993560
31	1	0	-6.206654	-2.607335	0.859447
32	6	0	2.409574	0.705638	0.153033
33	6	0	3.380359	0.539334	-0.824151
34	6	0	2.332115	-0.160290	1.242713
35	6	0	4.291770	-0.505507	-0.724372
36	1	0	3.432291	1.227514	-1.658808
37	6	0	3.247976	-1.191317	1.351226
38	1	0	1.561239	-0.036766	1.994082
39	6	0	4.227408	-1.370729	0.369291
40	1	0	5.043768	-0.623819	-1.492007
41	1	0	3.211724	-1.877371	2.188721
42	1	0	0.918650	2.218735	2.035604

43	8	0	5.076885	-2.414747	0.561125
44	6	0	6.087753	-2.633517	-0.420281
45	1	0	6.643775	-3.505715	-0.084892
46	1	0	5.641278	-2.834091	-1.396932
47	1	0	6.756699	-1.772347	-0.485100

TS-OMe/BCN$_2$/SB

```
Zero-point correction=                           0.386540 (Hartree/Particle)
Thermal correction to Energy=                    0.408927
Thermal correction to Enthalpy=                  0.409871
Thermal correction to Gibbs Free Energy=         0.333691
Sum of electronic and zero-point Energies=          -1146.931930
Sum of electronic and thermal Energies=             -1146.909543
Sum of electronic and thermal Enthalpies=           -1146.908599
Sum of electronic and thermal Free Energies=        -1146.984780
```

One imaginary frequency: -407.05i cm-1

Standard orientation:

Center Number	Atomic Number	Atomic Type	Coordinates (Angstroms) X	Y	Z
1	7	0	-1.453291	1.906020	-0.024441
2	7	0	-0.920758	2.116436	1.184983
3	6	0	-0.653723	2.435556	-0.971024
4	6	0	0.059542	3.493191	-0.307837
5	8	0	-0.220500	3.305867	1.027188
6	8	0	0.766616	4.401249	-0.689811
7	6	0	0.791391	0.885975	0.812168
8	6	0	1.060612	1.134833	-0.372886
9	6	0	1.052375	0.143700	2.055638
10	1	0	1.171504	0.837262	2.892743
11	1	0	0.190220	-0.490610	2.289341
12	6	0	1.907954	0.893983	-1.558685
13	1	0	2.321471	1.836882	-1.928240
14	1	0	1.295064	0.479712	-2.366892
15	6	0	3.039888	-0.084439	-1.195917
16	1	0	3.653483	-0.252274	-2.086799
17	1	0	3.681745	0.385244	-0.447125
18	6	0	2.503098	-1.413171	-0.702034
19	6	0	2.319884	-0.711313	1.877987
20	1	0	2.514901	-1.249572	2.811031
21	1	0	3.164885	-0.040650	1.709776
22	6	0	2.171082	-1.708979	0.745208
23	1	0	1.828980	-1.881848	-1.414104
24	1	0	1.306622	-2.354256	0.875959
25	6	0	3.348397	-2.387625	0.085917
26	1	0	3.213066	-3.436507	-0.157572
27	6	0	4.759636	-2.053199	0.474101
28	1	0	4.858544	-1.014535	0.795804
29	1	0	5.080101	-2.695729	1.300885
30	8	0	5.600391	-2.284632	-0.664641
31	1	0	6.514308	-2.146659	-0.392732
32	6	0	-2.246226	0.730144	-0.170365
33	6	0	-3.287253	0.505715	0.718160
34	6	0	-1.949505	-0.188140	-1.176730
35	6	0	-4.053166	-0.649747	0.609490
36	1	0	-3.506830	1.234005	1.489243
37	6	0	-2.718989	-1.331273	-1.294982
38	1	0	-1.122842	-0.015408	-1.855817
39	6	0	-3.770030	-1.568096	-0.403159
40	1	0	-4.864063	-0.812766	1.305816
41	1	0	-2.512941	-2.059811	-2.069640

42	1	0	-0.859765	2.365188	-2.027268
43	8	0	-4.465046	-2.720168	-0.597865
44	6	0	-5.549566	-2.996358	0.285429
45	1	0	-5.961915	-3.948546	-0.039568
46	1	0	-5.197793	-3.079677	1.316210
47	1	0	-6.314492	-2.219703	0.213316

TS-OMe/BCN$_2$/ST

```
Zero-point correction=                           0.386383 (Hartree/Particle)
Thermal correction to Energy=                    0.408803
Thermal correction to Enthalpy=                  0.409747
Thermal correction to Gibbs Free Energy=         0.333055
Sum of electronic and zero-point Energies=          -1146.932086
Sum of electronic and thermal Energies=             -1146.909666
Sum of electronic and thermal Enthalpies=           -1146.908722
Sum of electronic and thermal Free Energies=        -1146.985413
```

One imaginary frequency: -407.30i cm-1

Standard orientation:

Center Number	Atomic Number	Atomic Type	Coordinates (Angstroms) X	Y	Z
1	7	0	-1.464164	1.908034	0.134567
2	7	0	-0.850613	2.028236	1.318084
3	6	0	-0.748069	2.542555	-0.814153
4	6	0	-0.013428	3.558405	-0.110767
5	8	0	-0.192662	3.247289	1.218597
6	8	0	0.642944	4.517070	-0.458501
7	6	0	0.853087	0.881836	0.720576
8	6	0	1.036915	1.247740	-0.450366
9	6	0	1.194459	0.018841	1.862619
10	1	0	1.375332	0.624194	2.755251
11	1	0	0.343452	-0.632953	2.089607
12	6	0	1.812458	1.139231	-1.702855
13	1	0	2.186865	2.121615	-2.005039
14	1	0	1.159198	0.787561	-2.509044
15	6	0	2.979568	0.157547	-1.495784
16	1	0	3.557547	0.097860	-2.423679
17	1	0	3.641921	0.564024	-0.728771
18	6	0	2.485387	-1.227557	-1.123655
19	6	0	2.437673	-0.822283	1.519779
20	1	0	2.671922	-1.462460	2.376158
21	1	0	3.286732	-0.149081	1.380841
22	6	0	2.220119	-1.687268	0.294098
23	1	0	1.784149	-1.623699	-1.853318
24	1	0	1.360657	-2.344920	0.392696
25	6	0	3.368713	-2.277332	-0.491757
26	1	0	3.226677	-3.291936	-0.849632
27	6	0	4.791902	-1.979114	-0.118341
28	1	0	5.453736	-2.202144	-0.961743
29	1	0	4.929178	-0.929955	0.151145
30	8	0	5.139792	-2.811550	0.996687
31	1	0	6.068590	-2.661517	1.203787
32	6	0	-2.234665	0.723671	-0.057546
33	6	0	-3.207498	0.397733	0.875660
34	6	0	-1.981401	-0.103519	-1.151149
35	6	0	-3.947327	-0.769932	0.725861
36	1	0	-3.394531	1.056895	1.714593
37	6	0	-2.725947	-1.258559	-1.309136
38	1	0	-1.206635	0.146706	-1.866509
39	6	0	-3.707494	-1.597842	-0.372262
40	1	0	-4.705203	-1.012436	1.457758

41	1	0	-2.551889	-1.918671	-2.150150
42	1	0	-1.025510	2.556237	-1.856169
43	8	0	-4.381735	-2.753883	-0.610755
44	6	0	-5.393449	-3.133475	0.319358
45	1	0	-5.798972	-4.072397	-0.049777
46	1	0	-4.968295	-3.281552	1.314682
47	1	0	-6.184219	-2.380738	0.359438

TS-OMe/BCN$_2$/AT

```
Zero-point correction=                           0.386495 (Hartree/Particle)
Thermal correction to Energy=                    0.408896
Thermal correction to Enthalpy=                  0.409840
Thermal correction to Gibbs Free Energy=         0.333866
Sum of electronic and zero-point Energies=          -1146.931663
Sum of electronic and thermal Energies=             -1146.909262
Sum of electronic and thermal Enthalpies=           -1146.908317
Sum of electronic and thermal Free Energies=        -1146.984292
```

One imaginary frequency: -406.29i cm-1

Standard orientation:

Center Number	Atomic Number	Atomic Type	Coordinates (Angstroms) X	Y	Z
1	7	0	1.474378	1.785849	-0.183770
2	7	0	0.803780	1.771910	-1.340602
3	6	0	0.732371	2.389136	0.765214
4	6	0	-0.137366	3.280514	0.045125
5	8	0	0.018510	2.916411	-1.273178
6	8	0	-0.880250	4.180140	0.374953
7	6	0	-0.919224	0.899840	0.556648
8	6	0	-0.765133	0.505177	-0.609358
9	6	0	-1.689844	0.845690	1.816163
10	1	0	-1.025534	0.645572	2.661475
11	1	0	-2.155812	1.819618	2.002656
12	6	0	-1.178527	-0.331940	-1.746390
13	1	0	-0.336408	-0.930730	-2.104261
14	1	0	-1.482513	0.314520	-2.577193
15	6	0	-2.345292	-1.242158	-1.320664
16	1	0	-2.637416	-1.856701	-2.178046
17	1	0	-1.990415	-1.923311	-0.543739
18	6	0	-3.544378	-0.447005	-0.844532
19	6	0	-2.764063	-0.252102	1.712091
20	1	0	-3.310483	-0.297634	2.659723
21	1	0	-2.261170	-1.212924	1.584544
22	6	0	-3.742499	0.011994	0.584086
23	1	0	-3.901058	0.258928	-1.589399
24	1	0	-4.221610	0.983088	0.672649
25	6	0	-4.634920	-1.059886	0.003529
26	1	0	-5.648736	-0.753700	-0.233407
27	6	0	-4.544828	-2.485592	0.464968
28	1	0	-3.531469	-2.749360	0.774155
29	1	0	-5.212388	-2.644776	1.318273
30	8	0	-4.949653	-3.328965	-0.622606
31	1	0	-4.959370	-4.239083	-0.306822
32	6	0	2.396110	0.720983	0.030797
33	6	0	3.314262	0.415801	-0.963494
34	6	0	2.350067	-0.012631	1.215270
35	6	0	4.202960	-0.638426	-0.785854
36	1	0	3.342645	1.003410	-1.872809
37	6	0	3.243547	-1.052308	1.399736
38	1	0	1.620064	0.218882	1.981723
39	6	0	4.168625	-1.372621	0.401052

40	1	0	4.914002	-0.865240	-1.568075
41	1	0	3.230141	-1.638286	2.310648
42	1	0	1.053498	2.498838	1.788733
43	8	0	4.997834	-2.415425	0.672218
44	6	0	5.943812	-2.786328	-0.327949
45	1	0	6.490897	-3.632917	0.079831
46	1	0	5.436103	-3.083962	-1.248483
47	1	0	6.634300	-1.964653	-0.531285

TS-OMe/BCN$_2$/AB

```
Zero-point correction=                           0.386569 (Hartree/Particle)
Thermal correction to Energy=                    0.408985
Thermal correction to Enthalpy=                  0.409929
Thermal correction to Gibbs Free Energy=         0.333933
Sum of electronic and zero-point Energies=          -1146.931612
Sum of electronic and thermal Energies=             -1146.909196
Sum of electronic and thermal Enthalpies=           -1146.908252
Sum of electronic and thermal Free Energies=        -1146.984248
```

One imaginary frequency: -404.03i cm-1

Standard orientation:

Center Number	Atomic Number	Atomic Type	Coordinates (Angstroms) X	Y	Z
1	7	0	1.469385	1.770828	0.022572
2	7	0	0.868870	1.894463	-1.165704
3	6	0	0.659000	2.235771	0.993023
4	6	0	-0.188480	3.197095	0.339869
5	8	0	0.053886	3.008018	-1.002044
6	8	0	-0.971636	4.033847	0.735407
7	6	0	-0.945121	0.763083	0.496965
8	6	0	-0.711640	0.521130	-0.697120
9	6	0	-1.797347	0.559533	1.686363
10	1	0	-1.191700	0.246018	2.541210
11	1	0	-2.267016	1.509933	1.963535
12	6	0	-1.042149	-0.173945	-1.951019
13	1	0	-0.170984	-0.717843	-2.326359
14	1	0	-1.308147	0.564999	-2.714986
15	6	0	-2.215389	-1.139092	-1.702247
16	1	0	-2.439826	-1.667211	-2.634485
17	1	0	-1.894717	-1.888838	-0.976367
18	6	0	-3.456787	-0.407057	-1.229532
19	6	0	-2.874810	-0.497426	1.380546
20	1	0	-3.491390	-0.630754	2.275153
21	1	0	-2.380553	-1.452456	1.186944
22	6	0	-3.762389	-0.095539	0.219742
23	1	0	-3.763787	0.371818	-1.921936
24	1	0	-4.242376	0.866977	0.373715
25	6	0	-4.607391	-1.096767	-0.535153
26	1	0	-5.599317	-0.759704	-0.818273
27	6	0	-4.556727	-2.559760	-0.201726
28	1	0	-4.970039	-3.144854	-1.029879
29	1	0	-3.535273	-2.901833	-0.023700
30	8	0	-5.346853	-2.773710	0.976481
31	1	0	-5.362150	-3.719678	1.158377
32	6	0	2.406660	0.706510	0.155990
33	6	0	3.377409	0.540576	-0.821312
34	6	0	2.327636	-0.161396	1.243959
35	6	0	4.287094	-0.505930	-0.723469
36	1	0	3.430585	1.230296	-1.654616
37	6	0	3.241861	-1.194088	1.350619

38	1	0	1.556820	-0.038072	1.995425
39	6	0	4.221129	-1.373207	0.368466
40	1	0	5.038995	-0.623957	-1.491246
41	1	0	3.204406	-1.881688	2.186794
42	1	0	0.915395	2.218013	2.040157
43	8	0	5.068840	-2.419029	0.558321
44	6	0	6.079528	-2.637416	-0.423359
45	1	0	6.634099	-3.511193	-0.089680
46	1	0	5.632906	-2.835281	-1.400490
47	1	0	6.749864	-1.777191	-0.486318

TS-Me/BCN$_1$/SB

```
Zero-point correction=                           0.358249 (Hartree/Particle)
Thermal correction to Energy=                    0.381796
Thermal correction to Enthalpy=                  0.382740
Thermal correction to Gibbs Free Energy=         0.301887
Sum of electronic and zero-point Energies=          -1369.494795
Sum of electronic and thermal Energies=             -1369.471249
Sum of electronic and thermal Enthalpies=           -1369.470305
Sum of electronic and thermal Free Energies=        -1369.551158
```

One imaginary frequency: -392.81i cm-1

Standard orientation:

Center Number	Atomic Number	Atomic Type	Coordinates (Angstroms) X	Y	Z
1	7	0	-0.589584	2.395931	-0.028282
2	7	0	0.009779	2.554304	1.156483
3	6	0	0.260316	2.715197	-1.023091
4	6	0	1.200730	3.626238	-0.431498
5	8	0	0.936390	3.554153	0.919295
6	8	0	2.065618	4.350361	-0.874683
7	6	0	1.424887	0.952682	0.798203
8	6	0	1.696760	1.091067	-0.402038
9	6	0	1.541857	0.216720	2.066306
10	1	0	1.859971	0.887734	2.868868
11	1	0	0.560017	-0.177142	2.350755
12	6	0	2.417760	0.622482	-1.602610
13	1	0	3.030967	1.427215	-2.018047
14	1	0	1.695883	0.341333	-2.377143
15	6	0	3.294382	-0.586812	-1.228080
16	1	0	3.821817	-0.925770	-2.125451
17	1	0	4.052649	-0.260192	-0.512864
18	6	0	2.470461	-1.730623	-0.669743
19	6	0	2.552692	-0.931081	1.887660
20	1	0	2.638633	-1.472975	2.834839
21	1	0	3.532429	-0.498637	1.674338
22	6	0	2.125746	-1.894085	0.796172
23	1	0	1.678709	-2.043082	-1.345653
24	1	0	1.135681	-2.305804	0.972964
25	6	0	3.084066	-2.854494	0.131639
26	1	0	2.694144	-3.848586	-0.067470
27	6	0	4.550613	-2.858032	0.470903
28	1	0	4.913830	-1.862444	0.720911
29	1	0	4.733540	-3.507571	1.333490
30	8	0	5.351833	-3.293923	-0.633998
31	1	0	5.067387	-4.183476	-0.872587
32	6	0	-1.602338	1.395765	-0.088672
33	6	0	-2.594777	1.396870	0.887975
34	6	0	-1.555416	0.433662	-1.089476
35	6	0	-3.562545	0.407286	0.856957
36	1	0	-2.608965	2.166086	1.649512

37	6	0	-2.537766	-0.549747	-1.120867
38	1	0	-0.762795	0.436099	-1.827716
39	6	0	-3.528565	-0.557628	-0.148744
40	1	0	-4.345837	0.390658	1.606314
41	1	0	-2.515656	-1.306796	-1.894115
42	1	0	0.007042	2.639977	-2.068396
43	6	0	-4.592210	-1.613925	-0.148992
44	9	0	-4.562485	-2.354812	0.974716
45	9	0	-4.481211	-2.464036	-1.177015
46	9	0	-5.827577	-1.083624	-0.212910

TS-Me/BCN$_1$/ST

```
Zero-point correction=                           0.358576 (Hartree/Particle)
 Thermal correction to Energy=                    0.382055
 Thermal correction to Enthalpy=                  0.382999
 Thermal correction to Gibbs Free Energy=         0.302875
 Sum of electronic and zero-point Energies=          -1369.494536
 Sum of electronic and thermal Energies=             -1369.471057
 Sum of electronic and thermal Enthalpies=           -1369.470113
 Sum of electronic and thermal Free Energies=        -1369.550237
```

One imaginary frequency: -391.53i cm-1

Standard orientation:

Center Number	Atomic Number	Atomic Type	Coordinates (Angstroms) X	Y	Z
1	7	0	-0.614220	2.367498	0.149545
2	7	0	0.059862	2.407807	1.303065
3	6	0	0.168486	2.793958	-0.859971
4	6	0	1.144546	3.646468	-0.239054
5	8	0	0.968564	3.433597	1.111246
6	8	0	1.978675	4.418171	-0.660161
7	6	0	1.460726	0.865321	0.688289
8	6	0	1.647767	1.130872	-0.506682
9	6	0	1.685926	0.018592	1.869571
10	1	0	2.021285	0.626364	2.714434
11	1	0	0.741059	-0.447984	2.168726
12	6	0	2.310386	0.812486	-1.787505
13	1	0	2.849146	1.685984	-2.165652
14	1	0	1.555807	0.554609	-2.538440
15	6	0	3.277661	-0.365718	-1.572392
16	1	0	3.778425	-0.586198	-2.520601
17	1	0	4.048173	-0.052507	-0.865021
18	6	0	2.553813	-1.607636	-1.086953
19	6	0	2.733343	-1.057959	1.528346
20	1	0	2.890180	-1.683541	2.412671
21	1	0	3.682892	-0.562928	1.312485
22	6	0	2.294688	-1.931748	0.369787
23	1	0	1.748560	-1.902968	-1.754330
24	1	0	1.336375	-2.411828	0.549404
25	6	0	3.267055	-2.766697	-0.429675
26	1	0	2.920164	-3.756092	-0.713467
27	6	0	4.746977	-2.720839	-0.156131
28	1	0	5.297906	-3.092546	-1.026491
29	1	0	5.094142	-1.709444	0.048731
30	8	0	5.103348	-3.490205	0.999060
31	1	0	4.846590	-4.405310	0.838409
32	6	0	-1.633243	1.377625	0.051345
33	6	0	-2.544775	1.256715	1.097025
34	6	0	-1.673345	0.545182	-1.059616
35	6	0	-3.518243	0.275170	1.023113
36	1	0	-2.493756	1.927256	1.945143

37	6	0	-2.661479	-0.430261	-1.131993
38	1	0	-0.943365	0.641567	-1.853764
39	6	0	-3.571209	-0.560349	-0.091712
40	1	0	-4.239404	0.166023	1.825072
41	1	0	-2.705663	-1.086940	-1.991129
42	1	0	-0.151768	2.824509	-1.888984
43	6	0	-4.638557	-1.612078	-0.134202
44	9	0	-4.530399	-2.476468	0.892272
45	9	0	-4.609289	-2.338832	-1.257863
46	9	0	-5.871874	-1.080511	-0.045095

TS-Me/BCN$_1$/AT

```
Zero-point correction=                           0.358831 (Hartree/Particle)
Thermal correction to Energy=                    0.382208
Thermal correction to Enthalpy=                  0.383152
Thermal correction to Gibbs Free Energy=         0.303563
Sum of electronic and zero-point Energies=          -1369.494003
Sum of electronic and thermal Energies=             -1369.470626
Sum of electronic and thermal Enthalpies=           -1369.469682
Sum of electronic and thermal Free Energies=        -1369.549271
```

One imaginary frequency: -390.23i cm-1

Standard orientation:

Center Number	Atomic Number	Atomic Type	Coordinates (Angstroms) X	Y	Z
1	7	0	0.736438	2.171168	-0.193589
2	7	0	0.023065	2.144887	-1.322729
3	6	0	-0.025345	2.607483	0.827265
4	6	0	-1.051987	3.402429	0.209999
5	8	0	-0.915040	3.145785	-1.136861
6	8	0	-1.898449	4.159398	0.632845
7	6	0	-1.469780	0.891811	0.585525
8	6	0	-1.322533	0.595205	-0.607829
9	6	0	-2.156634	0.658762	1.872190
10	1	0	-1.428640	0.511138	2.674472
11	1	0	-2.747735	1.542181	2.137379
12	6	0	-1.662904	-0.218531	-1.784383
13	1	0	-0.761403	-0.659426	-2.218297
14	1	0	-2.103834	0.425132	-2.553425
15	6	0	-2.657311	-1.318976	-1.368866
16	1	0	-2.900460	-1.919452	-2.251105
17	1	0	-2.163214	-1.982274	-0.655309
18	6	0	-3.934297	-0.743511	-0.789353
19	6	0	-3.065597	-0.577695	1.742018
20	1	0	-3.552265	-0.755826	2.706351
21	1	0	-2.435325	-1.445060	1.534422
22	6	0	-4.125994	-0.398519	0.672655
23	1	0	-4.429476	-0.058045	-1.471681
24	1	0	-4.737207	0.483842	0.841098
25	6	0	-4.876260	-1.560519	0.062768
26	1	0	-5.935800	-1.397583	-0.112360
27	6	0	-4.556655	-2.984029	0.434483
28	1	0	-3.504289	-3.117313	0.679531
29	1	0	-5.143762	-3.281706	1.309705
30	8	0	-4.816591	-3.888899	-0.645867
31	1	0	-5.747944	-3.809542	-0.881381
32	6	0	1.810203	1.241495	-0.100316
33	6	0	2.663909	1.104832	-1.188140
34	6	0	1.964458	0.483150	1.057012
35	6	0	3.701761	0.185878	-1.117883
36	1	0	2.524372	1.715629	-2.070583

37	6	0	3.011690	-0.423062	1.124453
38	1	0	1.276435	0.587237	1.886992
39	6	0	3.869750	-0.568153	0.037352
40	1	0	4.376603	0.070806	-1.956110
41	1	0	3.151492	-1.021828	2.017212
42	1	0	0.329866	2.694238	1.841498
43	6	0	4.992366	-1.554925	0.150800
44	9	0	5.720533	-1.647640	-0.968726
45	9	0	4.546823	-2.793646	0.430073
46	9	0	5.844161	-1.232441	1.142695

TS-Me/BCN$_1$/AB

```
Zero-point correction=                           0.358641 (Hartree/Particle)
Thermal correction to Energy=                    0.382073
Thermal correction to Enthalpy=                  0.383017
Thermal correction to Gibbs Free Energy=         0.303560
Sum of electronic and zero-point Energies=          -1369.494166
Sum of electronic and thermal Energies=             -1369.470733
Sum of electronic and thermal Enthalpies=           -1369.469789
Sum of electronic and thermal Free Energies=        -1369.549247
```

One imaginary frequency: -390.84i cm-1

Standard orientation:

Center Number	Atomic Number	Atomic Type	Coordinates (Angstroms) X	Y	Z
1	7	0	-0.750964	2.162264	-0.021302
2	7	0	-0.103930	2.268468	1.142515
3	6	0	0.071800	2.474026	-1.040610
4	6	0	1.067151	3.335468	-0.462648
5	8	0	0.850965	3.239618	0.894835
6	8	0	1.942056	4.036936	-0.921736
7	6	0	1.486769	0.797044	-0.515743
8	6	0	1.269316	0.644991	0.693938
9	6	0	2.245389	0.419697	-1.725494
10	1	0	1.563696	0.171354	-2.543475
11	1	0	2.845881	1.272559	-2.060546
12	6	0	1.536873	-0.029233	1.973176
13	1	0	0.609070	-0.415539	2.403568
14	1	0	1.942408	0.695332	2.687739
15	6	0	2.540357	-1.173075	1.735552
16	1	0	2.723595	-1.681734	2.687342
17	1	0	2.079191	-1.901160	1.064908
18	6	0	3.856001	-0.664433	1.177906
19	6	0	3.154106	-0.781656	-1.404121
20	1	0	3.702552	-1.053831	-2.311634
21	1	0	2.522291	-1.633909	-1.143747
22	6	0	4.143160	-0.475584	-0.297176
23	1	0	4.310732	0.088634	1.815541
24	1	0	4.761828	0.390039	-0.517803
25	6	0	4.849453	-1.562543	0.478899
26	1	0	5.895047	-1.378616	0.708724
27	6	0	4.551915	-3.017537	0.232844
28	1	0	4.848173	-3.612055	1.103543
29	1	0	3.492093	-3.193401	0.055308
30	8	0	5.224780	-3.508788	-0.932747
31	1	0	6.172354	-3.396876	-0.795667
32	6	0	-1.827115	1.231679	-0.066125
33	6	0	-2.745862	1.232144	0.979988
34	6	0	-1.923910	0.341966	-1.129004
35	6	0	-3.786417	0.318407	0.957038

36	1	0	-2.650651	1.945096	1.788667
37	6	0	-2.977869	-0.563550	-1.150762
38	1	0	-1.189524	0.341483	-1.924856
39	6	0	-3.899074	-0.568946	-0.111274
40	1	0	-4.512689	0.305369	1.761087
41	1	0	-3.068045	-1.262448	-1.972571
42	1	0	-0.223834	2.439623	-2.076855
43	6	0	-5.014757	-1.570069	-0.098414
44	9	0	-4.757282	-2.597762	0.735010
45	9	0	-5.243959	-2.105328	-1.305013
46	9	0	-6.172762	-1.031350	0.317958

TS-Me/BCN$_2$/SB

```
Zero-point correction=                           0.358287 (Hartree/Particle)
Thermal correction to Energy=                    0.381775
Thermal correction to Enthalpy=                  0.382719
Thermal correction to Gibbs Free Energy=         0.302489
Sum of electronic and zero-point Energies=          -1369.494511
Sum of electronic and thermal Energies=             -1369.471022
Sum of electronic and thermal Enthalpies=           -1369.470078
Sum of electronic and thermal Free Energies=        -1369.550309
```

One imaginary frequency: -393.02i cm-1

Standard orientation:

Center Number	Atomic Number	Atomic Type	Coordinates (Angstroms) X	Y	Z
1	7	0	-0.586415	2.395500	-0.028501
2	7	0	0.012474	2.554349	1.156439
3	6	0	0.264496	2.712574	-1.023157
4	6	0	1.205724	3.623070	-0.432010
5	8	0	0.940541	3.552801	0.918667
6	8	0	2.071877	4.345522	-0.875474
7	6	0	1.426010	0.950745	0.800408
8	6	0	1.697668	1.086619	-0.400171
9	6	0	1.543361	0.216701	2.069606
10	1	0	1.863078	0.888645	2.870752
11	1	0	0.561499	-0.175840	2.355765
12	6	0	2.414730	0.613647	-1.601377
13	1	0	3.031278	1.415008	-2.018385
14	1	0	1.690098	0.335774	-2.374536
15	6	0	3.285787	-0.600211	-1.228407
16	1	0	3.806707	-0.943868	-2.127739
17	1	0	4.049448	-0.277505	-0.517034
18	6	0	2.457798	-1.738108	-0.664382
19	6	0	2.553223	-0.931937	1.890849
20	1	0	2.642288	-1.471760	2.838950
21	1	0	3.532075	-0.500080	1.672994
22	6	0	2.121233	-1.897111	0.803328
23	1	0	1.661559	-2.048153	-1.335983
24	1	0	1.131043	-2.306000	0.985592
25	6	0	3.069854	-2.864665	0.136287
26	1	0	2.673751	-3.855920	-0.059224
27	6	0	4.535325	-2.871545	0.461956
28	1	0	4.895772	-1.876639	0.731248
29	1	0	4.727217	-3.542164	1.306078
30	8	0	5.242046	-3.342560	-0.693795
31	1	0	6.175553	-3.411203	-0.465864
32	6	0	-1.600170	1.396292	-0.088797
33	6	0	-2.594339	1.400085	0.886059
34	6	0	-1.552479	0.432466	-1.087930

35	6	0	-3.563230	0.411580	0.854816
36	1	0	-2.609046	2.170561	1.646316
37	6	0	-2.535871	-0.549871	-1.119537
38	1	0	-0.758415	0.432712	-1.824634
39	6	0	-3.528533	-0.554968	-0.149289
40	1	0	-4.347974	0.397075	1.602692
41	1	0	-2.513116	-1.308257	-1.891454
42	1	0	0.011630	2.636691	-2.068518
43	6	0	-4.593398	-1.610059	-0.149949
44	9	0	-4.566290	-2.349726	0.974621
45	9	0	-4.481830	-2.461434	-1.176850
46	9	0	-5.828069	-1.078391	-0.216392

TS-Me/BCN$_2$/ST

```
Zero-point correction=                           0.358530 (Hartree/Particle)
Thermal correction to Energy=                    0.381987
Thermal correction to Enthalpy=                  0.382931
Thermal correction to Gibbs Free Energy=         0.302860
Sum of electronic and zero-point Energies=          -1369.494312
Sum of electronic and thermal Energies=             -1369.470856
Sum of electronic and thermal Enthalpies=           -1369.469911
Sum of electronic and thermal Free Energies=        -1369.549982
```

One imaginary frequency: -392.00i cm-1

Standard orientation:

Center Number	Atomic Number	Atomic Type	Coordinates (Angstroms) X	Y	Z
1	7	0	-0.590461	2.386051	0.149797
2	7	0	0.081985	2.421475	1.304787
3	6	0	0.196290	2.811055	-0.857270
4	6	0	1.174829	3.658089	-0.233109
5	8	0	0.995093	3.443664	1.116608
6	8	0	2.013031	4.427212	-0.651005
7	6	0	1.471011	0.871314	0.691268
8	6	0	1.667967	1.140512	-0.501373
9	6	0	1.669007	0.009794	1.866749
10	1	0	2.018469	0.601056	2.717569
11	1	0	0.709807	-0.431371	2.158987
12	6	0	2.332871	0.818313	-1.780081
13	1	0	2.894103	1.682602	-2.146485
14	1	0	1.578647	0.583844	-2.538992
15	6	0	3.271307	-0.383404	-1.566789
16	1	0	3.775966	-0.607592	-2.512069
17	1	0	4.041550	-0.094135	-0.849272
18	6	0	2.512493	-1.611313	-1.099774
19	6	0	2.685566	-1.094630	1.521321
20	1	0	2.815958	-1.733935	2.400054
21	1	0	3.651810	-0.627476	1.317372
22	6	0	2.229124	-1.942300	0.350365
23	1	0	1.705895	-1.878287	-1.777358
24	1	0	1.255585	-2.395399	0.517265
25	6	0	3.183507	-2.798603	-0.450036
26	1	0	2.809452	-3.773050	-0.747073
27	6	0	4.655628	-2.800052	-0.154574
28	1	0	5.206256	-3.206667	-1.009237
29	1	0	5.033122	-1.794655	0.042610
30	8	0	4.875074	-3.630034	0.994801
31	1	0	5.824687	-3.684141	1.147943
32	6	0	-1.610171	1.396927	0.049011
33	6	0	-2.524296	1.277331	1.092558

34	6	0	-1.646795	0.562361	-1.060467
35	6	0	-3.496417	0.294488	1.018441
36	1	0	-2.475839	1.949443	1.939589
37	6	0	-2.633545	-0.414519	-1.133079
38	1	0	-0.914838	0.657832	-1.852932
39	6	0	-3.545445	-0.543698	-0.094561
40	1	0	-4.219226	0.186092	1.819024
41	1	0	-2.674833	-1.073181	-1.990852
42	1	0	-0.121126	2.843532	-1.887113
43	6	0	-4.610322	-1.597976	-0.136402
44	9	0	-4.498764	-2.462605	0.889541
45	9	0	-4.580584	-2.324141	-1.260417
46	9	0	-5.844802	-1.069483	-0.045516

TS-Me/BCN$_2$/AT

```
Zero-point correction=                           0.358629 (Hartree/Particle)
Thermal correction to Energy=                    0.382078
Thermal correction to Enthalpy=                  0.383022
Thermal correction to Gibbs Free Energy=         0.303291
Sum of electronic and zero-point Energies=          -1369.493892
Sum of electronic and thermal Energies=             -1369.470442
Sum of electronic and thermal Enthalpies=           -1369.469498
Sum of electronic and thermal Free Energies=        -1369.549229
```

One imaginary frequency: -390.77i cm-1

Standard orientation:

Center Number	Atomic Number	Atomic Type	Coordinates (Angstroms) X	Y	Z
1	7	0	0.737535	2.163485	-0.194946
2	7	0	0.023212	2.136834	-1.323326
3	6	0	-0.023609	2.598482	0.826943
4	6	0	-1.051571	3.392888	0.210857
5	8	0	-0.915928	3.136729	-1.136082
6	8	0	-1.898337	4.148732	0.634973
7	6	0	-1.464918	0.881351	0.588209
8	6	0	-1.323763	0.586958	-0.606404
9	6	0	-2.147009	0.646707	1.877157
10	1	0	-1.416114	0.491321	2.675318
11	1	0	-2.731841	1.532274	2.149020
12	6	0	-1.675847	-0.218402	-1.785281
13	1	0	-0.780440	-0.666776	-2.224089
14	1	0	-2.112164	0.433569	-2.549971
15	6	0	-2.681034	-1.310315	-1.372663
16	1	0	-2.935126	-1.901389	-2.258148
17	1	0	-2.191547	-1.984457	-0.666061
18	6	0	-3.948569	-0.724713	-0.782944
19	6	0	-3.063555	-0.583822	1.744699
20	1	0	-3.546262	-0.765457	2.710392
21	1	0	-2.439827	-1.453494	1.527695
22	6	0	-4.128494	-0.390153	0.682523
23	1	0	-4.440161	-0.029481	-1.457788
24	1	0	-4.731119	0.496141	0.860698
25	6	0	-4.895814	-1.537899	0.068837
26	1	0	-5.953381	-1.361955	-0.099443
27	6	0	-4.588556	-2.964310	0.423056
28	1	0	-3.534488	-3.105588	0.670202
29	1	0	-5.183657	-3.269133	1.290204
30	8	0	-4.931843	-3.782381	-0.704119
31	1	0	-4.816414	-4.704575	-0.450575
32	6	0	1.813393	1.236259	-0.102345

33	6	0	2.666822	1.101096	-1.190553
34	6	0	1.970521	0.479321	1.055552
35	6	0	3.707529	0.185373	-1.119945
36	1	0	2.525025	1.710733	-2.073439
37	6	0	3.020540	-0.423584	1.123317
38	1	0	1.282689	0.582123	1.885861
39	6	0	3.878528	-0.566980	0.035945
40	1	0	4.382321	0.071625	-1.958393
41	1	0	3.162691	-1.020967	2.016630
42	1	0	0.332963	2.686218	1.840617
43	6	0	5.004363	-1.550004	0.149965
44	9	0	5.732404	-1.641548	-0.969740
45	9	0	4.562896	-2.789883	0.430666
46	9	0	5.855451	-1.223805	1.141234

TS-Me/BCN$_2$/AB

```
Zero-point correction=                           0.358342 (Hartree/Particle)
Thermal correction to Energy=                    0.381905
Thermal correction to Enthalpy=                  0.382849
Thermal correction to Gibbs Free Energy=         0.302362
Sum of electronic and zero-point Energies=          -1369.494139
Sum of electronic and thermal Energies=             -1369.470576
Sum of electronic and thermal Enthalpies=           -1369.469632
Sum of electronic and thermal Free Energies=        -1369.550119
```

One imaginary frequency: -391.41i cm-1

Standard orientation:

Center Number	Atomic Number	Atomic Type	Coordinates (Angstroms) X	Y	Z
1	7	0	0.754563	2.118995	0.024435
2	7	0	0.102548	2.236506	-1.135269
3	6	0	-0.063071	2.421560	1.050502
4	6	0	-1.059901	3.289954	0.485089
5	8	0	-0.850109	3.206776	-0.874144
6	8	0	-1.931902	3.987768	0.955018
7	6	0	-1.484591	0.755662	0.519624
8	6	0	-1.277123	0.613891	-0.693095
9	6	0	-2.244487	0.380488	1.729258
10	1	0	-1.564077	0.098553	2.537378
11	1	0	-2.816336	1.245409	2.083090
12	6	0	-1.567982	-0.038812	-1.978426
13	1	0	-0.652983	-0.444691	-2.418053
14	1	0	-1.957757	0.704351	-2.682602
15	6	0	-2.600594	-1.158187	-1.748043
16	1	0	-2.801840	-1.652018	-2.704012
17	1	0	-2.156171	-1.904918	-1.086932
18	6	0	-3.899202	-0.618698	-1.179504
19	6	0	-3.192565	-0.786950	1.396554
20	1	0	-3.746116	-1.052041	2.302955
21	1	0	-2.590468	-1.656426	1.122518
22	6	0	-4.174857	-0.434337	0.297646
23	1	0	-4.333419	0.154082	-1.807707
24	1	0	-4.763487	0.448747	0.530468
25	6	0	-4.920972	-1.489284	-0.487141
26	1	0	-5.959472	-1.267151	-0.710409
27	6	0	-4.671296	-2.950412	-0.249157
28	1	0	-5.029575	-3.533958	-1.103584
29	1	0	-3.608956	-3.164665	-0.114880
30	8	0	-5.392385	-3.335502	0.929455
31	1	0	-5.301892	-4.288421	1.037851

32	6	0	1.834506	1.192492	0.056249
33	6	0	2.752772	1.211663	-0.989909
34	6	0	1.935755	0.288725	1.106958
35	6	0	3.797881	0.302704	-0.980021
36	1	0	2.652608	1.933943	-1.789688
37	6	0	2.994664	-0.611009	1.116372
38	1	0	1.200567	0.272878	1.901956
39	6	0	3.912457	-0.601697	0.073643
40	1	0	4.523701	0.304605	-1.784433
41	1	0	3.088678	-1.320939	1.928343
42	1	0	0.237790	2.377727	2.084895
43	6	0	5.081060	-1.540451	0.093162
44	9	0	5.362522	-2.024243	-1.127696
45	9	0	4.884125	-2.594989	0.896235
46	9	0	6.204450	-0.934070	0.525627

TS-CO/BCN$_1$/SB

```
Zero-point correction=                           0.368802 (Hartree/Particle)
Thermal correction to Energy=                    0.391530
Thermal correction to Enthalpy=                  0.392475
Thermal correction to Gibbs Free Energy=         0.314649
Sum of electronic and zero-point Energies=          -1221.015994
Sum of electronic and thermal Energies=             -1220.993266
Sum of electronic and thermal Enthalpies=           -1220.992322
Sum of electronic and thermal Free Energies=        -1221.070147
```

One imaginary frequency: -387.87i cm-1

Standard orientation:

Center Number	Atomic Number	Atomic Type	Coordinates (Angstroms) X	Y	Z
1	7	0	-1.098828	2.131740	-0.032961
2	7	0	-0.515230	2.335904	1.151544
3	6	0	-0.283197	2.530793	-1.027602
4	6	0	0.575108	3.516901	-0.431106
5	8	0	0.321043	3.413645	0.919358
6	8	0	1.373302	4.315526	-0.871311
7	6	0	1.052360	0.873782	0.778447
8	6	0	1.300633	1.043793	-0.422591
9	6	0	1.286150	0.178611	2.053335
10	1	0	1.474766	0.902086	2.851190
11	1	0	0.388226	-0.381157	2.336301
12	6	0	2.084616	0.685367	-1.621637
13	1	0	2.545055	1.577318	-2.056164
14	1	0	1.418620	0.268267	-2.384625
15	6	0	3.163341	-0.342653	-1.233536
16	1	0	3.741255	-0.595693	-2.128130
17	1	0	3.852156	0.126338	-0.527246
18	6	0	2.560755	-1.607898	-0.654984
19	6	0	2.487772	-0.770988	1.891155
20	1	0	2.667903	-1.274963	2.846012
21	1	0	3.372376	-0.169644	1.671424
22	6	0	2.247502	-1.810582	0.813097
23	1	0	1.842239	-2.071390	-1.325806
24	1	0	1.349720	-2.394840	0.995575
25	6	0	3.368108	-2.588230	0.162876
26	1	0	3.167120	-3.639376	-0.022968
27	6	0	4.810051	-2.319467	0.502210
28	1	0	4.984093	-1.275065	0.755738
29	1	0	5.109904	-2.926535	1.362969
30	8	0	5.678037	-2.596246	-0.603743
31	1	0	5.559954	-3.521185	-0.848065

32	6	0	-2.039688	1.065829	-0.098764
33	6	0	-2.989378	0.955588	0.912052
34	6	0	-1.972448	0.153985	-1.147773
35	6	0	-3.894361	-0.093327	0.869690
36	1	0	-3.022168	1.686709	1.709549
37	6	0	-2.890112	-0.885005	-1.185421
38	1	0	-1.213568	0.243820	-1.915195
39	6	0	-3.847666	-1.012428	-0.180176
40	1	0	-4.640250	-0.188176	1.647751
41	1	0	-2.857231	-1.606701	-1.991929
42	1	0	-0.531335	2.442728	-2.073076
43	8	0	-4.808847	-2.968436	-1.152019
44	6	0	-4.806707	-2.146443	-0.262094
45	8	0	-5.678138	-2.177571	0.749371
46	1	0	-6.270630	-2.937162	0.630306

TS-CO/BCN$_1$/ST

```
Zero-point correction=                           0.368702 (Hartree/Particle)
Thermal correction to Energy=                    0.391438
Thermal correction to Enthalpy=                  0.392382
Thermal correction to Gibbs Free Energy=         0.314511
Sum of electronic and zero-point Energies=          -1221.016104
Sum of electronic and thermal Energies=             -1220.993369
Sum of electronic and thermal Enthalpies=           -1220.992424
Sum of electronic and thermal Free Energies=        -1221.070295
```

One imaginary frequency: -389.07i cm-1

Standard orientation:

Center Number	Atomic Number	Atomic Type	Coordinates (Angstroms) X	Y	Z
1	7	0	-1.092929	2.144108	0.134978
2	7	0	-0.433285	2.247364	1.292521
3	6	0	-0.349862	2.646703	-0.869483
4	6	0	0.536365	3.587406	-0.241344
5	8	0	0.374408	3.355461	1.107384
6	8	0	1.295070	4.436461	-0.656360
7	6	0	1.118558	0.850070	0.682652
8	6	0	1.284612	1.136083	-0.510568
9	6	0	1.430794	0.036565	1.867470
10	1	0	1.680112	0.680690	2.715176
11	1	0	0.546587	-0.541640	2.156623
12	6	0	1.993162	0.900071	-1.784550
13	1	0	2.423412	1.833011	-2.159769
14	1	0	1.283590	0.549636	-2.541744
15	6	0	3.096588	-0.149092	-1.556959
16	1	0	3.630427	-0.306947	-2.499607
17	1	0	3.815628	0.257211	-0.842509
18	6	0	2.526703	-1.470510	-1.077033
19	6	0	2.606166	-0.903544	1.539412
20	1	0	2.829428	-1.504940	2.426304
21	1	0	3.489704	-0.295229	1.333143
22	6	0	2.292389	-1.824502	0.376978
23	1	0	1.772774	-1.864276	-1.753407
24	1	0	1.399430	-2.420274	0.546073
25	6	0	3.370872	-2.531227	-0.409760
26	1	0	3.154657	-3.556374	-0.696468
27	6	0	4.829231	-2.300757	-0.117003
28	1	0	5.433505	-2.584842	-0.985081
29	1	0	5.041220	-1.256686	0.107941
30	8	0	5.265178	-3.038903	1.031022
31	1	0	5.101940	-3.973264	0.859456

32	6	0	-2.023278	1.071986	0.031194
33	6	0	-2.907622	0.856969	1.083440
34	6	0	-2.009470	0.257729	-1.097183
35	6	0	-3.801247	-0.199296	1.002269
36	1	0	-2.899825	1.514619	1.943098
37	6	0	-2.916600	-0.788545	-1.172705
38	1	0	-1.298731	0.427650	-1.896350
39	6	0	-3.809181	-1.020247	-0.127068
40	1	0	-4.497371	-0.374928	1.811681
41	1	0	-2.925323	-1.435674	-2.040814
42	1	0	-0.665511	2.650319	-1.900212
43	8	0	-4.826303	-2.879299	-1.223643
44	6	0	-4.761140	-2.156097	-0.254134
45	8	0	-5.549373	-2.308562	0.813039
46	1	0	-6.146237	-3.058220	0.658700

TS-CO/BCN$_1$/AT

```
Zero-point correction=                           0.368918 (Hartree/Particle)
Thermal correction to Energy=                    0.391586
Thermal correction to Enthalpy=                  0.392530
Thermal correction to Gibbs Free Energy=         0.315337
Sum of electronic and zero-point Energies=          -1221.015678
Sum of electronic and thermal Energies=             -1221.993010
Sum of electronic and thermal Enthalpies=           -1220.992066
Sum of electronic and thermal Free Energies=        -1221.069258
```

One imaginary frequency: -389.76i cm-1

Standard orientation:

Center Number	Atomic Number	Atomic Type	Coordinates (Angstroms) X	Y	Z
1	7	0	-1.149778	1.993673	0.173646
2	7	0	-0.454116	2.003542	1.313805
3	6	0	-0.402092	2.487104	-0.831824
4	6	0	0.562750	3.339490	-0.192242
5	8	0	0.422379	3.062274	1.150029
6	8	0	1.366379	4.151647	-0.595858
7	6	0	1.142990	0.862125	-0.584318
8	6	0	1.002888	0.547970	0.605208
9	6	0	1.859418	0.687257	-1.863965
10	1	0	1.153812	0.486744	-2.674668
11	1	0	2.383829	1.614356	-2.120164
12	6	0	1.389293	-0.243406	1.782774
13	1	0	0.519132	-0.754447	2.203377
14	1	0	1.769920	0.428409	2.559922
15	6	0	2.471018	-1.261802	1.375529
16	1	0	2.748421	-1.846736	2.258139
17	1	0	2.038739	-1.957159	0.652447
18	6	0	3.706963	-0.586113	0.815492
19	6	0	2.859590	-0.475530	-1.725994
20	1	0	3.370870	-0.611825	-2.684374
21	1	0	2.296745	-1.390425	-1.528598
22	6	0	3.889039	-0.218163	-0.642387
23	1	0	4.139202	0.131608	1.507362
24	1	0	4.430181	0.710994	-0.798593
25	6	0	4.720193	-1.321332	-0.029490
26	1	0	5.761860	-1.078118	0.159466
27	6	0	4.517923	-2.761670	-0.417285
28	1	0	3.477519	-2.980484	-0.652133
29	1	0	5.117241	-2.997795	-1.302996
30	8	0	4.867612	-3.654006	0.647811

31	1	0	5.795140	-3.506305	0.864740
32	6	0	-2.163843	1.002805	0.055289
33	6	0	-3.008754	0.783953	1.139160
34	6	0	-2.272539	0.268477	-1.121725
35	6	0	-3.985267	-0.194601	1.040845
36	1	0	-2.906936	1.379109	2.037288
37	6	0	-3.262069	-0.698662	-1.213216
38	1	0	-1.592402	0.437115	-1.947333
39	6	0	-4.115213	-0.934344	-0.136097
40	1	0	-4.650376	-0.372499	1.875500
41	1	0	-3.365375	-1.282627	-2.119045
42	1	0	-0.746088	2.561937	-1.850815
43	8	0	-5.323061	-2.638871	-1.288301
44	6	0	-5.154768	-1.988011	-0.280494
45	8	0	-5.896842	-2.155169	0.817105
46	1	0	-6.551025	-2.852662	0.650467

TS-CO/BCN$_1$/AB

```
Zero-point correction=                           0.368811 (Hartree/Particle)
Thermal correction to Energy=                    0.391517
Thermal correction to Enthalpy=                  0.392461
Thermal correction to Gibbs Free Energy=         0.314862
Sum of electronic and zero-point Energies=          -1221.015743
Sum of electronic and thermal Energies=             -1220.993037
Sum of electronic and thermal Enthalpies=           -1220.992093
Sum of electronic and thermal Free Energies=        -1221.069692
```

One imaginary frequency: -391.15i cm-1

Standard orientation:

Center Number	Atomic Number	Atomic Type	Coordinates (Angstroms) X	Y	Z
1	7	0	-1.156723	1.983749	-0.043789
2	7	0	-0.521596	2.141321	1.120646
3	6	0	-0.352461	2.337758	-1.064168
4	6	0	0.586107	3.264005	-0.491713
5	8	0	0.372295	3.167270	0.865966
6	8	0	1.417991	4.013393	-0.955097
7	6	0	1.161273	0.753768	-0.522054
8	6	0	0.955239	0.603444	0.689768
9	6	0	1.942806	0.410615	-1.727440
10	1	0	1.279264	0.097675	-2.538152
11	1	0	2.478817	1.298512	-2.080287
12	6	0	1.272662	-0.028091	1.979326
13	1	0	0.377128	-0.476885	2.417314
14	1	0	1.621747	0.737177	2.681184
15	6	0	2.360750	-1.095776	1.759858
16	1	0	2.582683	-1.572138	2.720064
17	1	0	1.956509	-1.868725	1.102897
18	6	0	3.633165	-0.497815	1.190851
19	6	0	2.937696	-0.714962	-1.386148
20	1	0	3.503217	-0.963565	-2.289878
21	1	0	2.370387	-1.606098	-1.107625
22	6	0	3.902712	-0.314597	-0.288140
23	1	0	4.029233	0.299504	1.813624
24	1	0	4.452881	0.591623	-0.526540
25	6	0	4.692098	-1.329266	0.505488
26	1	0	5.720527	-1.060053	0.728670
27	6	0	4.510323	-2.807278	0.286674
28	1	0	4.866607	-3.360209	1.162339
29	1	0	3.466507	-3.073508	0.128012
30	8	0	5.205865	-3.261759	-0.880732

31	1	0	6.137814	-3.039979	-0.773204
32	6	0	-2.172901	0.988787	-0.086052
33	6	0	-3.088697	0.929642	0.959914
34	6	0	-2.214450	0.094932	-1.151777
35	6	0	-4.071271	-0.047366	0.935713
36	1	0	-3.036205	1.645440	1.770088
37	6	0	-3.209605	-0.870627	-1.170961
38	1	0	-1.480148	0.141022	-1.946493
39	6	0	-4.135305	-0.945582	-0.131286
40	1	0	-4.791925	-0.101744	1.741017
41	1	0	-3.262168	-1.576452	-1.990354
42	1	0	-0.643481	2.278034	-2.100413
43	8	0	-5.284532	-2.798725	-1.100824
44	6	0	-5.181534	-2.000895	-0.195099
45	8	0	-6.010142	-1.989161	0.852092
46	1	0	-6.664600	-2.697447	0.741985

TS-CO/BCN$_2$/SB

```
Zero-point correction=                           0.368799 (Hartree/Particle)
Thermal correction to Energy=                    0.391503
Thermal correction to Enthalpy=                  0.392447
Thermal correction to Gibbs Free Energy=         0.314826
Sum of electronic and zero-point Energies=          -1221.015740
Sum of electronic and thermal Energies=             -1220.993037
Sum of electronic and thermal Enthalpies=           -1220.992093
Sum of electronic and thermal Free Energies=        -1221.069714
```

One imaginary frequency: -388.23i cm-1

Standard orientation:

Center Number	Atomic Number	Atomic Type	Coordinates (Angstroms) X	Y	Z
1	7	0	-1.092370	2.135622	-0.035260
2	7	0	-0.510226	2.343814	1.149191
3	6	0	-0.275561	2.531216	-1.030350
4	6	0	0.582149	3.519299	-0.436239
5	8	0	0.326703	3.420579	0.914162
6	8	0	1.381055	4.316209	-0.878266
7	6	0	1.058497	0.880226	0.782450
8	6	0	1.304639	1.042983	-0.419993
9	6	0	1.294571	0.192169	2.060805
10	1	0	1.490734	0.919894	2.852971
11	1	0	0.395280	-0.361248	2.351754
12	6	0	2.080363	0.671744	-1.620484
13	1	0	2.549228	1.556856	-2.059934
14	1	0	1.406740	0.259705	-2.379515
15	6	0	3.148669	-0.367600	-1.233688
16	1	0	3.716076	-0.633382	-2.131252
17	1	0	3.849050	0.096401	-0.535424
18	6	0	2.535039	-1.621480	-0.642230
19	6	0	2.490585	-0.764496	1.898167
20	1	0	2.674921	-1.262427	2.855409
21	1	0	3.376569	-0.169611	1.667332
22	6	0	2.235518	-1.810579	0.829951
23	1	0	1.805412	-2.081083	-1.303523
24	1	0	1.334750	-2.386157	1.024699
25	6	0	3.339614	-2.606381	0.174911
26	1	0	3.124723	-3.655267	-0.002051
27	6	0	4.785000	-2.348480	0.489161
28	1	0	4.964522	-1.305461	0.757527
29	1	0	5.100804	-2.973832	1.330671

30	8	0	5.555524	-2.685180	-0.673077
31	1	0	6.488284	-2.594889	-0.449824
32	6	0	-2.032869	1.069212	-0.099673
33	6	0	-2.984554	0.961171	0.909471
34	6	0	-1.963455	0.154836	-1.146406
35	6	0	-3.889505	-0.087855	0.867529
36	1	0	-3.019095	1.694118	1.705223
37	6	0	-2.881018	-0.884200	-1.183657
38	1	0	-1.203174	0.242769	-1.912644
39	6	0	-3.840735	-1.009275	-0.180199
40	1	0	-4.637057	-0.180944	1.644210
41	1	0	-2.846419	-1.607681	-1.988493
42	1	0	-0.522965	2.440323	-2.075755
43	8	0	-4.800144	-2.966977	-1.150320
44	6	0	-4.799909	-2.143188	-0.262057
45	8	0	-5.673890	-2.171952	0.747272
46	1	0	-6.266400	-2.931518	0.628116

TS-CO/BCN$_2$/ST

```
Zero-point correction=                           0.368601 (Hartree/Particle)
Thermal correction to Energy=                    0.391350
Thermal correction to Enthalpy=                  0.392294
Thermal correction to Gibbs Free Energy=         0.314567
Sum of electronic and zero-point Energies=          -1221.015936
Sum of electronic and thermal Energies=             -1220.993187
Sum of electronic and thermal Enthalpies=           -1220.992243
Sum of electronic and thermal Free Energies=        -1221.069970
```

One imaginary frequency: -389.12i cm-1

Standard orientation:

Center Number	Atomic Number	Atomic Type	Coordinates (Angstroms) X	Y	Z
1	7	0	-1.081888	2.151931	0.139034
2	7	0	-0.420339	2.247645	1.296370
3	6	0	-0.338147	2.656847	-0.863776
4	6	0	0.551987	3.591461	-0.232343
5	8	0	0.390466	3.353940	1.115620
6	8	0	1.313034	4.440095	-0.644068
7	6	0	1.122026	0.846371	0.679842
8	6	0	1.293418	1.139833	-0.510872
9	6	0	1.419074	0.017377	1.857710
10	1	0	1.674580	0.649320	2.712685
11	1	0	0.525309	-0.550508	2.138008
12	6	0	2.005657	0.908724	-1.783711
13	1	0	2.444316	1.841493	-2.149519
14	1	0	1.297013	0.569469	-2.546839
15	6	0	3.100524	-0.150119	-1.558925
16	1	0	3.640503	-0.302350	-2.499018
17	1	0	3.816355	0.244119	-0.834795
18	6	0	2.516476	-1.471848	-1.097418
19	6	0	2.581332	-0.937209	1.525247
20	1	0	2.788081	-1.553102	2.406059
21	1	0	3.476440	-0.341288	1.332537
22	6	0	2.262060	-1.838859	0.349297
23	1	0	1.765278	-1.850971	-1.785043
24	1	0	1.359666	-2.424090	0.503896
25	6	0	3.339036	-2.552358	-0.435455
26	1	0	3.111302	-3.570152	-0.735208
27	6	0	4.790978	-2.343498	-0.115712

28	1	0	5.409301	-2.669135	-0.958723
29	1	0	5.016198	-1.293678	0.082793
30	8	0	5.106459	-3.129776	1.041801
31	1	0	6.048328	-3.034633	1.220466
32	6	0	-2.013822	1.081371	0.032549
33	6	0	-2.897658	0.864952	1.084946
34	6	0	-2.000854	0.269028	-1.097165
35	6	0	-3.791388	-0.191104	1.002820
36	1	0	-2.889007	1.521129	1.945722
37	6	0	-2.908218	-0.777039	-1.173690
38	1	0	-1.290413	0.440070	-1.896386
39	6	0	-3.799970	-1.010412	-0.127701
40	1	0	-4.486862	-0.368042	1.812508
41	1	0	-2.917549	-1.422916	-2.042722
42	1	0	-0.654267	2.665423	-1.894322
43	8	0	-4.817709	-2.868213	-1.225893
44	6	0	-4.751620	-2.146463	-0.255378
45	8	0	-5.538408	-2.300903	0.812601
46	1	0	-6.135071	-3.050660	0.657984

TS-CO/BCN$_2$/AT

Zero-point correction= 0.368893 (Hartree/Particle)
Thermal correction to Energy= 0.391556
Thermal correction to Enthalpy= 0.392501
Thermal correction to Gibbs Free Energy= 0.315341
Sum of electronic and zero-point Energies= -1221.015406
Sum of electronic and thermal Energies= -1220.992743
Sum of electronic and thermal Enthalpies= -1220.991799
Sum of electronic and thermal Free Energies= -1221.068959

One imaginary frequency: -389.72i cm-1

Standard orientation:

Center Number	Atomic Number	Atomic Type	Coordinates (Angstroms) X	Y	Z
1	7	0	1.145700	1.992919	-0.176607
2	7	0	0.448822	2.000058	-1.315961
3	6	0	0.398632	2.487098	0.828957
4	6	0	-0.568072	3.337197	0.189002
5	8	0	-0.429054	3.057789	-1.152890
6	8	0	-1.372291	4.148924	0.592229
7	6	0	-1.143787	0.858795	0.586690
8	6	0	-1.006785	0.544275	-0.603076
9	6	0	-1.857004	0.683088	1.868063
10	1	0	-1.149327	0.482621	2.676968
11	1	0	-2.381488	1.609669	2.125961
12	6	0	-1.397879	-0.244567	-1.780740
13	1	0	-0.530474	-0.758889	-2.203032
14	1	0	-1.776410	0.429841	-2.556683
15	6	0	-2.483876	-1.258802	-1.374292
16	1	0	-2.766915	-1.838099	-2.258774
17	1	0	-2.053906	-1.959866	-0.655225
18	6	0	-3.714593	-0.579344	-0.807644
19	6	0	-2.856353	-0.480588	1.731138
20	1	0	-3.363871	-0.620413	2.691032
21	1	0	-2.293058	-1.393909	1.528626
22	6	0	-3.890206	-0.219973	0.652575
23	1	0	-4.146401	0.143068	-1.494728
24	1	0	-4.429837	0.708916	0.815163
25	6	0	-4.728916	-1.315351	0.037103
26	1	0	-5.769064	-1.066828	-0.147432

27	6	0	-4.527713	-2.756062	0.408488
28	1	0	-3.488148	-2.969361	0.665363
29	1	0	-5.149278	-3.008322	1.274024
30	8	0	-4.920144	-3.559866	-0.712866
31	1	0	-4.853592	-4.485693	-0.455095
32	6	0	2.161491	1.003928	-0.057559
33	6	0	3.004722	0.783196	-1.142361
34	6	0	2.273602	0.273222	1.121417
35	6	0	3.983208	-0.193286	-1.042835
36	1	0	2.900196	1.375376	-2.042142
37	6	0	3.265031	-0.691830	1.214036
38	1	0	1.594682	0.443071	1.947778
39	6	0	4.116697	-0.929184	0.136119
40	1	0	4.647074	-0.372564	-1.878187
41	1	0	3.371088	-1.272782	2.121482
42	1	0	0.743923	2.564737	1.847296
43	8	0	5.328526	-2.629165	1.290826
44	6	0	5.158405	-1.980516	0.281889
45	8	0	5.900195	-2.148286	-0.815794
46	1	0	6.555673	-2.844349	-0.648270

TS-CO/BCN$_2$/AB

```
Zero-point correction=                           0.368633 (Hartree/Particle)
Thermal correction to Energy=                    0.391402
Thermal correction to Enthalpy=                  0.392346
Thermal correction to Gibbs Free Energy=         0.314493
Sum of electronic and zero-point Energies=          -1221.015605
Sum of electronic and thermal Energies=             -1221.992836
Sum of electronic and thermal Enthalpies=           -1220.991892
Sum of electronic and thermal Free Energies=        -1221.069745
```

One imaginary frequency: -391.14i cm-1

Standard orientation:

Center Number	Atomic Number	Atomic Type	Coordinates (Angstroms) X	Y	Z
1	7	0	-1.153297	1.985744	-0.049777
2	7	0	-0.520041	2.147505	1.115118
3	6	0	-0.347424	2.336235	-1.070100
4	6	0	0.590067	3.264666	-0.499409
5	8	0	0.374152	3.172611	0.858276
6	8	0	1.422512	4.012596	-0.964117
7	6	0	1.165584	0.754344	-0.520075
8	6	0	0.957460	0.608009	0.691876
9	6	0	1.949124	0.408303	-1.723283
10	1	0	1.287001	0.092911	-2.534220
11	1	0	2.484970	1.295707	-2.077630
12	6	0	1.272317	-0.020793	1.983434
13	1	0	0.375175	-0.465524	2.422269
14	1	0	1.623655	0.745210	2.683357
15	6	0	2.356675	-1.092791	1.766442
16	1	0	2.575500	-1.569744	2.727102
17	1	0	1.950156	-1.864027	1.109125
18	6	0	3.632180	-0.499279	1.199637
19	6	0	2.944781	-0.715564	-1.378485
20	1	0	3.513389	-0.962750	-2.280589
21	1	0	2.378653	-1.607984	-1.101280
22	6	0	3.905825	-0.313759	-0.277731
23	1	0	4.028554	0.296689	1.823864
24	1	0	4.456557	0.592458	-0.514417
25	6	0	4.694544	-1.328807	0.517949

26	1	0	5.721148	-1.058808	0.743505
27	6	0	4.511858	-2.801782	0.292186
28	1	0	4.894834	-3.360523	1.152489
29	1	0	3.460944	-3.066385	0.158394
30	8	0	5.252163	-3.164508	-0.881737
31	1	0	5.202659	-4.121258	-0.983022
32	6	0	-2.169348	0.990562	-0.089939
33	6	0	-3.087100	0.935855	0.954553
34	6	0	-2.208867	0.092147	-1.151914
35	6	0	-4.069534	-0.041339	0.932797
36	1	0	-3.036104	1.655146	1.761728
37	6	0	-3.203883	-0.873613	-1.168707
38	1	0	-1.473159	0.134922	-1.945534
39	6	0	-4.131456	-0.944205	-0.130394
40	1	0	-4.791648	-0.092330	1.737015
41	1	0	-3.254960	-1.582939	-1.985172
42	1	0	-0.636755	2.272942	-2.106608
43	8	0	-5.277889	-2.802441	-1.093452
44	6	0	-5.177256	-2.000100	-0.191438
45	8	0	-6.008227	-1.983551	0.853801
46	1	0	-6.662163	-2.692635	0.745678

TS-CF/BCN$_1$/SB

```
Zero-point correction=                           0.358249 (Hartree/Particle)
Thermal correction to Energy=                    0.381796
Thermal correction to Enthalpy=                  0.382740
Thermal correction to Gibbs Free Energy=         0.301887
Sum of electronic and zero-point Energies=          -1369.494795
Sum of electronic and thermal Energies=             -1369.471249
Sum of electronic and thermal Enthalpies=           -1369.470305
Sum of electronic and thermal Free Energies=        -1369.551158
```

One imaginary frequency: -392.81i cm-1

Standard orientation:

Center Number	Atomic Number	Atomic Type	Coordinates (Angstroms) X	Y	Z
1	7	0	-0.589584	2.395931	-0.028282
2	7	0	0.009779	2.554304	1.156483
3	6	0	0.260316	2.715197	-1.023091
4	6	0	1.200730	3.626238	-0.431498
5	8	0	0.936390	3.554153	0.919295
6	8	0	2.065618	4.350361	-0.874683
7	6	0	1.424887	0.952682	0.798203
8	6	0	1.696760	1.091067	-0.402038
9	6	0	1.541857	0.216720	2.066306
10	1	0	1.859971	0.887734	2.868868
11	1	0	0.560017	-0.177142	2.350755
12	6	0	2.417760	0.622482	-1.602610
13	1	0	3.030967	1.427215	-2.018047
14	1	0	1.695883	0.341333	-2.377143
15	6	0	3.294382	-0.586812	-1.228080
16	1	0	3.821817	-0.925770	-2.125451
17	1	0	4.052649	-0.260192	-0.512864
18	6	0	2.470461	-1.730623	-0.669743
19	6	0	2.552692	-0.931081	1.887660
20	1	0	2.638633	-1.472975	2.834839
21	1	0	3.532429	-0.498637	1.674338
22	6	0	2.125746	-1.894085	0.796172
23	1	0	1.678709	-2.043082	-1.345653
24	1	0	1.135681	-2.305804	0.972964
25	6	0	3.084066	-2.854494	0.131639

26	1	0	2.694144	-3.848586	-0.067470
27	6	0	4.550613	-2.858032	0.470903
28	1	0	4.913830	-1.862444	0.720911
29	1	0	4.733540	-3.507571	1.333490
30	8	0	5.351833	-3.293923	-0.633998
31	1	0	5.067387	-4.183476	-0.872587
32	6	0	-1.602338	1.395765	-0.088672
33	6	0	-2.594777	1.396870	0.887975
34	6	0	-1.555416	0.433662	-1.089476
35	6	0	-3.562545	0.407286	0.856957
36	1	0	-2.608965	2.166086	1.649512
37	6	0	-2.537766	-0.549747	-1.120867
38	1	0	-0.762795	0.436099	-1.827716
39	6	0	-3.528565	-0.557628	-0.148744
40	1	0	-4.345837	0.390658	1.606314
41	1	0	-2.515656	-1.306796	-1.894115
42	1	0	0.007042	2.639977	-2.068396
43	6	0	-4.592210	-1.613925	-0.148992
44	9	0	-4.562485	-2.354812	0.974716
45	9	0	-4.481211	-2.464036	-1.177015
46	9	0	-5.827577	-1.083624	-0.212910

TS-CF/BCN$_1$/ST

```
Zero-point correction=                           0.358576 (Hartree/Particle)
Thermal correction to Energy=                    0.382055
Thermal correction to Enthalpy=                  0.382999
Thermal correction to Gibbs Free Energy=         0.302875
Sum of electronic and zero-point Energies=          -1369.494536
Sum of electronic and thermal Energies=             -1369.471057
Sum of electronic and thermal Enthalpies=           -1369.470113
Sum of electronic and thermal Free Energies=        -1369.550237
```

One imaginary frequency: -391.53i cm-1

Standard orientation:

Center Number	Atomic Number	Atomic Type	Coordinates (Angstroms) X	Y	Z
1	7	0	-0.614220	2.367498	0.149545
2	7	0	0.059862	2.407807	1.303065
3	6	0	0.168486	2.793958	-0.859971
4	6	0	1.144546	3.646468	-0.239054
5	8	0	0.968564	3.433597	1.111246
6	8	0	1.978675	4.418171	-0.660161
7	6	0	1.460726	0.865321	0.688289
8	6	0	1.647767	1.130872	-0.506682
9	6	0	1.685926	0.018592	1.869571
10	1	0	2.021285	0.626364	2.714434
11	1	0	0.741059	-0.447984	2.168726
12	6	0	2.310386	0.812486	-1.787505
13	1	0	2.849146	1.685984	-2.165652
14	1	0	1.555807	0.554609	-2.538440
15	6	0	3.277661	-0.365718	-1.572392
16	1	0	3.778425	-0.586198	-2.520601
17	1	0	4.048173	-0.052507	-0.865021
18	6	0	2.553813	-1.607636	-1.086953
19	6	0	2.733343	-1.057959	1.528346
20	1	0	2.890180	-1.683541	2.412671
21	1	0	3.682892	-0.562928	1.312485
22	6	0	2.294688	-1.931748	0.369787
23	1	0	1.748560	-1.902968	-1.754330
24	1	0	1.336375	-2.411828	0.549404
25	6	0	3.267055	-2.766697	-0.429675

26	1	0	2.920164	-3.756092	-0.713467
27	6	0	4.746977	-2.720839	-0.156131
28	1	0	5.297906	-3.092546	-1.026491
29	1	0	5.094142	-1.709444	0.048731
30	8	0	5.103348	-3.490205	0.999060
31	1	0	4.846590	-4.405310	0.838409
32	6	0	-1.633243	1.377625	0.051345
33	6	0	-2.544775	1.256715	1.097025
34	6	0	-1.673345	0.545182	-1.059616
35	6	0	-3.518243	0.275170	1.023113
36	1	0	-2.493756	1.927256	1.945143
37	6	0	-2.661479	-0.430261	-1.131993
38	1	0	-0.943365	0.641567	-1.853764
39	6	0	-3.571209	-0.560349	-0.091712
40	1	0	-4.239404	0.166023	1.825072
41	1	0	-2.705663	-1.086940	-1.991129
42	1	0	-0.151768	2.824509	-1.888984
43	6	0	-4.638557	-1.612078	-0.134202
44	9	0	-4.530399	-2.476468	0.892272
45	9	0	-4.609289	-2.338832	-1.257863
46	9	0	-5.871874	-1.080511	-0.045095

TS-CF/BCN$_1$/AT

```
Zero-point correction=                           0.358831 (Hartree/Particle)
Thermal correction to Energy=                    0.382208
Thermal correction to Enthalpy=                  0.383152
Thermal correction to Gibbs Free Energy=         0.303563
Sum of electronic and zero-point Energies=          -1369.494003
Sum of electronic and thermal Energies=             -1369.470626
Sum of electronic and thermal Enthalpies=           -1369.469682
Sum of electronic and thermal Free Energies=        -1369.549271
```

One imaginary frequency: -390.23i cm-1

Standard orientation:

Center Number	Atomic Number	Atomic Type	Coordinates (Angstroms) X	Y	Z
1	7	0	0.736438	2.171168	-0.193589
2	7	0	0.023065	2.144887	-1.322729
3	6	0	-0.025345	2.607483	0.827265
4	6	0	-1.051987	3.402429	0.209999
5	8	0	-0.915040	3.145785	-1.136861
6	8	0	-1.898449	4.159398	0.632845
7	6	0	-1.469780	0.891811	0.585525
8	6	0	-1.322533	0.595205	-0.607829
9	6	0	-2.156634	0.658762	1.872190
10	1	0	-1.428640	0.511138	2.674472
11	1	0	-2.747735	1.542181	2.137379
12	6	0	-1.662904	-0.218531	-1.784383
13	1	0	-0.761403	-0.659426	-2.218297
14	1	0	-2.103834	0.425132	-2.553425
15	6	0	-2.657311	-1.318976	-1.368866
16	1	0	-2.900460	-1.919452	-2.251105
17	1	0	-2.163214	-1.982274	-0.655309
18	6	0	-3.934297	-0.743511	-0.789353
19	6	0	-3.065597	-0.577695	1.742018
20	1	0	-3.552265	-0.755826	2.706351
21	1	0	-2.435325	-1.445060	1.534422
22	6	0	-4.125994	-0.398519	0.672655
23	1	0	-4.429476	-0.058045	-1.471681
24	1	0	-4.737207	0.483842	0.841098
25	6	0	-4.876260	-1.560519	0.062768

26	1	0	-5.935800	-1.397583	-0.112360
27	6	0	-4.556655	-2.984029	0.434483
28	1	0	-3.504289	-3.117313	0.679531
29	1	0	-5.143762	-3.281706	1.309705
30	8	0	-4.816591	-3.888899	-0.645867
31	1	0	-5.747944	-3.809542	-0.881381
32	6	0	1.810203	1.241495	-0.100316
33	6	0	2.663909	1.104832	-1.188140
34	6	0	1.964458	0.483150	1.057012
35	6	0	3.701761	0.185878	-1.117883
36	1	0	2.524372	1.715629	-2.070583
37	6	0	3.011690	-0.423062	1.124453
38	1	0	1.276435	0.587237	1.886992
39	6	0	3.869750	-0.568153	0.037352
40	1	0	4.376603	0.070806	-1.956110
41	1	0	3.151492	-1.021828	2.017212
42	1	0	0.329866	2.694238	1.841498
43	6	0	4.992366	-1.554925	0.150800
44	9	0	5.720533	-1.647640	-0.968726
45	9	0	4.546823	-2.793646	0.430073
46	9	0	5.844161	-1.232441	1.142695

TS-CF/BCN$_1$/AB

```
Zero-point correction=                           0.358641 (Hartree/Particle)
Thermal correction to Energy=                    0.382073
Thermal correction to Enthalpy=                  0.383017
Thermal correction to Gibbs Free Energy=         0.303560
Sum of electronic and zero-point Energies=          -1369.494166
Sum of electronic and thermal Energies=             -1369.470733
Sum of electronic and thermal Enthalpies=           -1369.469789
Sum of electronic and thermal Free Energies=        -1369.549247
```

One imaginary frequency: -390.84i cm-1

Standard orientation:

Center Number	Atomic Number	Atomic Type	Coordinates (Angstroms) X	Y	Z
1	7	0	-0.750964	2.162264	-0.021302
2	7	0	-0.103930	2.268468	1.142515
3	6	0	0.071800	2.474026	-1.040610
4	6	0	1.067151	3.335468	-0.462648
5	8	0	0.850965	3.239618	0.894835
6	8	0	1.942056	4.036936	-0.921736
7	6	0	1.486769	0.797044	-0.515743
8	6	0	1.269316	0.644991	0.693938
9	6	0	2.245389	0.419697	-1.725494
10	1	0	1.563696	0.171354	-2.543475
11	1	0	2.845881	1.272559	-2.060546
12	6	0	1.536873	-0.029233	1.973176
13	1	0	0.609070	-0.415539	2.403568
14	1	0	1.942408	0.695332	2.687739
15	6	0	2.540357	-1.173075	1.735552
16	1	0	2.723595	-1.681734	2.687342
17	1	0	2.079191	-1.901160	1.064908
18	6	0	3.856001	-0.664433	1.177906
19	6	0	3.154106	-0.781656	-1.404121
20	1	0	3.702552	-1.053831	-2.311634
21	1	0	2.522291	-1.633909	-1.143747
22	6	0	4.143160	-0.475584	-0.297176
23	1	0	4.310732	0.088634	1.815541
24	1	0	4.761828	0.390039	-0.517803
25	6	0	4.849453	-1.562543	0.478899

26	1	0	5.895047	-1.378616	0.708724
27	6	0	4.551915	-3.017537	0.232844
28	1	0	4.848173	-3.612055	1.103543
29	1	0	3.492093	-3.193401	0.055308
30	8	0	5.224780	-3.508788	-0.932747
31	1	0	6.172354	-3.396876	-0.795667
32	6	0	-1.827115	1.231679	-0.066125
33	6	0	-2.745862	1.232144	0.979988
34	6	0	-1.923910	0.341966	-1.129004
35	6	0	-3.786417	0.318407	0.957038
36	1	0	-2.650651	1.945096	1.788667
37	6	0	-2.977869	-0.563550	-1.150762
38	1	0	-1.189524	0.341483	-1.924856
39	6	0	-3.899074	-0.568946	-0.111274
40	1	0	-4.512689	0.305369	1.761087
41	1	0	-3.068045	-1.262448	-1.972571
42	1	0	-0.223834	2.439623	-2.076855
43	6	0	-5.014757	-1.570069	-0.098414
44	9	0	-4.757282	-2.597762	0.735010
45	9	0	-5.243959	-2.105328	-1.305013
46	9	0	-6.172762	-1.031350	0.317958

TS-CF/BCN$_2$/SB

```
Zero-point correction=                           0.358287 (Hartree/Particle)
Thermal correction to Energy=                    0.381775
Thermal correction to Enthalpy=                  0.382719
Thermal correction to Gibbs Free Energy=         0.302489
Sum of electronic and zero-point Energies=          -1369.494511
Sum of electronic and thermal Energies=             -1369.471022
Sum of electronic and thermal Enthalpies=           -1369.470078
Sum of electronic and thermal Free Energies=        -1369.550309
```

One imaginary frequency: -393.02i cm-1

Standard orientation:

Center Number	Atomic Number	Atomic Type	Coordinates (Angstroms) X	Y	Z
1	7	0	-0.586415	2.395500	-0.028501
2	7	0	0.012474	2.554349	1.156439
3	6	0	0.264496	2.712574	-1.023157
4	6	0	1.205724	3.623070	-0.432010
5	8	0	0.940541	3.552801	0.918667
6	8	0	2.071877	4.345522	-0.875474
7	6	0	1.426010	0.950745	0.800408
8	6	0	1.697668	1.086619	-0.400171
9	6	0	1.543361	0.216701	2.069606
10	1	0	1.863078	0.888645	2.870752
11	1	0	0.561499	-0.175840	2.355765
12	6	0	2.414730	0.613647	-1.601377
13	1	0	3.031278	1.415008	-2.018385
14	1	0	1.690098	0.335774	-2.374536
15	6	0	3.285787	-0.600211	-1.228407
16	1	0	3.806707	-0.943868	-2.127739
17	1	0	4.049448	-0.277505	-0.517034
18	6	0	2.457798	-1.738108	-0.664382
19	6	0	2.553223	-0.931937	1.890849
20	1	0	2.642288	-1.471760	2.838950
21	1	0	3.532075	-0.500080	1.672994
22	6	0	2.121233	-1.897111	0.803328
23	1	0	1.661559	-2.048153	-1.335983
24	1	0	1.131043	-2.306000	0.985592
25	6	0	3.069854	-2.864665	0.136287

26	1	0	2.673751	-3.855920	-0.059224
27	6	0	4.535325	-2.871545	0.461956
28	1	0	4.895772	-1.876639	0.731248
29	1	0	4.727217	-3.542164	1.306078
30	8	0	5.242046	-3.342560	-0.693795
31	1	0	6.175553	-3.411203	-0.465864
32	6	0	-1.600170	1.396292	-0.088797
33	6	0	-2.594339	1.400085	0.886059
34	6	0	-1.552479	0.432466	-1.087930
35	6	0	-3.563230	0.411580	0.854816
36	1	0	-2.609046	2.170561	1.646316
37	6	0	-2.535871	-0.549871	-1.119537
38	1	0	-0.758415	0.432712	-1.824634
39	6	0	-3.528533	-0.554968	-0.149289
40	1	0	-4.347974	0.397075	1.602692
41	1	0	-2.513116	-1.308257	-1.891454
42	1	0	0.011630	2.636691	-2.068518
43	6	0	-4.593398	-1.610059	-0.149949
44	9	0	-4.566290	-2.349726	0.974621
45	9	0	-4.481830	-2.461434	-1.176850
46	9	0	-5.828069	-1.078391	-0.216392

TS-CF/BCN$_2$/ST

```
Zero-point correction=                           0.358530 (Hartree/Particle)
Thermal correction to Energy=                    0.381987
Thermal correction to Enthalpy=                  0.382931
Thermal correction to Gibbs Free Energy=         0.302860
Sum of electronic and zero-point Energies=          -1369.494312
Sum of electronic and thermal Energies=             -1369.470856
Sum of electronic and thermal Enthalpies=           -1369.469911
Sum of electronic and thermal Free Energies=        -1369.549982
```

One imaginary frequency: -392.00i cm-1

Standard orientation:

Center Number	Atomic Number	Atomic Type	Coordinates (Angstroms) X	Y	Z
1	7	0	-0.590461	2.386051	0.149797
2	7	0	0.081985	2.421475	1.304787
3	6	0	0.196290	2.811055	-0.857270
4	6	0	1.174829	3.658089	-0.233109
5	8	0	0.995093	3.443664	1.116608
6	8	0	2.013031	4.427212	-0.651005
7	6	0	1.471011	0.871314	0.691268
8	6	0	1.667967	1.140512	-0.501373
9	6	0	1.669007	0.009794	1.866749
10	1	0	2.018469	0.601056	2.717569
11	1	0	0.709807	-0.431371	2.158987
12	6	0	2.332871	0.818313	-1.780081
13	1	0	2.894103	1.682602	-2.146485
14	1	0	1.578647	0.583844	-2.538992
15	6	0	3.271307	-0.383404	-1.566789
16	1	0	3.775966	-0.607592	-2.512069
17	1	0	4.041550	-0.094135	-0.849272
18	6	0	2.512493	-1.611313	-1.099774
19	6	0	2.685566	-1.094630	1.521321
20	1	0	2.815958	-1.733935	2.400054
21	1	0	3.651810	-0.627476	1.317372
22	6	0	2.229124	-1.942300	0.350365
23	1	0	1.705895	-1.878287	-1.777358
24	1	0	1.255585	-2.395399	0.517265
25	6	0	3.183507	-2.798603	-0.450036

26	1	0	2.809452	-3.773050	-0.747073
27	6	0	4.655628	-2.800052	-0.154574
28	1	0	5.206256	-3.206667	-1.009237
29	1	0	5.033122	-1.794655	0.042610
30	8	0	4.875074	-3.630034	0.994801
31	1	0	5.824687	-3.684141	1.147943
32	6	0	-1.610171	1.396927	0.049011
33	6	0	-2.524296	1.277331	1.092558
34	6	0	-1.646795	0.562361	-1.060467
35	6	0	-3.496417	0.294488	1.018441
36	1	0	-2.475839	1.949443	1.939589
37	6	0	-2.633545	-0.414519	-1.133079
38	1	0	-0.914838	0.657832	-1.852932
39	6	0	-3.545445	-0.543698	-0.094561
40	1	0	-4.219226	0.186092	1.819024
41	1	0	-2.674833	-1.073181	-1.990852
42	1	0	-0.121126	2.843532	-1.887113
43	6	0	-4.610322	-1.597976	-0.136402
44	9	0	-4.498764	-2.462605	0.889541
45	9	0	-4.580584	-2.324141	-1.260417
46	9	0	-5.844802	-1.069483	-0.045516

TS-CF/BCN$_2$/AT

```
Zero-point correction=                           0.358629 (Hartree/Particle)
Thermal correction to Energy=                    0.382078
Thermal correction to Enthalpy=                  0.383022
Thermal correction to Gibbs Free Energy=         0.303291
Sum of electronic and zero-point Energies=          -1369.493892
Sum of electronic and thermal Energies=             -1369.470442
Sum of electronic and thermal Enthalpies=           -1369.469498
Sum of electronic and thermal Free Energies=        -1369.549229
```

One imaginary frequency: -390.77i cm-1

Standard orientation:

Center Number	Atomic Number	Atomic Type	Coordinates (Angstroms) X	Y	Z
1	7	0	0.737535	2.163485	-0.194946
2	7	0	0.023212	2.136834	-1.323326
3	6	0	-0.023609	2.598482	0.826943
4	6	0	-1.051571	3.392888	0.210857
5	8	0	-0.915928	3.136729	-1.136082
6	8	0	-1.898337	4.148732	0.634973
7	6	0	-1.464918	0.881351	0.588209
8	6	0	-1.323763	0.586958	-0.606404
9	6	0	-2.147009	0.646707	1.877157
10	1	0	-1.416114	0.491321	2.675318
11	1	0	-2.731841	1.532274	2.149020
12	6	0	-1.675847	-0.218402	-1.785281
13	1	0	-0.780440	-0.666776	-2.224089
14	1	0	-2.112164	0.433569	-2.549971
15	6	0	-2.681034	-1.310315	-1.372663
16	1	0	-2.935126	-1.901389	-2.258148
17	1	0	-2.191547	-1.984457	-0.666061
18	6	0	-3.948569	-0.724713	-0.782944
19	6	0	-3.063555	-0.583822	1.744699
20	1	0	-3.546262	-0.765457	2.710392
21	1	0	-2.439827	-1.453494	1.527695
22	6	0	-4.128494	-0.390153	0.682523
23	1	0	-4.440161	-0.029481	-1.457788
24	1	0	-4.731119	0.496141	0.860698

25	6	0	-4.895814	-1.537899	0.068837
26	1	0	-5.953381	-1.361955	-0.099443
27	6	0	-4.588556	-2.964310	0.423056
28	1	0	-3.534488	-3.105588	0.670202
29	1	0	-5.183657	-3.269133	1.290204
30	8	0	-4.931843	-3.782381	-0.704119
31	1	0	-4.816414	-4.704575	-0.450575
32	6	0	1.813393	1.236259	-0.102345
33	6	0	2.666822	1.101096	-1.190553
34	6	0	1.970521	0.479321	1.055552
35	6	0	3.707529	0.185373	-1.119945
36	1	0	2.525025	1.710733	-2.073439
37	6	0	3.020540	-0.423584	1.123317
38	1	0	1.282689	0.582123	1.885861
39	6	0	3.878528	-0.566980	0.035945
40	1	0	4.382321	0.071625	-1.958393
41	1	0	3.162691	-1.020967	2.016630
42	1	0	0.332963	2.686218	1.840617
43	6	0	5.004363	-1.550004	0.149965
44	9	0	5.732404	-1.641548	-0.969740
45	9	0	4.562896	-2.789883	0.430666
46	9	0	5.855451	-1.223805	1.141234

TS-CF/BCN$_2$/AB

```
Zero-point correction=                           0.358342 (Hartree/Particle)
Thermal correction to Energy=                    0.381905
Thermal correction to Enthalpy=                  0.382849
Thermal correction to Gibbs Free Energy=         0.302362
Sum of electronic and zero-point Energies=          -1369.494139
Sum of electronic and thermal Energies=             -1369.470576
Sum of electronic and thermal Enthalpies=           -1369.469632
Sum of electronic and thermal Free Energies=        -1369.550119
```

One imaginary frequency: -391.41i cm-1

Standard orientation:

Center Number	Atomic Number	Atomic Type	Coordinates (Angstroms) X	Y	Z
1	7	0	0.754563	2.118995	0.024435
2	7	0	0.102548	2.236506	-1.135269
3	6	0	-0.063071	2.421560	1.050502
4	6	0	-1.059901	3.289954	0.485089
5	8	0	-0.850109	3.206776	-0.874144
6	8	0	-1.931902	3.987768	0.955018
7	6	0	-1.484591	0.755662	0.519624
8	6	0	-1.277123	0.613891	-0.693095
9	6	0	-2.244487	0.380488	1.729258
10	1	0	-1.564077	0.098553	2.537378
11	1	0	-2.816336	1.245409	2.083090
12	6	0	-1.567982	-0.038812	-1.978426
13	1	0	-0.652983	-0.444691	-2.418053
14	1	0	-1.957757	0.704351	-2.682602
15	6	0	-2.600594	-1.158187	-1.748043
16	1	0	-2.801840	-1.652018	-2.704012
17	1	0	-2.156171	-1.904918	-1.086932
18	6	0	-3.899202	-0.618698	-1.179504
19	6	0	-3.192565	-0.786950	1.396554
20	1	0	-3.746116	-1.052041	2.302955
21	1	0	-2.590468	-1.656426	1.122518
22	6	0	-4.174857	-0.434337	0.297646
23	1	0	-4.333419	0.154082	-1.807707
24	1	0	-4.763487	0.448747	0.530468

25	6	0	-4.920972	-1.489284	-0.487141
26	1	0	-5.959472	-1.267151	-0.710409
27	6	0	-4.671296	-2.950412	-0.249157
28	1	0	-5.029575	-3.533958	-1.103584
29	1	0	-3.608956	-3.164665	-0.114880
30	8	0	-5.392385	-3.335502	0.929455
31	1	0	-5.301892	-4.288421	1.037851
32	6	0	1.834506	1.192492	0.056249
33	6	0	2.752772	1.211663	-0.989909
34	6	0	1.935755	0.288725	1.106958
35	6	0	3.797881	0.302704	-0.980021
36	1	0	2.652608	1.933943	-1.789688
37	6	0	2.994664	-0.611009	1.116372
38	1	0	1.200567	0.272878	1.901956
39	6	0	3.912457	-0.601697	0.073643
40	1	0	4.523701	0.304605	-1.784433
41	1	0	3.088678	-1.320939	1.928343
42	1	0	0.237790	2.377727	2.084895
43	6	0	5.081060	-1.540451	0.093162
44	9	0	5.362522	-2.024243	-1.127696
45	9	0	4.884125	-2.594989	0.896235
46	9	0	6.204450	-0.934070	0.525627

TS-NO/BCN$_1$/SB

```
Zero-point correction=                           0.356629 (Hartree/Particle)
Thermal correction to Energy=                    0.378996
Thermal correction to Enthalpy=                  0.379940
Thermal correction to Gibbs Free Energy=         0.302557
Sum of electronic and zero-point Energies=          -1236.936386
Sum of electronic and thermal Energies=             -1236.914020
Sum of electronic and thermal Enthalpies=           -1236.913075
Sum of electronic and thermal Free Energies=        -1236.990458
```

One imaginary frequency: -381.46i cm-1

Standard orientation:

Center Number	Atomic Number	Atomic Type	Coordinates (Angstroms) X	Y	Z
1	7	0	-1.155923	2.091864	-0.027215
2	7	0	-0.575042	2.312001	1.155338
3	6	0	-0.354289	2.504886	-1.027271
4	6	0	0.491608	3.504264	-0.435688
5	8	0	0.244447	3.398255	0.916567
6	8	0	1.275842	4.313892	-0.879706
7	6	0	1.024276	0.864953	0.775919
8	6	0	1.262251	1.037842	-0.425873
9	6	0	1.279281	0.182125	2.053082
10	1	0	1.452151	0.913554	2.847147
11	1	0	0.397899	-0.400395	2.341622
12	6	0	2.046169	0.697711	-1.629979
13	1	0	2.479782	1.600242	-2.070050
14	1	0	1.386444	0.260198	-2.386838
15	6	0	3.154580	-0.299564	-1.244355
16	1	0	3.733439	-0.540688	-2.141591
17	1	0	3.834765	0.190868	-0.544240
18	6	0	2.590076	-1.578257	-0.656842
19	6	0	2.505769	-0.735211	1.888101
20	1	0	2.703350	-1.231491	2.843514
21	1	0	3.372427	-0.110157	1.663270
22	6	0	2.289836	-1.783976	0.813711
23	1	0	1.881302	-2.064376	-1.321960
24	1	0	1.409802	-2.392744	1.002367

25	6	0	3.428614	-2.532387	0.160656
26	1	0	3.256415	-3.589487	-0.020200
27	6	0	4.864133	-2.221315	0.491474
28	1	0	5.008704	-1.171789	0.742204
29	1	0	5.186196	-2.817705	1.351641
30	8	0	5.734224	-2.474350	-0.618533
31	1	0	5.643973	-3.403199	-0.859878
32	6	0	-2.080684	1.014421	-0.084560
33	6	0	-3.012008	0.887186	0.942672
34	6	0	-2.015465	0.110673	-1.140891
35	6	0	-3.904260	-0.170814	0.913638
36	1	0	-3.043267	1.613261	1.744337
37	6	0	-2.916387	-0.942441	-1.178790
38	1	0	-1.269380	0.213530	-1.918636
39	6	0	-3.838123	-1.060663	-0.149839
40	1	0	-4.642992	-0.294226	1.693324
41	1	0	-2.891759	-1.662713	-1.984893
42	1	0	-0.605473	2.410220	-2.071438
43	7	0	-4.789609	-2.180069	-0.187376
44	8	0	-4.770900	-2.916715	-1.153534
45	8	0	-5.551366	-2.316841	0.749465

TS-NO/BCN$_1$/ST

```
Zero-point correction=                           0.356551 (Hartree/Particle)
Thermal correction to Energy=                    0.378891
Thermal correction to Enthalpy=                  0.379835
Thermal correction to Gibbs Free Energy=         0.302912
Sum of electronic and zero-point Energies=          -1236.936458
Sum of electronic and thermal Energies=             -1236.914118
Sum of electronic and thermal Enthalpies=           -1236.913174
Sum of electronic and thermal Free Energies=        -1236.990097
```

One imaginary frequency: -382.49i cm-1

Standard orientation:

Center Number	Atomic Number	Atomic Type	Coordinates (Angstroms) X	Y	Z
1	7	0	-1.164151	2.086480	0.140830
2	7	0	-0.501599	2.205023	1.294652
3	6	0	-0.439309	2.600115	-0.871052
4	6	0	0.439436	3.553005	-0.250892
5	8	0	0.290451	3.320318	1.099852
6	8	0	1.184095	4.410528	-0.672477
7	6	0	1.079972	0.824034	0.671338
8	6	0	1.226078	1.107915	-0.524078
9	6	0	1.434621	0.038028	1.862352
10	1	0	1.655272	0.701735	2.702803
11	1	0	0.582270	-0.581595	2.160686
12	6	0	1.929143	0.891458	-1.804329
13	1	0	2.310207	1.839291	-2.194939
14	1	0	1.228139	0.498707	-2.548534
15	6	0	3.084517	-0.100523	-1.576961
16	1	0	3.614861	-0.243554	-2.523929
17	1	0	3.790729	0.349582	-0.876110
18	6	0	2.587675	-1.443227	-1.074898
19	6	0	2.655510	-0.843245	1.535937
20	1	0	2.915449	-1.422542	2.427544
21	1	0	3.504276	-0.191349	1.317183
22	6	0	2.383990	-1.792162	0.385301
23	1	0	1.850535	-1.884938	-1.740006
24	1	0	1.526045	-2.433630	0.567779
25	6	0	3.492964	-2.448812	-0.402465

26	1	0	3.331045	-3.487541	-0.675792
27	6	0	4.939085	-2.135473	-0.125148
28	1	0	5.549735	-2.384647	-0.999426
29	1	0	5.092785	-1.080912	0.098191
30	8	0	5.428006	-2.847090	1.018059
31	1	0	5.337644	-3.789863	0.838873
32	6	0	-2.082692	1.006201	0.047839
33	6	0	-2.949622	0.781381	1.113916
34	6	0	-2.074736	0.196751	-1.084253
35	6	0	-3.835861	-0.280008	1.046852
36	1	0	-2.937406	1.436648	1.974874
37	6	0	-2.969661	-0.859534	-1.158972
38	1	0	-1.376465	0.374830	-1.892063
39	6	0	-3.828064	-1.074761	-0.091473
40	1	0	-4.526348	-0.477739	1.854930
41	1	0	-2.988224	-1.508794	-2.023345
42	1	0	-0.763820	2.597323	-1.899037
43	7	0	-4.773436	-2.197456	-0.169161
44	8	0	-4.805453	-2.850805	-1.193191
45	8	0	-5.479689	-2.419975	0.794257

TS-NO/BCN$_1$/AT

```
Zero-point correction=                           0.356368 (Hartree/Particle)
Thermal correction to Energy=                    0.378821
Thermal correction to Enthalpy=                  0.379765
Thermal correction to Gibbs Free Energy=         0.302317
Sum of electronic and zero-point Energies=          -1236.936431
Sum of electronic and thermal Energies=             -1236.913978
Sum of electronic and thermal Enthalpies=           -1236.913034
Sum of electronic and thermal Free Energies=        -1236.990482
```

One imaginary frequency: -383.32i cm-1

Standard orientation:

Center Number	Atomic Number	Atomic Type	Coordinates (Angstroms) X	Y	Z
1	7	0	1.194808	1.960609	-0.175587
2	7	0	0.499833	1.988555	-1.315528
3	6	0	0.460660	2.464831	0.834187
4	6	0	-0.494530	3.331536	0.200059
5	8	0	-0.361664	3.055304	-1.143976
6	8	0	-1.285785	4.153157	0.607742
7	6	0	-1.115432	0.856132	0.586239
8	6	0	-0.983700	0.542180	-0.603386
9	6	0	-1.830646	0.695199	1.868106
10	1	0	-1.127093	0.480473	2.676852
11	1	0	-2.336801	1.631926	2.125721
12	6	0	-1.384485	-0.237810	-1.783376
13	1	0	-0.524509	-0.763682	-2.206447
14	1	0	-1.754498	0.442795	-2.557879
15	6	0	-2.484136	-1.237019	-1.375312
16	1	0	-2.774299	-1.814877	-2.258444
17	1	0	-2.063253	-1.941587	-0.654389
18	6	0	-3.705929	-0.539337	-0.811038
19	6	0	-2.852981	-0.448415	1.730110
20	1	0	-3.365607	-0.575772	2.688975
21	1	0	-2.307787	-1.373585	1.531258
22	6	0	-3.878700	-0.170568	0.647906
23	1	0	-4.126181	0.187622	-1.500644
24	1	0	-4.402388	0.768185	0.806219
25	6	0	-4.731042	-1.257199	0.034497
26	1	0	-5.768391	-0.994700	-0.152312

27	6	0	-4.553931	-2.701724	0.418953
28	1	0	-3.517734	-2.938298	0.655325
29	1	0	-5.158920	-2.930033	1.302835
30	8	0	-4.915946	-3.585539	-0.649110
31	1	0	-5.840152	-3.421652	-0.868582
32	6	0	2.194675	0.957576	-0.066041
33	6	0	3.023949	0.727334	-1.160966
34	6	0	2.303608	0.224555	1.111903
35	6	0	3.989637	-0.260737	-1.076595
36	1	0	2.920855	1.321899	-2.058875
37	6	0	3.277635	-0.757823	1.202706
38	1	0	1.634998	0.402983	1.944353
39	6	0	4.096956	-0.979488	0.106312
40	1	0	4.651878	-0.460956	-1.907367
41	1	0	3.387262	-1.346213	2.103083
42	1	0	0.808826	2.529872	1.852407
43	7	0	5.125650	-2.024437	0.200957
44	8	0	5.221085	-2.643170	1.242446
45	8	0	5.833824	-2.221355	-0.766673

TS-NO/BCN$_1$/AB

```
Zero-point correction=                           0.356541 (Hartree/Particle)
Thermal correction to Energy=                    0.378955
Thermal correction to Enthalpy=                  0.379900
Thermal correction to Gibbs Free Energy=         0.302418
Sum of electronic and zero-point Energies=          -1236.936246
Sum of electronic and thermal Energies=             -1236.913831
Sum of electronic and thermal Enthalpies=           -1236.912887
Sum of electronic and thermal Free Energies=        -1236.990369
```

One imaginary frequency: -384.64i cm-1

Standard orientation:

Center Number	Atomic Number	Atomic Type	Coordinates (Angstroms) X	Y	Z
1	7	0	1.199739	1.970705	0.039319
2	7	0	0.566698	2.144207	-1.123803
3	6	0	0.407105	2.337354	1.064172
4	6	0	-0.522020	3.275161	0.496357
5	8	0	-0.313367	3.177044	-0.862884
6	8	0	-1.343201	4.033813	0.962728
7	6	0	-1.134387	0.763381	0.520986
8	6	0	-0.934348	0.613433	-0.690928
9	6	0	-1.911935	0.426795	1.730425
10	1	0	-1.247164	0.109651	2.538381
11	1	0	-2.441306	1.317938	2.084837
12	6	0	-1.259050	-0.010031	-1.982263
13	1	0	-0.368070	-0.462892	-2.425153
14	1	0	-1.606535	0.759908	-2.679665
15	6	0	-2.352809	-1.071803	-1.760576
16	1	0	-2.582190	-1.543916	-2.721102
17	1	0	-1.949892	-1.848934	-1.107748
18	6	0	-3.618957	-0.468106	-1.183557
19	6	0	-2.914398	-0.693117	1.391360
20	1	0	-3.478091	-0.939486	2.296816
21	1	0	-2.352773	-1.586935	1.109871
22	6	0	-3.881153	-0.286404	0.297108
23	1	0	-4.013899	0.332587	-1.802694
24	1	0	-4.426032	0.622072	0.539032
25	6	0	-4.678876	-1.295686	-0.495061
26	1	0	-5.706996	-1.021168	-0.713121
27	6	0	-4.503186	-2.775043	-0.280217

28	1	0	-4.866076	-3.324273	-1.155498
29	1	0	-3.459995	-3.046748	-0.126858
30	8	0	-5.195639	-3.228739	0.889301
31	1	0	-6.127188	-3.003276	0.786013
32	6	0	2.197334	0.959817	0.073880
33	6	0	3.092412	0.875353	-0.989533
34	6	0	2.238303	0.075617	1.147891
35	6	0	4.055916	-0.118454	-0.979609
36	1	0	3.041036	1.584953	-1.804698
37	6	0	3.211482	-0.911543	1.166746
38	1	0	1.518451	0.140757	1.953897
39	6	0	4.096285	-0.987777	0.102053
40	1	0	4.768465	-0.207275	-1.787920
41	1	0	3.269323	-1.614846	1.985963
42	1	0	0.700767	2.270631	2.099192
43	7	0	5.123419	-2.038862	0.118683
44	8	0	5.196367	-2.754344	1.098150
45	8	0	5.852449	-2.143371	-0.847838

TS-NO/BCN$_2$/SB

```
Zero-point correction=                           0.356518 (Hartree/Particle)
Thermal correction to Energy=                    0.378923
Thermal correction to Enthalpy=                  0.379868
Thermal correction to Gibbs Free Energy=         0.302505
Sum of electronic and zero-point Energies=          -1236.936241
Sum of electronic and thermal Energies=             -1236.913836
Sum of electronic and thermal Enthalpies=           -1236.912892
Sum of electronic and thermal Free Energies=        -1236.990255
```

One imaginary frequency: -381.88i cm-1

Standard orientation:

Center Number	Atomic Number	Atomic Type	Coordinates (Angstroms) X	Y	Z
1	7	0	-1.149596	2.096908	-0.030222
2	7	0	-0.570792	2.322185	1.152321
3	6	0	-0.346880	2.506891	-1.030692
4	6	0	0.497142	3.509410	-0.441696
5	8	0	0.248283	3.408236	0.910459
6	8	0	1.281348	4.317990	-0.887674
7	6	0	1.031200	0.875189	0.780405
8	6	0	1.266993	1.039976	-0.422917
9	6	0	1.288329	0.200227	2.061349
10	1	0	1.470127	0.936439	2.848990
11	1	0	0.404872	-0.374541	2.358899
12	6	0	2.042186	0.685466	-1.628523
13	1	0	2.485689	1.580604	-2.073782
14	1	0	1.373817	0.253821	-2.381159
15	6	0	3.138940	-0.325143	-1.244186
16	1	0	3.706786	-0.580771	-2.144385
17	1	0	3.831930	0.159821	-0.552787
18	6	0	2.561237	-1.591380	-0.642789
19	6	0	2.507933	-0.726217	1.895748
20	1	0	2.709420	-1.216128	2.853645
21	1	0	3.377008	-0.109217	1.658809
22	6	0	2.274928	-1.781985	0.832003
23	1	0	1.839998	-2.072855	-1.297666
24	1	0	1.390713	-2.380382	1.033650
25	6	0	3.395847	-2.551333	0.174002
26	1	0	3.207596	-3.606253	0.002661
27	6	0	4.835914	-2.254144	0.478223
28	1	0	4.989268	-1.205837	0.742113

29	1	0	5.173651	-2.868197	1.319501
30	8	0	5.607253	-2.574228	-0.688145
31	1	0	6.539084	-2.463970	-0.470069
32	6	0	-2.072730	1.017876	-0.085996
33	6	0	-3.006598	0.892560	0.939117
34	6	0	-2.003612	0.110897	-1.139382
35	6	0	-3.897635	-0.166541	0.910724
36	1	0	-3.040958	1.621002	1.738510
37	6	0	-2.903280	-0.943263	-1.176696
38	1	0	-1.255658	0.212087	-1.915545
39	6	0	-3.827778	-1.059363	-0.149997
40	1	0	-4.638406	-0.288491	1.688705
41	1	0	-2.875570	-1.665872	-1.980610
42	1	0	-0.596925	2.408754	-2.074815
43	7	0	-4.778229	-2.179654	-0.187203
44	8	0	-4.756917	-2.918197	-1.151851
45	8	0	-5.541834	-2.315154	0.748321

TS-NO/BCN$_2$/ST

```
Zero-point correction=                           0.356171 (Hartree/Particle)
Thermal correction to Energy=                    0.378682
Thermal correction to Enthalpy=                  0.379627
Thermal correction to Gibbs Free Energy=         0.301587
Sum of electronic and zero-point Energies=          -1236.936548
Sum of electronic and thermal Energies=             -1236.914036
Sum of electronic and thermal Enthalpies=           -1236.913092
Sum of electronic and thermal Free Energies=        -1236.991131
```

One imaginary frequency: -382.57i cm-1

Standard orientation:

Center Number	Atomic Number	Atomic Type	Coordinates (Angstroms) X	Y	Z
1	7	0	-1.151129	2.104088	0.143162
2	7	0	-0.489237	2.220331	1.297868
3	6	0	-0.426249	2.622026	-0.866566
4	6	0	0.451023	3.573880	-0.243185
5	8	0	0.301240	3.337035	1.106896
6	8	0	1.194802	4.433871	-0.661471
7	6	0	1.088732	0.839462	0.673202
8	6	0	1.241007	1.129221	-0.520048
9	6	0	1.423775	0.035511	1.857858
10	1	0	1.660553	0.686082	2.704146
11	1	0	0.555437	-0.564487	2.150350
12	6	0	1.945531	0.912353	-1.799432
13	1	0	2.345934	1.856664	-2.179032
14	1	0	1.240903	0.539821	-2.550572
15	6	0	3.080828	-0.103562	-1.575760
16	1	0	3.614793	-0.246932	-2.520648
17	1	0	3.790287	0.325374	-0.865184
18	6	0	2.552614	-1.440943	-1.092105
19	6	0	2.621237	-0.874876	1.524776
20	1	0	2.861129	-1.472100	2.410041
21	1	0	3.489171	-0.244839	1.316818
22	6	0	2.328090	-1.801522	0.361466
23	1	0	1.809870	-1.857321	-1.767222
24	1	0	1.451818	-2.421109	0.531608
25	6	0	3.424343	-2.480206	-0.427256
26	1	0	3.234469	-3.509613	-0.713470
27	6	0	4.870587	-2.210870	-0.127218
28	1	0	5.490465	-2.510742	-0.978592
29	1	0	5.054527	-1.152547	0.068844

30	8	0	5.233159	-2.983649	1.025377
31	1	0	6.175372	-2.861275	1.184234
32	6	0	-2.064571	1.019512	0.047287
33	6	0	-2.931260	0.789027	1.112355
34	6	0	-2.050372	0.210427	-1.085020
35	6	0	-3.810244	-0.278315	1.044525
36	1	0	-2.923946	1.444104	1.973514
37	6	0	-2.938338	-0.851691	-1.160681
38	1	0	-1.352436	0.393112	-1.892091
39	6	0	-3.795982	-1.072970	-0.093792
40	1	0	-4.499973	-0.480921	1.852050
41	1	0	-2.951812	-1.501027	-2.025095
42	1	0	-0.749476	2.621017	-1.894952
43	7	0	-4.733017	-2.202622	-0.171910
44	8	0	-4.763823	-2.852677	-1.198064
45	8	0	-5.433866	-2.433908	0.793385

TS-NO/BCN$_2$/AT

```
Zero-point correction=                           0.356377 (Hartree/Particle)
Thermal correction to Energy=                    0.378810
Thermal correction to Enthalpy=                  0.379754
Thermal correction to Gibbs Free Energy=         0.302399
Sum of electronic and zero-point Energies=          -1236.936126
Sum of electronic and thermal Energies=             -1236.913693
Sum of electronic and thermal Enthalpies=           -1236.912749
Sum of electronic and thermal Free Energies=        -1236.990104
```

One imaginary frequency: -383.36i cm-1

Standard orientation:

Center Number	Atomic Number	Atomic Type	Coordinates (Angstroms) X	Y	Z
1	7	0	1.190944	1.960526	-0.178242
2	7	0	0.494791	1.986441	-1.317443
3	6	0	0.457565	2.465608	0.831670
4	6	0	-0.498977	3.330760	0.197341
5	8	0	-0.367466	3.052713	-1.146384
6	8	0	-1.290387	4.152312	0.604798
7	6	0	-1.116701	0.854368	0.588172
8	6	0	-0.988121	0.540534	-0.601818
9	6	0	-1.828132	0.691482	1.871940
10	1	0	-1.122029	0.477226	2.678574
11	1	0	-2.335223	1.627099	2.131717
12	6	0	-1.393776	-0.236016	-1.782365
13	1	0	-0.536521	-0.763998	-2.208301
14	1	0	-1.762736	0.447672	-2.554676
15	6	0	-2.496517	-1.232292	-1.375276
16	1	0	-2.792143	-1.804024	-2.260527
17	1	0	-2.076858	-1.942627	-0.659201
18	6	0	-3.713414	-0.532666	-0.803165
19	6	0	-2.848512	-0.454040	1.734951
20	1	0	-3.356901	-0.585944	2.695478
21	1	0	-2.301941	-1.376971	1.530335
22	6	0	-3.879369	-0.173524	0.658411
23	1	0	-4.134460	0.198853	-1.487281
24	1	0	-4.402566	0.764227	0.823828
25	6	0	-4.738152	-1.252971	0.042531
26	1	0	-5.774397	-0.986487	-0.138840
27	6	0	-4.560012	-2.697750	0.409871
28	1	0	-3.523558	-2.928511	0.664306
29	1	0	-5.184084	-2.942170	1.275853
30	8	0	-4.967333	-3.491981	-0.712933

31	1	0	-4.911926	-4.419555	-0.458826
32	6	0	2.191997	0.958755	-0.068109
33	6	0	3.019241	0.726101	-1.164072
34	6	0	2.304342	0.229432	1.111844
35	6	0	3.986399	-0.260465	-1.078635
36	1	0	2.913509	1.317684	-2.063641
37	6	0	3.279856	-0.751336	1.203689
38	1	0	1.637346	0.409513	1.945219
39	6	0	4.097209	-0.975328	0.106307
40	1	0	4.647131	-0.462467	-1.910182
41	1	0	3.392228	-1.336651	2.105732
42	1	0	0.806961	2.532955	1.849318
43	7	0	5.127454	-2.018598	0.202130
44	8	0	5.225192	-2.634587	1.245046
45	8	0	5.834531	-2.216997	-0.765994

TS-NO/BCN$_2$/AB

```
Zero-point correction=                           0.356362 (Hartree/Particle)
Thermal correction to Energy=                    0.378840
Thermal correction to Enthalpy=                  0.379785
Thermal correction to Gibbs Free Energy=         0.302050
Sum of electronic and zero-point Energies=          -1236.936105
Sum of electronic and thermal Energies=             -1236.913627
Sum of electronic and thermal Enthalpies=           -1236.912683
Sum of electronic and thermal Free Energies=        -1236.990418
```

One imaginary frequency: -384.65i cm-1

Standard orientation:

Center Number	Atomic Number	Atomic Type	Coordinates (Angstroms) X	Y	Z
1	7	0	1.196328	1.972403	0.045402
2	7	0	0.565338	2.150077	-1.118231
3	6	0	0.401957	2.335694	1.070106
4	6	0	-0.525810	3.275840	0.503906
5	8	0	-0.314827	3.182305	-0.855309
6	8	0	-1.347448	4.033244	0.971473
7	6	0	-1.139176	0.764409	0.518958
8	6	0	-0.936705	0.618073	-0.692999
9	6	0	-1.919315	0.425639	1.726071
10	1	0	-1.256357	0.106557	2.534768
11	1	0	-2.448686	1.316511	2.081181
12	6	0	-1.258349	-0.003340	-1.986123
13	1	0	-0.365539	-0.452253	-2.429344
14	1	0	-1.607976	0.766926	-2.682084
15	6	0	-2.348341	-1.069476	-1.766726
16	1	0	-2.574194	-1.542811	-2.727521
17	1	0	-1.943268	-1.844457	-1.112928
18	6	0	-3.617888	-0.470000	-1.192731
19	6	0	-2.922395	-0.692763	1.383734
20	1	0	-3.489502	-0.937202	2.287502
21	1	0	-2.361829	-1.588007	1.104281
22	6	0	-3.884775	-0.285250	0.286136
23	1	0	-4.012943	0.329019	-1.813875
24	1	0	-4.430384	0.623306	0.525682
25	6	0	-4.681466	-1.295354	-0.507913
26	1	0	-5.707696	-1.020222	-0.728900
27	6	0	-4.504802	-2.769577	-0.285366
28	1	0	-4.893980	-3.324936	-1.145078
29	1	0	-3.454636	-3.039489	-0.156415
30	8	0	-5.241964	-3.130827	0.890955
31	1	0	-5.195909	-4.087905	0.990806

32	6	0	2.193866	0.961378	0.077933
33	6	0	3.091616	0.882076	-0.983629
34	6	0	2.232265	0.072052	1.147814
35	6	0	4.055093	-0.111770	-0.976207
36	1	0	3.042184	1.595616	-1.795476
37	6	0	3.205352	-0.915258	1.164183
38	1	0	1.510615	0.133437	1.952529
39	6	0	4.092693	-0.986431	0.101243
40	1	0	4.769598	-0.196693	-1.783218
41	1	0	3.261294	-1.622423	1.980206
42	1	0	0.693823	2.265405	2.105406
43	7	0	5.119595	-2.037795	0.115156
44	8	0	5.189656	-2.758530	1.090981
45	8	0	5.851290	-2.137296	-0.849869

TS-MeF/BCN$_1$/SB

```
Zero-point correction=                           0.373128 (Hartree/Particle)
Thermal correction to Energy=                    0.395747
Thermal correction to Enthalpy=                  0.396692
Thermal correction to Gibbs Free Energy=         0.319655
Sum of electronic and zero-point Energies=          -1170.960451
Sum of electronic and thermal Energies=             -1170.937832
Sum of electronic and thermal Enthalpies=           -1170.936887
Sum of electronic and thermal Free Energies=        -1171.013924
```

One imaginary frequency: -358.98i cm-1

Standard orientation:

Center Number	Atomic Number	Atomic Type	Coordinates (Angstroms) X	Y	Z
1	7	0	2.036700	-1.193113	0.186436
2	7	0	1.604325	-1.542142	1.403101
3	6	0	1.439246	-1.973384	-0.724681
4	6	0	1.084436	-3.194396	-0.053237
5	8	0	1.263750	-2.886673	1.261796
6	8	0	0.710186	-4.274239	-0.450912
7	6	0	-0.469665	-0.838960	0.990208
8	6	0	-0.631045	-1.205417	-0.176733
9	6	0	-0.903554	-0.104447	2.186122
10	1	0	-0.907085	-0.756945	3.063014
11	1	0	-0.198280	0.707833	2.392158
12	6	0	-1.447028	-1.235457	-1.406107
13	1	0	-1.635281	-2.265891	-1.720208
14	1	0	-0.904408	-0.746381	-2.222747
15	6	0	-2.772789	-0.497722	-1.134882
16	1	0	-3.373695	-0.513199	-2.049614
17	1	0	-3.329199	-1.049197	-0.373606
18	6	0	-2.543019	0.940203	-0.711305
19	6	0	-2.313685	0.458328	1.919760
20	1	0	-2.646566	1.005395	2.807422
21	1	0	-2.998657	-0.380671	1.780977
22	6	0	-2.331884	1.388342	0.720753
23	1	0	-1.955555	1.498152	-1.436036
24	1	0	-1.625254	2.207556	0.824292
25	6	0	-3.601988	1.757420	-0.009250
26	1	0	-3.685035	2.795050	-0.319464
27	6	0	-4.926512	1.152063	0.371471
28	1	0	-4.818430	0.133820	0.742150
29	1	0	-5.396017	1.747285	1.161733
30	8	0	-5.813316	1.062098	-0.750362
31	1	0	-5.952004	1.953751	-1.089344
32	6	0	2.448322	0.163823	0.005491

33	6	0	3.426505	0.675866	0.850187
34	6	0	1.836995	0.947168	-0.966085
35	6	0	3.795272	2.005707	0.712858
36	1	0	3.886610	0.039164	1.596017
37	6	0	2.231571	2.274257	-1.093038
38	1	0	1.059570	0.541686	-1.603111
39	6	0	3.209582	2.822105	-0.260744
40	1	0	4.555516	2.417187	1.367700
41	1	0	1.760715	2.896326	-1.846070
42	6	0	3.644869	4.252375	-0.418132
43	1	0	3.817685	4.716787	0.554146
44	1	0	4.582229	4.303250	-0.979303
45	1	0	2.896803	4.832306	-0.959480
46	9	0	1.671159	-1.874801	-2.013055

TS-MeF/BCN$_1$/ST

```
Zero-point correction=                           0.373196 (Hartree/Particle)
Thermal correction to Energy=                    0.395791
Thermal correction to Enthalpy=                  0.396735
Thermal correction to Gibbs Free Energy=         0.320490
Sum of electronic and zero-point Energies=          -1170.960397
Sum of electronic and thermal Energies=             -1170.937802
Sum of electronic and thermal Enthalpies=           -1170.936858
Sum of electronic and thermal Free Energies=        -1171.013103
```

One imaginary frequency: -359.40i cm-1

Standard orientation:

Center Number	Atomic Number	Atomic Type	Coordinates (Angstroms) X	Y	Z
1	7	0	2.068683	-1.087647	0.333810
2	7	0	1.544277	-1.408901	1.521451
3	6	0	1.613770	-1.948496	-0.586799
4	6	0	1.275058	-3.158799	0.112069
5	8	0	1.306911	-2.779016	1.420077
6	8	0	1.010759	-4.279046	-0.261367
7	6	0	-0.520117	-0.869665	0.880704
8	6	0	-0.543797	-1.303905	-0.273875
9	6	0	-1.104678	-0.101183	1.987666
10	1	0	-1.161872	-0.708471	2.894653
11	1	0	-0.461565	0.755821	2.215134
12	6	0	-1.237102	-1.450309	-1.568523
13	1	0	-1.332160	-2.505529	-1.839327
14	1	0	-0.652831	-0.967935	-2.360058
15	6	0	-2.625015	-0.790534	-1.453650
16	1	0	-3.145218	-0.898565	-2.410684
17	1	0	-3.204783	-1.336259	-0.706326
18	6	0	-2.518837	0.682274	-1.103428
19	6	0	-2.507286	0.376421	1.561443
20	1	0	-2.940024	0.960577	2.379639
21	1	0	-3.144326	-0.499418	1.418730
22	6	0	-2.458886	1.229617	0.308425
23	1	0	-1.902617	1.229672	-1.812079
24	1	0	-1.803613	2.090011	0.416774
25	6	0	-3.676407	1.480195	-0.549778
26	1	0	-3.784007	2.488548	-0.939067
27	6	0	-4.997672	0.830614	-0.236421
28	1	0	-5.631277	0.822692	-1.129625
29	1	0	-4.878527	-0.199499	0.095776
30	8	0	-5.681188	1.502515	0.828580
31	1	0	-5.822636	2.416814	0.557898
32	6	0	2.410450	0.284116	0.121240

33	6	0	3.284554	0.892792	1.015255
34	6	0	1.836822	0.984328	-0.932793
35	6	0	3.585679	2.235047	0.842710
36	1	0	3.718884	0.318900	1.824796
37	6	0	2.162816	2.326852	-1.092790
38	1	0	1.139749	0.504247	-1.609735
39	6	0	3.035502	2.970069	-0.213565
40	1	0	4.265423	2.721100	1.534056
41	1	0	1.720923	2.884495	-1.910953
42	6	0	3.397270	4.417074	-0.401116
43	1	0	3.358295	4.953579	0.548786
44	1	0	4.416482	4.506532	-0.786967
45	1	0	2.723059	4.901623	-1.107694
46	9	0	1.963037	-1.893085	-1.851056

TS-MeF/BCN$_1$/AT

```
Zero-point correction=                           0.372953 (Hartree/Particle)
Thermal correction to Energy=                    0.395670
Thermal correction to Enthalpy=                  0.396614
Thermal correction to Gibbs Free Energy=         0.319369
Sum of electronic and zero-point Energies=          -1170.960222
Sum of electronic and thermal Energies=             -1170.937505
Sum of electronic and thermal Enthalpies=           -1170.936561
Sum of electronic and thermal Free Energies=        -1171.013806
```

One imaginary frequency: -354.98i cm-1

Standard orientation:

Center Number	Atomic Number	Atomic Type	Coordinates (Angstroms) X	Y	Z
1	7	0	1.919455	1.221523	-0.388655
2	7	0	1.259872	1.328180	-1.545129
3	6	0	1.305388	1.973304	0.534855
4	6	0	0.612693	3.013027	-0.180174
5	8	0	0.664547	2.588491	-1.472749
6	8	0	0.079316	4.039057	0.175145
7	6	0	-0.621108	0.779091	0.410306
8	6	0	-0.585242	0.330421	-0.737980
9	6	0	-1.313391	0.910570	1.707308
10	1	0	-0.652200	0.625729	2.530605
11	1	0	-1.598113	1.955458	1.871641
12	6	0	-1.160257	-0.444014	-1.845230
13	1	0	-0.444507	-1.183580	-2.213213
14	1	0	-1.388442	0.225544	-2.681196
15	6	0	-2.443206	-1.136180	-1.340901
16	1	0	-2.874353	-1.713113	-2.165080
17	1	0	-2.167022	-1.847892	-0.559494
18	6	0	-3.470050	-0.140470	-0.837189
19	6	0	-2.560829	0.005695	1.685872
20	1	0	-3.068528	0.086779	2.652302
21	1	0	-2.229370	-1.029591	1.580242
22	6	0	-3.525447	0.391384	0.580508
23	1	0	-3.740955	0.594065	-1.590675
24	1	0	-3.832725	1.431469	0.646907
25	6	0	-4.605058	-0.538176	0.076776
26	1	0	-5.566208	-0.076772	-0.131022
27	6	0	-4.732513	-1.945563	0.595498
28	1	0	-3.766441	-2.374899	0.855913
29	1	0	-5.356538	-1.956996	1.495373
30	8	0	-5.293807	-2.825407	-0.386368
31	1	0	-6.169376	-2.492538	-0.613986
32	6	0	2.666596	0.026310	-0.159011

33	6	0	3.596854	-0.365350	-1.116338
34	6	0	2.433950	-0.725728	0.985359
35	6	0	4.305750	-1.539007	-0.914036
36	1	0	3.760603	0.245694	-1.995540
37	6	0	3.166266	-1.893312	1.172603
38	1	0	1.691459	-0.420374	1.712883
39	6	0	4.107040	-2.316490	0.232889
40	1	0	5.033436	-1.855376	-1.653372
41	1	0	2.993325	-2.488812	2.061889
42	6	0	4.907509	-3.570829	0.446312
43	1	0	4.912350	-4.186114	-0.455553
44	1	0	5.946607	-3.323419	0.679849
45	1	0	4.503980	-4.158616	1.271147
46	9	0	1.738625	2.087802	1.769341

TS-MeF/BCN$_1$/AB

```
Zero-point correction=                           0.373049 (Hartree/Particle)
Thermal correction to Energy=                    0.395714
Thermal correction to Enthalpy=                  0.396658
Thermal correction to Gibbs Free Energy=         0.319928
Sum of electronic and zero-point Energies=          -1170.960156
Sum of electronic and thermal Energies=             -1170.937491
Sum of electronic and thermal Enthalpies=           -1170.936547
Sum of electronic and thermal Free Energies=        -1171.013277
```

One imaginary frequency: -354.07i cm-1

Standard orientation:

Center Number	Atomic Number	Atomic Type	Coordinates (Angstroms) X	Y	Z
1	7	0	-1.922987	1.277083	0.214790
2	7	0	-1.346774	1.501372	1.398828
3	6	0	-1.227144	1.914865	-0.736066
4	6	0	-0.565731	3.016974	-0.088630
5	8	0	-0.721369	2.738129	1.235014
6	8	0	0.013778	3.990836	-0.512057
7	6	0	0.659636	0.709971	-0.334717
8	6	0	0.522304	0.393375	0.849533
9	6	0	1.450382	0.682473	-1.580819
10	1	0	0.842266	0.327253	-2.417655
11	1	0	1.779193	1.696589	-1.832446
12	6	0	0.984540	-0.276604	2.071878
13	1	0	0.212049	-0.943816	2.462745
14	1	0	1.187188	0.469868	2.847037
15	6	0	2.263239	-1.068591	1.732637
16	1	0	2.607748	-1.581686	2.636053
17	1	0	2.005144	-1.838222	1.002241
18	6	0	3.369151	-0.167943	1.215570
19	6	0	2.665285	-0.243506	-1.372734
20	1	0	3.247373	-0.266747	-2.299493
21	1	0	2.299235	-1.257702	-1.196735
22	6	0	3.555005	0.222224	-0.236908
23	1	0	3.616268	0.624500	1.916627
24	1	0	3.905914	1.241389	-0.374502
25	6	0	4.552837	-0.690996	0.436375
26	1	0	5.513216	-0.246515	0.681578
27	6	0	4.663180	-2.143110	0.055459
28	1	0	5.132337	-2.708003	0.867956
29	1	0	3.690362	-2.590749	-0.142142
30	8	0	5.420747	-2.318723	-1.148080
31	1	0	6.299037	-1.949748	-0.999992

32	6	0	-2.673094	0.071534	0.061266
33	6	0	-3.670700	-0.208774	0.989000
34	6	0	-2.375395	-0.800513	-0.978521
35	6	0	-4.380906	-1.392918	0.865371
36	1	0	-3.883293	0.493516	1.785666
37	6	0	-3.109717	-1.976300	-1.089416
38	1	0	-1.581648	-0.581131	-1.682713
39	6	0	-4.116915	-2.290225	-0.175698
40	1	0	-5.160327	-1.623032	1.583488
41	1	0	-2.886052	-2.665184	-1.896125
42	6	0	-4.920322	-3.553559	-0.310644
43	1	0	-5.911800	-3.330903	-0.714772
44	1	0	-4.432154	-4.260048	-0.982450
45	1	0	-5.061760	-4.029837	0.661236
46	9	0	-1.567893	1.899869	-2.004174

TS-MeF/BCN$_2$/SB

```
Zero-point correction=                           0.373152 (Hartree/Particle)
Thermal correction to Energy=                    0.395779
Thermal correction to Enthalpy=                  0.396724
Thermal correction to Gibbs Free Energy=         0.319941
Sum of electronic and zero-point Energies=          -1170.960206
Sum of electronic and thermal Energies=             -1170.937579
Sum of electronic and thermal Enthalpies=           -1170.936635
Sum of electronic and thermal Free Energies=        -1171.013417
```

One imaginary frequency: -358.22i cm-1

Standard orientation:

Center Number	Atomic Number	Atomic Type	Coordinates (Angstroms) X	Y	Z
1	7	0	-2.013288	1.219741	0.190764
2	7	0	-1.571992	1.557311	1.407164
3	6	0	-1.398020	1.985491	-0.720604
4	6	0	-1.014154	3.197704	-0.048858
5	8	0	-1.199020	2.893509	1.266147
6	8	0	-0.615075	4.268646	-0.446494
7	6	0	0.488136	0.807661	0.989035
8	6	0	0.653514	1.172111	-0.177881
9	6	0	0.914815	0.068933	2.184871
10	1	0	0.921606	0.720685	3.062292
11	1	0	0.203989	-0.738869	2.389308
12	6	0	1.466433	1.190677	-1.409381
13	1	0	1.663737	2.218306	-1.727200
14	1	0	0.916417	0.704514	-2.222887
15	6	0	2.785910	0.440924	-1.140371
16	1	0	3.383097	0.447348	-2.057581
17	1	0	3.350854	0.989160	-0.382894
18	6	0	2.544456	-0.993243	-0.711138
19	6	0	2.322067	-0.501705	1.919539
20	1	0	2.652806	-1.048582	2.808165
21	1	0	3.010177	0.334157	1.778889
22	6	0	2.335540	-1.434594	0.722735
23	1	0	1.951529	-1.549480	-1.432557
24	1	0	1.625504	-2.250257	0.830132
25	6	0	3.600224	-1.816764	-0.009506
26	1	0	3.674479	-2.854825	-0.316970
27	6	0	4.925448	-1.215691	0.359854
28	1	0	4.815853	-0.205620	0.760055
29	1	0	5.414052	-1.830927	1.122589
30	8	0	5.740050	-1.179188	-0.820069
31	1	0	6.609498	-0.846206	-0.572267

32	6	0	-2.463259	-0.124760	0.008886
33	6	0	-3.462812	-0.605834	0.846542
34	6	0	-1.868500	-0.927376	-0.957459
35	6	0	-3.871423	-1.923874	0.706968
36	1	0	-3.908924	0.045493	1.588268
37	6	0	-2.302593	-2.241727	-1.086581
38	1	0	-1.073703	-0.546553	-1.588306
39	6	0	-3.303938	-2.758390	-0.261826
40	1	0	-4.648971	-2.311235	1.356145
41	1	0	-1.845065	-2.878853	-1.835233
42	6	0	-3.783066	-4.173961	-0.423934
43	1	0	-4.010432	-4.622421	0.544462
44	1	0	-4.698607	-4.197071	-1.021878
45	1	0	-3.035727	-4.785184	-0.930860
46	9	0	-1.632835	1.892203	-2.008800

TS-MeF/BCN$_2$/ST

```
Zero-point correction=                           0.373007 (Hartree/Particle)
Thermal correction to Energy=                    0.395724
Thermal correction to Enthalpy=                  0.396668
Thermal correction to Gibbs Free Energy=         0.318997
Sum of electronic and zero-point Energies=          -1170.960292
Sum of electronic and thermal Energies=             -1170.937575
Sum of electronic and thermal Enthalpies=           -1170.936631
Sum of electronic and thermal Free Energies=        -1171.014302
```

One imaginary frequency: -359.48i cm-1

Standard orientation:

Center Number	Atomic Number	Atomic Type	Coordinates (Angstroms) X	Y	Z
1	7	0	2.065352	-1.094023	0.338415
2	7	0	1.535247	-1.409483	1.525136
3	6	0	1.608030	-1.954117	-0.581681
4	6	0	1.260009	-3.160869	0.118784
5	8	0	1.290225	-2.778447	1.426073
6	8	0	0.990158	-4.280347	-0.253022
7	6	0	-0.523710	-0.859329	0.876673
8	6	0	-0.546296	-1.297144	-0.276609
9	6	0	-1.106344	-0.083629	1.979571
10	1	0	-1.167831	-0.686897	2.888959
11	1	0	-0.459108	0.770942	2.204535
12	6	0	-1.238270	-1.445789	-1.571721
13	1	0	-1.333929	-2.501546	-1.840246
14	1	0	-0.652830	-0.965626	-2.363727
15	6	0	-2.625793	-0.784963	-1.458652
16	1	0	-3.147441	-0.896879	-2.414480
17	1	0	-3.204286	-1.327135	-0.708005
18	6	0	-2.517866	0.689367	-1.115636
19	6	0	-2.505972	0.400044	1.550221
20	1	0	-2.934415	0.992198	2.364858
21	1	0	-3.149150	-0.472278	1.412796
22	6	0	-2.452842	1.245145	0.292065
23	1	0	-1.901739	1.231525	-1.828290
24	1	0	-1.794062	2.103426	0.394841
25	6	0	-3.670689	1.496965	-0.567124
26	1	0	-3.773556	2.502772	-0.961400
27	6	0	-4.988982	0.855794	-0.242795
28	1	0	-5.639396	0.874181	-1.123485
29	1	0	-4.869878	-0.184310	0.067718
30	8	0	-5.596508	1.607632	0.816943
31	1	0	-6.471802	1.240209	0.981399

32	6	0	2.414958	0.275629	0.124482
33	6	0	3.293990	0.879004	1.014412
34	6	0	1.847036	0.977537	-0.933825
35	6	0	3.606319	2.220168	0.837587
36	1	0	3.729006	0.303064	1.822125
37	6	0	2.183072	2.315109	-1.097464
38	1	0	1.150787	0.498668	-1.612516
39	6	0	3.058370	2.956513	-0.216393
40	1	0	4.295568	2.700276	1.523186
41	1	0	1.751210	2.872322	-1.921582
42	6	0	3.383598	4.413927	-0.390314
43	1	0	2.593631	5.032044	0.045814
44	1	0	4.320736	4.668352	0.105887
45	1	0	3.459959	4.673791	-1.447263
46	9	0	1.961290	-1.903491	-1.845047

TS-MeF/BCN$_2$/AT

```
Zero-point correction=                           0.372965 (Hartree/Particle)
Thermal correction to Energy=                    0.395692
Thermal correction to Enthalpy=                  0.396636
Thermal correction to Gibbs Free Energy=         0.319218
Sum of electronic and zero-point Energies=          -1170.959916
Sum of electronic and thermal Energies=             -1170.937188
Sum of electronic and thermal Enthalpies=           -1170.936244
Sum of electronic and thermal Free Energies=        -1171.013662
```

One imaginary frequency: -354.21i cm-1

Standard orientation:

Center Number	Atomic Number	Atomic Type	Coordinates (Angstroms) X	Y	Z
1	7	0	-1.919488	-1.222110	-0.389021
2	7	0	-1.259841	-1.327263	-1.545733
3	6	0	-1.309315	-1.980662	0.531521
4	6	0	-0.620979	-3.020478	-0.187350
5	8	0	-0.670556	-2.590442	-1.478336
6	8	0	-0.092620	-4.050550	0.163809
7	6	0	0.624548	-0.795128	0.409806
8	6	0	0.586598	-0.338750	-0.735404
9	6	0	1.320684	-0.935676	1.703839
10	1	0	0.660634	-0.661174	2.531529
11	1	0	1.610141	-1.980846	1.857718
12	6	0	1.158005	0.444336	-1.838364
13	1	0	0.440352	1.185690	-2.199046
14	1	0	1.384557	-0.218851	-2.679830
15	6	0	2.441894	1.134141	-1.333402
16	1	0	2.871200	1.714364	-2.156129
17	1	0	2.168795	1.842399	-0.547571
18	6	0	3.469617	0.136057	-0.836779
19	6	0	2.563953	-0.024877	1.687202
20	1	0	3.073173	-0.109445	2.652562
21	1	0	2.226669	1.008994	1.588408
22	6	0	3.529425	-0.399390	0.578656
23	1	0	3.739957	-0.594707	-1.593985
24	1	0	3.841242	-1.438357	0.640216
25	6	0	4.607642	0.532106	0.075667
26	1	0	5.567530	0.072841	-0.137236
27	6	0	4.729811	1.936995	0.590837
28	1	0	3.763726	2.344112	0.895986
29	1	0	5.396033	1.958602	1.459536
30	8	0	5.282811	2.745429	-0.457069
31	1	0	5.431899	3.629773	-0.105280

32	6	0	-2.659335	-0.023026	-0.155833
33	6	0	-3.585908	0.377726	-1.113411
34	6	0	-2.424065	0.723307	0.991362
35	6	0	-4.287035	1.555197	-0.908571
36	1	0	-3.753193	-0.230120	-1.994163
37	6	0	-3.149182	1.895367	1.181289
38	1	0	-1.685705	0.410090	1.719738
39	6	0	-4.084051	2.328572	0.240738
40	1	0	-5.012639	1.878161	-1.647191
41	1	0	-2.975264	2.485665	2.073816
42	6	0	-4.868529	3.594025	0.448182
43	1	0	-5.935590	3.373006	0.531596
44	1	0	-4.551313	4.109084	1.355138
45	1	0	-4.740584	4.269963	-0.400307
46	9	0	-1.742895	-2.097575	1.765625

TS-MeF/BCN$_2$/AB

```
Zero-point correction=                           0.372866 (Hartree/Particle)
Thermal correction to Energy=                    0.395650
Thermal correction to Enthalpy=                  0.396594
Thermal correction to Gibbs Free Energy=         0.319385
Sum of electronic and zero-point Energies=          -1170.960007
Sum of electronic and thermal Energies=             -1170.937224
Sum of electronic and thermal Enthalpies=           -1170.936280
Sum of electronic and thermal Free Energies=        -1171.013488
```

One imaginary frequency: -354.07i cm-1

Standard orientation:

Center Number	Atomic Number	Atomic Type	Coordinates (Angstroms) X	Y	Z
1	7	0	-1.922214	1.276951	0.210820
2	7	0	-1.347606	1.504679	1.394941
3	6	0	-1.225579	1.912555	-0.740924
4	6	0	-0.565500	3.016722	-0.095577
5	8	0	-0.722545	2.741285	1.228599
6	8	0	0.013988	3.989752	-0.520890
7	6	0	0.661087	0.708646	-0.334582
8	6	0	0.523239	0.396705	0.850825
9	6	0	1.452604	0.678258	-1.580128
10	1	0	0.845918	0.317993	-2.415851
11	1	0	1.778440	1.692368	-1.835646
12	6	0	0.985970	-0.267748	2.076005
13	1	0	0.214024	-0.933798	2.469888
14	1	0	1.188463	0.482117	2.847924
15	6	0	2.264729	-1.060499	1.738841
16	1	0	2.609710	-1.571449	2.643327
17	1	0	2.006116	-1.831591	1.010399
18	6	0	3.370287	-0.160484	1.219771
19	6	0	2.670663	-0.242891	-1.368388
20	1	0	3.254192	-0.265293	-2.294213
21	1	0	2.308802	-1.258394	-1.190638
22	6	0	3.556755	0.228548	-0.232326
23	1	0	3.615531	0.633603	1.919539
24	1	0	3.904899	1.248346	-0.371572
25	6	0	4.558123	-0.679518	0.444884
26	1	0	5.515180	-0.230407	0.690019
27	6	0	4.672143	-2.127879	0.066012
28	1	0	5.166778	-2.684699	0.868700
29	1	0	3.694680	-2.582652	-0.108082
30	8	0	5.461211	-2.210354	-1.129212
31	1	0	5.613569	-3.141553	-1.323870

32	6	0	-2.671572	0.070677	0.059539
33	6	0	-3.671112	-0.207311	0.986649
34	6	0	-2.373610	-0.802816	-0.978286
35	6	0	-4.381769	-1.390664	0.863953
36	1	0	-3.885265	0.497337	1.780804
37	6	0	-3.108976	-1.978676	-1.088344
38	1	0	-1.580044	-0.584540	-1.683015
39	6	0	-4.116274	-2.290825	-0.174970
40	1	0	-5.163894	-1.618273	1.580070
41	1	0	-2.886222	-2.667668	-1.895125
42	6	0	-4.913478	-3.559481	-0.297197
43	1	0	-5.960663	-3.332976	-0.513445
44	1	0	-4.526622	-4.192212	-1.096218
45	1	0	-4.887876	-4.122360	0.638486
46	9	0	-1.564954	1.894368	-2.009369

References:

1. Plougastel, L.; Koniev, O.; Specklin, S.; Decuypere, E.; Créminon, C.; Buisson, D.-A.; Wagner, A.; Kolodych, S.; Taran, F. 4-Halogeno-sydnones for fast strain promoted cycloaddition with bicyclo-[6.1.0]-nonyne *Chemical Communications*, **2014**, *50*, 9376-9378. https://doi.org/10.1039/C4CC03816A
2. March, J. “Advanced Organic Chemistry”. Wiley-Interscience. **1992**, p. 280.
